# Towards artificial intelligence in mental health by improving schizophrenia prediction with multiple brain parcellation ensemble-learning

**DOI:** 10.1038/s41537-018-0070-8

**Published:** 2019-01-18

**Authors:** Sunil Vasu Kalmady, Russell Greiner, Rimjhim Agrawal, Venkataram Shivakumar, Janardhanan C. Narayanaswamy, Matthew R. G. Brown, Andrew J Greenshaw, Serdar M Dursun, Ganesan Venkatasubramanian

**Affiliations:** 1 Alberta Machine Intelligence Institute, Department of Computing Science, University of Alberta, Edmonton, AB Canada; 2 Department of Psychiatry, University of Alberta, Edmonton, AB Canada; 3 The Schizophrenia Clinic, Department of Psychiatry, National Institute of Mental Health and Neuro Sciences, Bangalore, India; 4 Translational Psychiatry Laboratory, Neurobiology Research Centre, National Institute of Mental Health and Neuro Sciences, Bangalore, India

## Abstract

In the literature, there are substantial machine learning attempts to classify schizophrenia based on alterations in resting-state (RS) brain patterns using functional magnetic resonance imaging (fMRI). Most earlier studies modelled patients undergoing treatment, entailing confounding with drug effects on brain activity, and making them less applicable to real-world diagnosis at the point of first medical contact. Further, most studies with classification accuracies >80% are based on small sample datasets, which may be insufficient to capture the heterogeneity of schizophrenia, limiting generalization to unseen cases. In this study, we used RS fMRI data collected from a cohort of antipsychotic drug treatment-naive patients meeting DSM IV criteria for schizophrenia (*N* = 81) as well as age- and sex-matched healthy controls (*N* = 93). We present an ensemble model -- EMPaSchiz (read as ‘Emphasis’; standing for ‘Ensemble algorithm with Multiple Parcellations for Schizophrenia prediction’) that stacks predictions from several ‘single-source’ models, each based on features of regional activity and functional connectivity, over a range of different a priori parcellation schemes. EMPaSchiz yielded a classification accuracy of 87% (vs. chance accuracy of 53%), which out-performs earlier machine learning models built for diagnosing schizophrenia using RS fMRI measures modelled on large samples (*N* > 100). To our knowledge, EMPaSchiz is first to be reported that has been trained and validated exclusively on data from drug-naive patients diagnosed with schizophrenia. The method relies on a single modality of MRI acquisition and can be readily scaled-up without needing to rebuild parcellation maps from incoming training images.

## Introduction

Despite decades of research, there are no precise and reliable etiopathophysiological markers for major psychiatric conditions.^1^ Impeding factors range from inherent challenges in studying complex genetic disorders^2^ to weakly established neural bases for cognition, experience and behaviour.^3,4^ However, a part of the problem is a mismatch between current diagnostic standards for psychiatric illnesses and observations emerging from basic systems and behavioural neuroscience research.^5^ Recognized biological heterogeneity, also adds to the difficulty of identifying reliable biological markers associated with these conditions.^6^ Treatments for psychiatric disorders have emerged largely as a result of serendipitous observations^7^ with an unfortunate range of side-effects^8^ and this may be why mortality and prevalence rates associated with psychiatric illnesses have not decreased in past years,^9^ as compared to other medical conditions such as certain types of cancer^10^ or heart diseases.^11^

In particular, the underlying pathophysiology of schizophrenia, a severe and debilitating psychotic illness, still remains elusive, with few established consistent findings.^12^ Currently objectively measurable diagnostic tests for schizophrenia^13^ are lacking, and the reliability of diagnoses based on observable signs and symptoms leaves room for improvement.^5^ Further, there is marked heterogeneity within clinical manifestations of ‘schizophrenia’ as well as considerable overlap with other psychiatric diagnoses, leading many to question the validity of a singular disease entity.^14^

In this context, applying machine learning techniques to MRI data has the potential to provide an objective and evidence-based approach for identification and management of schizophrenia.^15,16^ Machine-learned MRI models have the potential to identify biological markers and delineate symptom clusters. Recently, an increasing number of studies have attempted to classify schizophrenia (vs. healthy controls) based on functional alterations in resting-state brain patterns (Table 1, see supplementary materials for more description of these studies).

**Table 1:** List of single-site studies that provided machine learning model for predicting schizophrenia using resting-state brain patterns

Study	Year	Total: Size of classes	Accuracy
Shen et al.^83^	2010	52: 32 SCZ, 20 HC	86.50%
Fan et al.^84^	2011	62: 31 SCZ, 31 HC	87.1%^a^
Yu et al.^85^	2013	89: 32 SCZ, 38 HC (+19 MDD)	80.9%
Anderson and Cohen^86^	2013	146: 74 SCZ, 72 HC (COBRE dataset)	65%
Arbabshirani et al.^87^	2013	56: 28 SCZ, 28 HC	96%^a^
Yu et al.^88^	2013	71: 24 SCZ, 25 healthy siblings of SCZ, 22 HC	62%
Guo et al.^89^	2014	131: 69 SCZ, 62 HC	80%
Brodersen et al.^90^	2014	83: 41 SCZ, 42 HC	78%^a^
Anticevic et al.^91^	2014	180: 90 SCZ, 90 HC	73.9%
Watanabe et al.^92^	2014	123: 54 SCZ, 67 HC	73.50%
Chyzhyk et al.^93^	2015	54: 26 SCZ with history of AH, 14 SCZ without a history of AH, 28 HC	97.1%^a^
Cheng et al.^94^	2015	48: 19 SCZ, 29 HC	79%
Peters et al.^95^	2016	36: 18 SCZ, 18 HC	91%^a^
Mikolas et al.^96^	2016	126: 63 SCZ with FE SCZ, 63 HC	73%
Cabral et al.^34^	2016	132: 66 SCZ, 66 HC (COBRE dataset)	70.5%
Yang et al.^97^	2016	86: 40 SCZ, 46 HC	77.91%
Iwabuchi and Palaniyappan^98^	2017	133: 62 SCZ, 71 HC	78.04%^a^
Lottman et al.^99^	2017	69: 34 unmedicated (17 drug-naive) SCZ + follow-up post treatment, 35 HC	83.8%^a^
Guo et al.^100^	2017	68: 28 FE drug-naive SCZ, 28 family-based controls, 40 HC	92.86%^a^

*SCZ* Schizophrenia, *HC* Healthy controls, *AH* Auditory hallucinations, *FE* First episode, *MDD* Major depression

^a^Accuracy of best model among several reported models

**Fig. 1:**
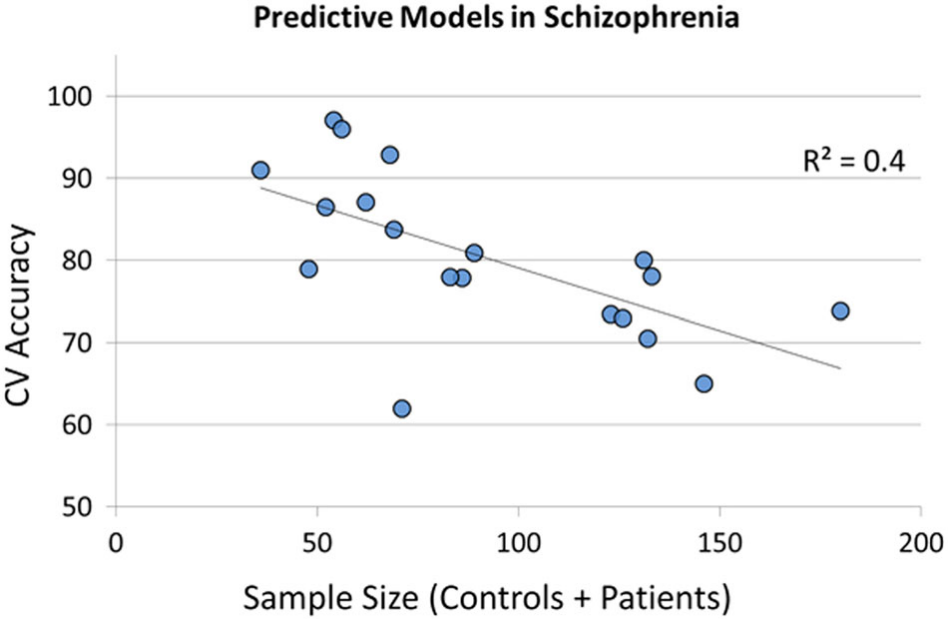
Negative correlation between sample size and cross-validated (CV) accuracy for predicting schizophrenia using resting-state brain patterns (studies cited in Table 1)

Most earlier studies assessed patients already undergoing treatment, which means their fMRI scans were confounded with antipsychotic drug effects^17^ – hence, those scans did not correspond to the point of first medical contact, and so may not lead to optimal diagnostic models. Further, diagnostic models obtained from larger datasets (more than 100 subjects) have classification accuracies well below 80% (Fig. 1). Many have observed this phenomenon: “smaller-N studies reach higher prediction accuracy of schizophrenia with neuroimaging data”.^18^ Even with higher cross-validated accuracy, the smaller samples likely do not capture the heterogeneity of the disease, which suggests that these models will not generalize well to unseen cases.

Many of these studies first parcellate the whole brain resting-state information into spatial regions that are considered homogeneous. However, with the increasing number of parcellation methods and atlases now available, the choice of which parcellation to use seems rather arbitrary. These methods can vary widely in principle and can be based on (a) pre-defined ontology of brain structures such as post-mortem cytoarchitecture,^19,20^ sulco-gyral anatomy,^21,22^ anatomical connectivity using diffusion imaging^23,24^ or (b) data-driven modelling of the functional features in the BOLD signal from resting-state^25^ or task-based fMRI^26,27^ or even meta-analyses^28,29^ using analytical techniques such as hierarchical clustering^30^ or independent components analysis.^31^ The quality of the brain network obtained and the downstream predictive model may be largely influenced by the selection of the atlas or parcellation used.^32,33^ Brain segmentations based on these parcellation schemes not only provide a way to reduce the dimensionality of fMRI data but can also provide an elegant way to incorporate prior neurobiological knowledge to ‘refine’ the features. However, to date, there has been no investigation on whether combined learning from multiple predefined parcellation schemes can provide better performance for diagnostic prediction of schizophrenia.

In this study, we eliminated the potential confound of antipsychotic treatment by using resting state fMRI data collected from a cohort of *antipsychotic-naive* schizophrenia patients (*N* = 81) as well as age- and gender-matched healthy controls (*N* = 93). The aim of our study was to improve accuracy for diagnostic prediction, compared to results reported in the literature, by designing a feature creation and learning pipeline that incorporates prior knowledge of neuroanatomy and neurophysiology. Our overall model involves stacking predictions from several single-source models, each based on the specific set of features related to regional fMRI activity and functional connectivity, and a specific a priori parcellation scheme. We demonstrate that our ensemble model yields a classification accuracy of 87% (vs. 53% chance), which is better than any standard single-source model considered in the study. To the best of our knowledge, (1) the performance of our model, based on 174 subjects, outscores earlier machine learning models built for diagnosing schizophrenia using resting-state fMRI measures that have been learned from datasets of *N* > 100 subjects; and (2) this is the only such classification model that has been built and validated exclusively on never-treated schizophrenia cases.

Our method relies on a single modality of data acquisition for neuroimaging and is easily scalable as it uses a set of pre-defined atlases—i.e., it does not rely on data-driven brain parcellation methods, such as group-independent component analysis.

## Results

We show below that (a) our EMPaSchiz ensemble learner, which learns a combination of learned classifiers, each trained on its own neuroimaging feature extractions and brain parcellation schemes, produces a classifier that can predict schizophrenia more accurately than any of the individual predictors (that used just a single feature/parcellation combination). (b) Within this ensemble prediction framework, even a very small fraction of features (as low as top 0.5% selected via univariate tests) can still provide high prediction accuracy (>80%). (c) This learning framework can also produce models that can distinguish clinically symptomatic versus non-symptomatic patients, with moderate accuracy.

**Fig. 2:**
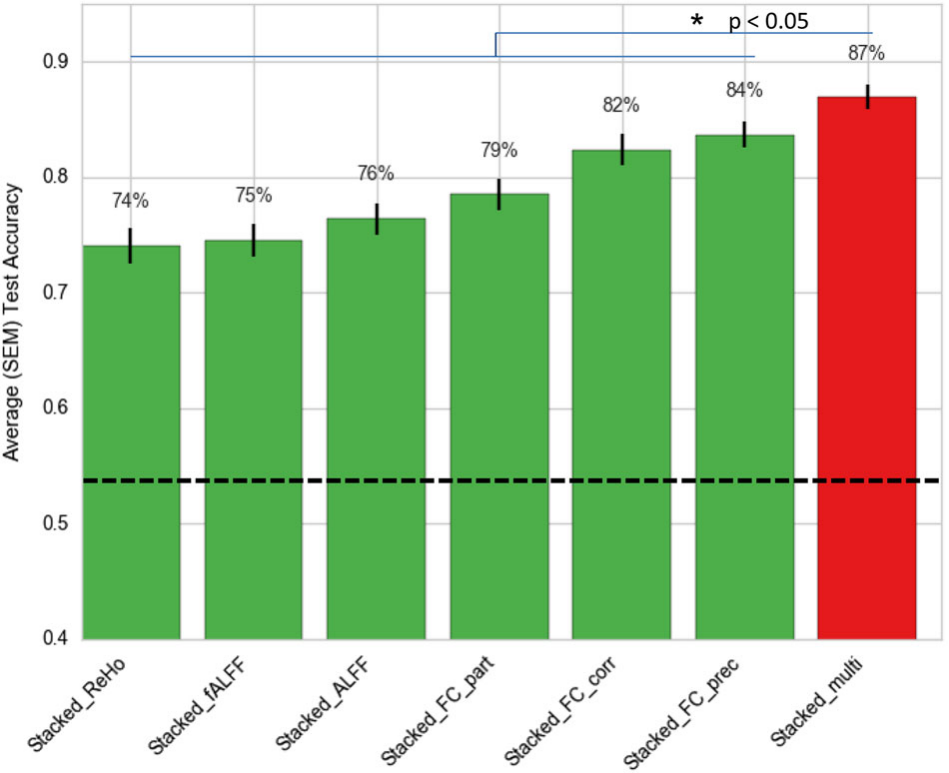
Comparison of 5×10-fold cross-validation prediction accuracies for stacked learners in EMPaSchiz model. The comprehensive ensemble of EMPaSchiz “stacked-multi” is shown in red. Stacked-multi shows the best performance and performs significantly better than all other stacked models (all *p* < 0.05). The dotted line is the majority class baseline predictor. SEM standard error of mean

Table 2 presents the 5 × 10-fold cross-validation prediction performance of the various learners in EMPaSchiz. Majority class baseline accuracy for schizophrenia prediction (declaring every subject to be control) was 53.4% (93 controls of 174 total subjects). These accuracy values are plotted in Fig. 2. Stacked models with neuroimaging features that are regional—viz., ALFF, fALFF and ReHO—had accuracies in the range of 74 to 76%, while the ones based on functional connectivity—viz., FC-Correlation, FC-partial correlation, FC-precision—showed better performance with 79 to 84% accuracy. The final ensemble model EMPaSchiz (stacked-multi) showed the best performance with accuracy of 87%, sensitivity of 80%, specificity of 93% and precision of 92%, each with standard errors of 1–2%. This accuracy of stacked-multi was significantly better than second best stacked model (stacked-FC-precision at 84%, *t*-test, *p* = 0.03).

**Table 2:** Model performance (in percentage) and elements of confusion matrix of the various stacked learners in EMPaSchiz model: average (standard errors) − 5 × 10-fold CV

	Accuracy	Precision	Sensitivity	Specificity	True positive	True negative	False positive	False negative
Stacked-multi	86.9 (1.1)	91.9 (1.4)	79.8 (1.8)	93.1 (1.2)	65.0 (1.4)	86.8 (1.2)	6.2 (1.1)	16.0 (1.4)
Stacked-ALFF	76.4 (1.4)	76.3 (1.8)	73.9 (2.2)	78.7 (1.9)	59.8 (1.7)	73.0 (1.7)	20.0 (1.9)	21.2 (1.8)
Stacked-ReHo	74.1 (1.6)	73.4 (2.0)	74.6 (2.0)	73.6 (2.5)	60.4 (1.6)	68.2 (2.3)	24.8 (2.5)	20.6 (1.6)
Stacked-fALFF	74.5 (1.5)	73.8 (1.7)	72.2 (1.8)	76.6 (1.9)	58.6 (1.6)	72.0 (1.7)	21.0 (1.7)	22.4 (1.7)
Stacked-FC-correlation	82.4 (1.3)	83.9 (1.9)	79.7 (1.8)	84.7 (2.0)	64.6 (1.5)	78.8 (2.0)	14.2 (1.9)	16.4 (1.4)
Stacked-FC-partial correlation	78.5 (1.4)	93.7 (1.5)	58.2 (2.8)	96.2 (0.9)	46.8 (2.4)	89.8 (1.0)	3.2 (0.8)	34.2 (2.3)
Stacked-FC-precision	83.7 (1.2)	90.2 (1.6)	73.8 (2.0)	92.3 (1.3)	60.0 (1.9)	86.8 (1.3)	6.2 (1.2)	21.0 (1.8)
Baseline^a^	51.2 (0.3)	47.0 (0.5)	40.7 (0.6)	60.2 (0.5)	33.0 (0.4)	56.0 (0.5)	37.0 (0.5)	48.0 (0.5)

^a^Baseline results are based on permutation test over the randomly shuffled labels (based on 100 repetitions of entire ‘learning with subsequent 10-fold CV evaluations’)

**Fig. 3:**
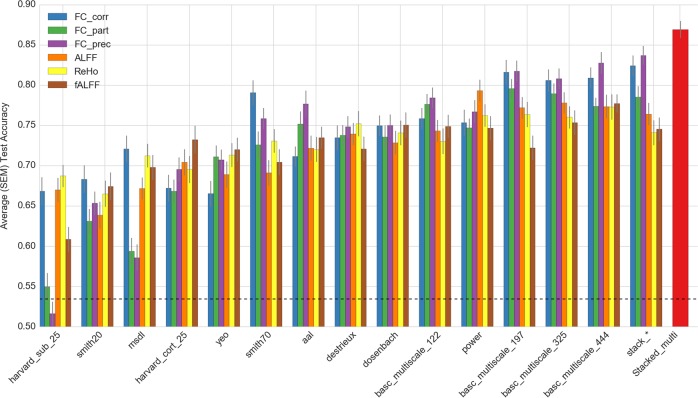
Comparison of 5×10-fold cross-validation prediction accuracies for single-source and multi-source models. The comprehensive ensemble model of EMPaSchiz “stacked-multi” is shown in red. (Horizontal dotted line, at 0.53, is the majority class baseline predictor. SEM Standard error of mean)

Figure 3 shows a comparative profile of accuracies for various SSM predictors along with EMPaSchiz stacked models. (Supplementary material provides results in tabular format as well as plots of comparisons limited to specific feature types. It also provides results for various ensemble learners that were stacked parcellation-wise.) Prediction accuracies for SSM ranged from 52% (FC-precision with harvard_sub_25) to 83% (FC-precision with basc_multiscale_444) and averaged overall at 73%. In general, basc_multiscale atlases showed better performance than the others. For instance, accuracies of EMPaSchiz stacked models were comparable to basc_multiscale_197 models for FC-correlation at 82% and for FC-partial correlation at 79%.

**Fig. 4:**
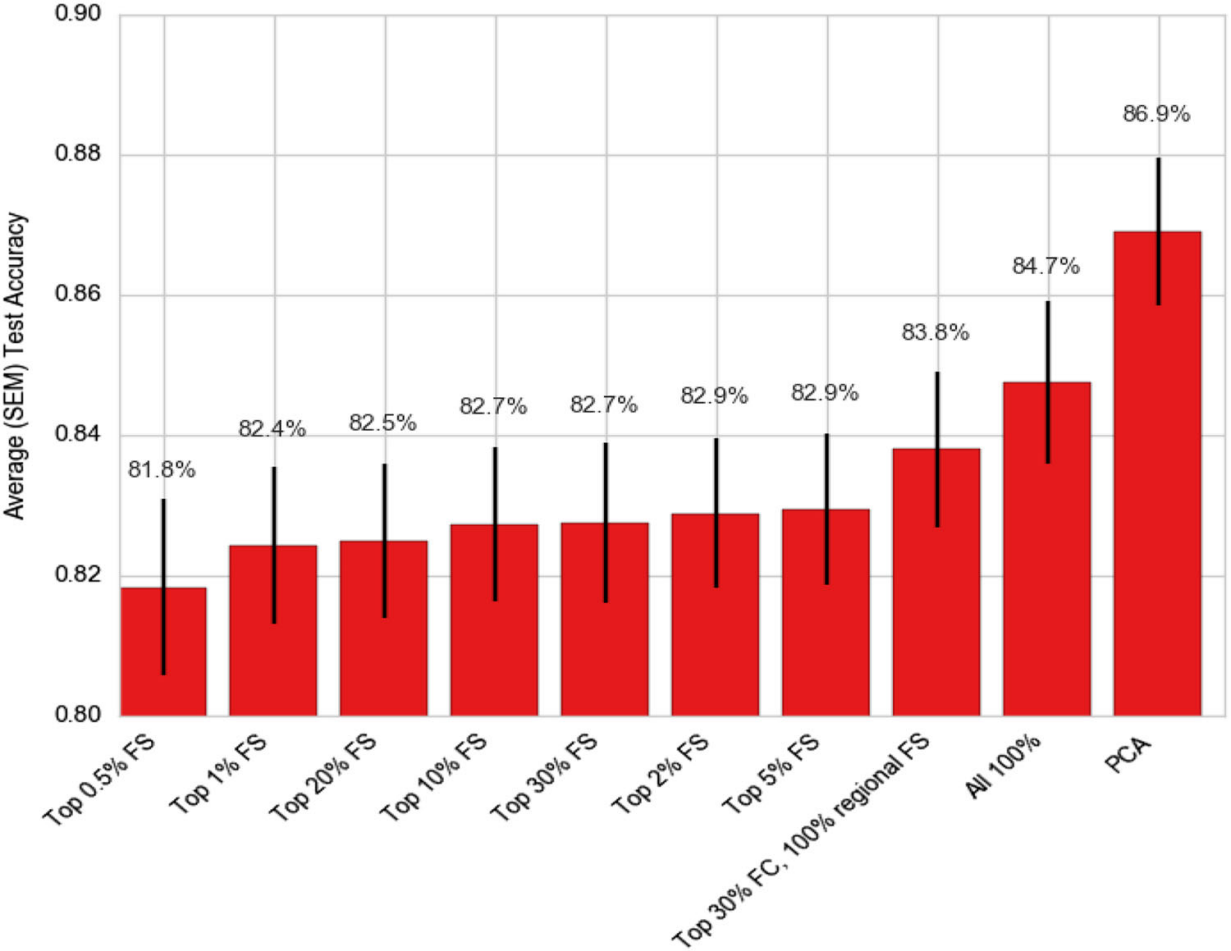
Comparison of 5×10-fold cross-validation prediction accuracies for stacked-multi models with various levels of feature selection and PCA

We examined the effect of feature selection using top-*r* percentage of total features based on a univariate test, of *r* percentile of the highest *F*-value scores, for *r* = 0.5%, 1%, 2%, 5%, 10%, 20%, 30%, as well as “all regional features +30% connectivity features” (we chose this combination as, for any given parcellation, the number of regional features was much less than that of connectivity features), and all features (no feature selection). Note that each “setting” is applied to all 84 SSMs. Figure 4 shows the comparative profile of model performances with varying levels of *r* for top-*r* percentage of features, along with original EMPaSchiz (stacked-multi) model where feature reduction was done using PCA. (Supplementary materials provide results in tabular format as well as additional plots of comparisons of feature selection methods for SSM and MSM models.) Using all features (*r* = 100%, i.e., no selection/reduction) showed accuracy of 85% (which was slightly poorer than PCA reduced features at 87% but was not a statistically significant difference) and accuracy declined only slightly when *r* was reduced gradually to as low as 0.5. It is noteworthy that with only 0.5% of top features, our ensemble prediction framework still showed a high prediction accuracy of 82%.

Patients with schizophrenia in our sample showed a range of psychopathological symptom severity, as measured using the clinical scales SANS for negative symptoms (integer values from 0 to 110) and SAPS for positive symptoms (integer values from 8 to 55). We used the first and last quartile of these scales to categorize the 20 least, and the 20 most, severely symptomatic patients. We then used our ensemble prediction framework in leave-one-out cross-validation setup to predict the high-symptomatic patients against non/low-symptomatic ones (majority class baseline accuracy of 50%). We used leave-one-out cross-validation (rather than 10-fold) to deal with low number of subjects (*N* = 40) that were available for this analysis. Prediction accuracy for stacked-multi model was 73.2% for SANS and 61.9% for SAPS of schizophrenia psychopathology.

**Fig. 5:**
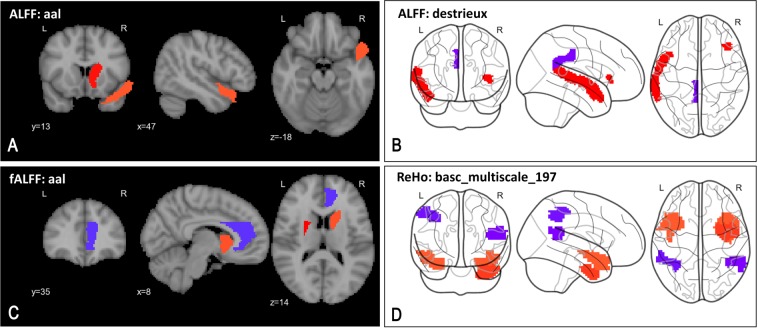
Key pathological alterations in schizophrenia suggested by top-most reliable features—elevated (red) and suppressed (blue) changes in regional activity. Panels show top 98th percentile of top regional features. **a** Higher ALFF in right caudate and right superior temporal pole (aal). **b** Higher ALFF in lateral aspect of left superior temporal gyrus and horizontal ramus of the right lateral sulcus, and lower ALFF in left posterior-dorsal cingulate gyrus (destrieux). **c** Higher fALFF in left putamen, right caudate and lower fALFF in right anterior cingulum (aal). **d** Higher ReHo in left superior temporal pole, right inferior temporal gyrus, and lower ReHo in left inferior parietal lobule and right superior temporal gyrus (basc_multiscale_197)

**Fig. 6:**
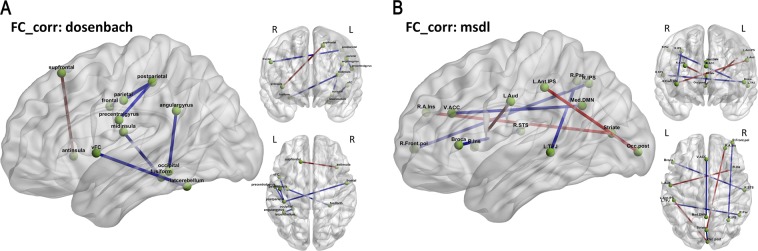
Key pathological alterations in schizophrenia suggested by top-most reliable features—network edges show elevated (red) and suppressed (blue) changes in functional connectivity. Panels show top 99th percentile of top functional connectivity features using dosenbach and msdl atlases. **a** Decreased functional connectivity between regions—left ventral frontal cortex and left lateral cerebellum, left occipital and left angular gyrus, left middle insula and right fusiform gyrus, and lastly left post parietal cortex with three nodes namely right frontal gyrus, left parietal, left precentral gyrus. Increased interhemispheric functional connectivity between left superior frontal gyrus and the right anterior insula. **b** Decreased functional connectivity between regions—striatum and posterior occipital lobe, right intraparietal sulcus and right frontal pole, ventral anterior cingulate cortex and medial default mode network, left temporo-parietal junction and right parietal cortex, right superior temporal sulcus and Broca’s area. Increased functional connectivity between regions—right anterior insula and striatum, right insula and left auditory cortex, and left anterior intraparietal sulcus and posterior occipital lobe

To identify some of the key pathological alterations in our schizophrenia sample, we estimated the reliability of a feature’s importance for diagnostic prediction, similar to the approach used by an earlier neuroimaging study^34^ – sorting the features by their respective mean logistic regression weight divided by its standard error for each feature in a particular learned SSM generated during 50 folds of cross-validation. (This was performed with raw ROI data, without any PCA transformations.) Fig. 5 (respectively Fig. 6) highlight some of the top-most ( > 98 or 99th percentile) reliable features using representative atlases for regional resting state measures (respective connectivity).^35^ However, given the complexity of our ensemble model (which recall is based on 84 SSM), these depictions should be considered just representative in nature, and cannot be claimed as the ‘only’ important features in the model.

The pattern of functional connectivity changes (Fig. 6) indicates robust hypo-connectivity between the frontoparietal network (such as post parietal) and the sensorimotor network (such as frontal, parietal, precentral gyrus) with widespread hypo-connectivity in language (e.g.: Broca), attention (e.g.: frontal pole, parietal) and default mode network (e.g.: angular, fusiform gyrus). On other hand, the auditory network as well as the anterior insula, which is implicated in high-level cognitive control, attentional processes and saliency,^36^ show hyper-connectivity. Similarly, the overall picture (Fig. 5) shows increased regional low frequency activity in the superior temporal gyrus and basal ganglia structures - caudate, putamen, and reduced regional activity in cingulum.

## Discussion

This study aimed to build a machine learned classifier for diagnosing schizophrenia that depends on a single neuroimaging modality of acquisition - resting state fMRI. Resting state fMRI is a popular imaging method and possibly better than task-based fMRI, since the latter depends on experimental parameters that require standardization. Further, resting state fMRI is not limited by participants’ attention or cognitive ability to perform a task and hence is applicable to patients with more pronounced disabilities.^37^

Several recent studies have built diagnostic models using data from patients receiving antipsychotic drug treatment (see Table 1). However, antipsychotics are known to affect brain activity and function,^38,39^ and a recent study cautions against the practice of interpreting brain changes in a medicated state, noting it might not be related to the pure pathology of schizophrenia.^17^ We developed the model presented in this study on a sample of never-treated schizophrenia patients, to make our results directly apply to realistic clinical scenarios of diagnosis at first clinical presentation. Further work will be necessary to examine how this may generalize to medicated patients, as well as other confounds, such as multi-site batch effects, remains to be examined.^40^ It is notable, however, that non-medicated patients are an important group for analysis and represent, perhaps, the most difficult sample for recruitment. In this way our study provides a very important sample to demonstrate the value of our approach.

With respect to diagnostic accuracy of schizophrenia, Schnack and others have observed that smaller sample studies may reach high prediction accuracy at the cost of lower generalizability to external samples -- an effect attributed to clinical heterogeneity, physiological variation, sampling noise and errors in diagnosis.^18^ In our outline of recent literature on machine learning studies with resting-state fMRI (see the Introduction section), we also observed this relation (see Fig. 1). Nevertheless, our ensemble model outscores earlier models built for diagnosing schizophrenia using resting state fMRI measures, even though it was learned from a large sample. We believe this may be because our feature creation process incorporates prior rich neurobiological knowledge with simultaneous use of regional and connectivity measures that are jointly extracted over various biologically-informed brain atlas schemes. We demonstrate that if we employ standard machine learning pipelines (called SSM here) on this dataset of untreated patients, we obtain a level of performance ( < 80% accuracy) that is similar to the results reported widely in earlier studies with comparable sample sizes. Hence, these drug-naive cases are unlikely to be ‘easier’ to model than standard treated cases. Our results provide encouraging progress toward deploying automated or semi-automated diagnostic systems based on neuroimaging and predictive models in psychiatric clinics. However, the performance of our model is favoured by the fact that the entire sample in this study comes from a single site, meaning it does not need to deal with the challenges of cross-site generalizability and site-specific effects. Future clinical studies with larger cohorts, preferably from multiple clinical sites, would be necessary to justify clinical deployment.

Our EMPaSchiz model used brain parcellations that were based on prior knowledge of anatomy / cytoarchitecture or statistical maps extracted from correlation structure in fMRI data collected and analysed in earlier studies. Hence, these maps might not perfectly adapt to signals in the individual subject images – which might not be an issue for data-driven parcellation or clustering techniques. Our study neither explored that option, nor compared model performance empirically, with features obtained with these two alternative methodologies. However, use of pre-existing parcellations reduces chances of overfitting, and possibly increases the robustness of the resulting model. Note also that these *a priori* ROIs incorporate nicely biological knowledge of fMRI data into the feature creation process, which can help interpretation of results, and provide an effective way to reduce dimensionality. Our model may be readily scaled-up with relatively little computation, as it does not need to build parcellation maps from incoming training images.

It is often challenging to provide a biological interpretation of complex machine learning models, as the goal of the learning process is to find a model that maximizes prediction performance, which may require (possibly non-linear) combinations of thousands of features. In this study, we produced an effective classifier by seeking the coefficients for the features that collectively optimize the predictive accuracy. In general, such coefficients need not correspond to the inherent correlation of each individual feature with the outcome. This is especially true in our approach of using multiple parcellations of the brain, as this means the “features” will overlap to a large degree. This can be seen as potential limitation for the interpretation of our model. We provide only a snapshot of some representative changes in patient’s brain, showing only the most reliable resting state features; features that, alone, may be neither necessary nor sufficient to obtain the prediction performance of the reported ensemble model. However, several of these brain networks and regions were observed to be altered consistently in schizophrenia.^41–43^

Functional connectivity aberrations observed in our study are consistent with the dysconnectivity hypothesis of schizophrenia.^44^ This theoretical framework describes schizophrenia as a dysconnection syndrome linking aberrations at the level of synapse with the abnormalities in the long-range connectivity of several brain networks.^45^ A vital component of the dysconnectivity hypothesis is proposed aberrant connectivity between prefrontal cortex and other brain regions, which is posited to give rise to key symptoms such as delusions and hallucinations.^46^ A systematic review of fMRI studies on functional connectivity supports reduction in brain region connectivity in subjects with schizophrenia, especially reductions involving prefrontal cortex,^47^ in agreement with our observations. Our findings of concurrent hyper-connectivity among some regions is also consistent with earlier reports of increased functional connectivity in schizophrenia.^48^ Another core postulate of the dysconnectivity hypothesis is that modulation of synaptic efficacy with resultant fronto-temporo-parietal aberrations leads to hallucinations / delusions in schizophrenia.^49^ The hypothesized synaptic efficacy aberrations may be linked to NMDA receptor abnormalities.^49^ In this context it is of interest that effects on temporoparietal-prefrontal circuitry through transcranial Direct Current Stimulation (possibly via NMDA-dependent mechanisms^50^) has been shown to ameliorate severity of auditory hallucinations,^51,52^ possibly through “correction” of functional dysconnectivity.^53^ It is likely that further systematic application of machine learning techniques to analysis of brain connectivity may be useful for developing prognostic markers for schizophrenia that might predict differential responses to clinical interventions.

A general conceptual limitation of machine learning studies in psychiatry is that the diagnostic labels might themselves be ill defined. Amidst an ever-expanding volume of research data, inconsistencies in neurobiological findings fuel doubts about the validity of the currently defined disease construct of schizophrenia. This might be an issue inherent in psychiatric practice, which contributes to low reliability of diagnosis with nosology such as the DSM criteria. The work reported here may indicate a useful step towards more biological informed diagnoses, as it involves developing algorithms to predict current psychiatric diagnoses based on objective neurobiological features. This approach could also provide us with a framework for evaluating the validity of clinical diagnoses. Lastly, our empirical results show that multi-parcellation ensemble learning models may effectively learn models for early diagnosis of schizophrenia; we anticipate that this approach may work for other psychoses, and for prediction of treatment responses.

## Methods

### Subjects

This study examined 92 patients attending the clinical services of the National Institute of Mental Health & Neurosciences (NIMHANS, India), who fulfilled DSM-IV criteria for schizophrenia and were never treated with any psychotropic medications including antipsychotics. The diagnosis of schizophrenia was established using the Mini International Neuropsychiatric Interview (MINI) Plus,^54^ which was confirmed by another psychiatrist through an independent clinical interview. The details related to illness onset and antipsychotic-naive status were carefully ascertained by reliable information obtained from at least one additional adult relative. The Scale for Assessment of Positive Symptoms (SAPS) and Scale for Assessment of Negative Symptoms (SANS) were used to measure psychotic symptoms.^55^ Clinical assessments and MRI were performed on the day before starting antipsychotics.

Controls were recruited from among the consenting healthy volunteers from the same locale to match for age and sex. We used 102 age- and sex-matched healthy volunteers, who were screened to rule out any psychiatric diagnosis using the MINI as well as a comprehensive mental status examination. For both cases and controls, we recruited only right-handed subjects to avoid the potential confounds of differential handedness. None of the study subjects had contraindications to MRI or medical illness that could significantly influence CNS function or structure, such as seizure disorder, cerebral palsy, or history suggestive of delayed developmental milestones. There was no history suggestive of DSM-IV psychoactive substance dependence or of head injury associated with loss of consciousness longer than 10 min. No subjects had abnormal movements as assessed by the Abnormal Involuntary Movements Scale. Pregnant or postpartum females were not included. The supplementary material provides a table with details of demographic and clinical profile of 174 subjects who qualified to be included in the study. (See details on excessive head movement in the ‘Image pre-processing’ section)

The catchment area for the subject recruitment involved the southern states of India. We obtained informed written consent after providing a complete description of the study to all the subjects. The NIMHANS ethics committee reviewed and approved the original research protocol. The Research Ethics Board at University of Alberta, Edmonton approved the secondary analysis of archived data.

### Image acquisition

Magnetic Resonance Imaging (MRI) was done in a 3.0 Tesla scanner (Magnetom Skyra, Siemens). Resting State Functional MRI: BOLD (Blood Oxygen Level Dependent) sensitive echo-planar imaging was obtained using a 32-channel coil for a duration of 5 minutes 14 s, yielding 153 dynamic scans. The scan parameters were: TR = 2000ms; TE = 30ms; flip angle = 78°; Slice thickness = 3 mm; Slice order: Descending; Slice number = 37; Gap = 25%; Matrix = 64 × 64 × 64 mm^3^, FOV = 192 × 192, voxel size = 3.0 mm isotropic. Subjects were asked to keep their eyes open during the scan. For intra-subject co-registration, structural MRI: T1-weighted three-dimensional high-resolution MRI was performed (TR = 8.1 msec, TE = 3.7ms, nutation angle = 8°, FOV = 256 mm, slice thickness = 1 mm without inter-slice gap, NEX = 1, matrix = 256 × 256) yielding 165 sagittal slices.

### Image pre-processing

We performed pre-processing and feature extraction using MATLAB (The MathWorks, Inc) toolboxes including Statistical parametric mapping (SPM8, http://www.fil.ion.ucl.ac.uk/spm), Data Processing Assistant for Resting-State fMRI (DPARSF)^56^ as well as Python toolboxes including the nilearn package^57^ based on scikit-learn, a Python machine learning library.^58^ We checked acquired images visually for artefacts such as incomplete brain coverage or ghosting; then re-orientated the origin to the anterior commissure in structural MRI and fMRI images. The first ten volumes of each functional time-series were discarded as they were before the time required for the scanner field to reach steady magnetization, and for the participants to adapt to scanning noise. Images were then pre-processed with slice-timing correction, image realignment to correct for motion, and intensity normalization. Since head movement may lead to group-related differences,^59–61^ we excluded images for 11 patients and 9 controls from the study based on excessive head movement (translational > 2.0 mm and/or rotational > 2°).^62^ This yielded a total of 174 subjects: 93 controls and 81 patients. Functional images were co-registered with the structural image and then normalized to MNI space resampled to 3×3×3 mm^3^. Nuisance regression was performed to remove noise in the signal induced by head motion using 24 regressors derived from the parameters estimated during motion realignment, scanner drift using a linear term, as well as global fMRI signals from white matter and cerebrospinal fluid segments using SPM’s new segment method.^63^ Normalized images were smoothed, detrended and band-pass filtered as appropriate—depending on the feature to be extracted, see details below.

### Feature extraction

To obtain neurobiologically relevant features, we projected each resting brain information into 14 different parcellations, each based on a specific a priori defined atlas or set of regions of interest (ROIs). Our goal here was to jointly learn from this entire set of neuroimaging features extracted through several brain parcellation schemes to obtain an accurate model; *n.b*., we are neither trying to compare nor evaluate the influence of any single feature type or ROI definition on prediction accuracy. Our goal is to produce a predictive model whose validation is only its predictive accuracy.

We used the following 14 pre-defined brain parcellation schemes:

- **yeo**: intrinsic functional connectivity of cerebral cortex^25^
- **smith20, smith70**: functional networks during activation and rest (at two different resolutions)^26^
- **harvard_cort_25, harvard_sub_25**: Harvard-Oxford cortical and subcortical parcellation (http://www.cma.mgh.harvard.edu/fsl_atlas.html)
- **msdl**: multi-subject dictionary learning for functional parcellation^64^
- **aal**: macroscopic anatomical parcellation of single-subject brain^65^
- **basc_multiscale_122, basc_multiscale_197, basc_multiscale_325 and basc_multiscale_444**: multi-level bootstrap analysis of stable clusters in resting-state fMRI, at four different resolutions^66^
- **destrieux**: sulcal depth-based anatomical parcellation of the cerebral cortex^67^
- **dosenbach**: multivariate pattern analysis of functional connectivity^28^
- **power**: graph measures of functional brain organization^68^

For each of these 14 parcellation schemes, we extracted 3 regional-based and 3 connectivity-based resting brain fMRI features. For regional features, we used:

- **ALFF**: amplitude of frequency fluctuations
- **fALFF**: fractional ALFF
- **ReHo**: regional homogeneity

We smoothed each functional image using a 4 mm FWHM gaussian kernel (except for extraction of ReHo - to avoid overestimation of spatial homogeneity) and band-pass-filtered fMRI time-courses at 0.01–0.08 Hz to capture slow fluctuations that are believed to reflect spontaneous brain activity.^69,70^ ALFF was calculated as total power within the frequency range between 0.01 and 0.08 Hz to estimate the strength of low frequency oscillations.^71^ fALFF was calculated as power within the low-frequency range (0.01–0.08 Hz) divided by the total power in the entire detectable frequency range.^69^ Lastly, ReHo was calculated using Kendall’s coefficient of concordance,^72^ as a measure of the similarity between the time series of a given voxel and its nearest neighbours.^73^

We calculated each of these features at the voxel level using the DPARSF toolbox, standardized and then averaged over an ROI. For each ROI, we ran a nuisance regression across the features to remove the effects of confounding variables that are generally recommended and commonly reported in neuroimaging research—age, sex, and total intracranial volume.^74^ In addition, we also used average framewise displacement to (at least partially) counter systematic yet spurious correlations in functional connectivity that may arise from subject motion.^59^

We also computed connectivity features with each of the 14 parcellations, by extracting average time series per ROI and then estimating functional connectivity matrices between each pair of regions using one of three statistical measures

- Pearson correlation
- partial correlation
- precision

In each case, the feature vectors were the flattened lower triangular part of these symmetric matrices.

We chose to study the above features as earlier literature established their relevance to schizophrenia pathology. Abnormalities in low-frequency oscillations^70,75^ and regional homogeneity of blood-oxygen-level-dependent signals^76,77^ have been well documented in schizophrenia. Further, patients diagnosed with schizophrenia have exhibited changes in functional brain connectivity, as revealed through distant correlations.^77,78^ In addition to simple Pearson correlation, we described the connectivity structure using partial correlation, which measures the interactions between two ROIs. We use a sparse precision matrix—i.e., the sparse inverse of the covariance matrix—which reveals the brain regions that appear conditionally independent given all other brain regions.^79^

So, in total, our approach ‘**E**nsemble algorithm with Multiple Parcellations for Schizophrenia prediction’, abbreviated as: EMPaSchiz (read as ‘Emphasis’) – modelled 84 sources of data (14 parcellation schemes×(3 + 3) feature types) per subject; these descriptions ranged in size from 17 to 98,346 values. We used appropriate masker classes^57^ to summarize brain signals from non-overlapping clusters (e.g.: basc_multiscale) or overlapping networks (e.g., smith) or spheres centred at seeds with fixed small radius (e.g.: power). Table 3 presents the total number of features per data source. (The supplementary material presents visualizations of a few representative parcellations, overlaid over an MRI slice.)

**Table 3:** Number of features extracted for regional and connectivity feature-types from each parcellation scheme

Parcellation	Regional	Connectivity
yeo	17	136
smith20	20	190
harvard_sub_25	22	231
msdl	39	741
smith70	70	2415
harvard_cort_25	96	4560
aal	116	6670
basc_multiscale_122	122	7381
destrieux	148	10,878
dosenbach	160	12,720
basc_multiscale_197	197	19,306
power	264	34,716
basc_multiscale_325	325	52,650
basc_multiscale_444	444	98,346

### Prediction and evaluation framework

**Fig. 7:**
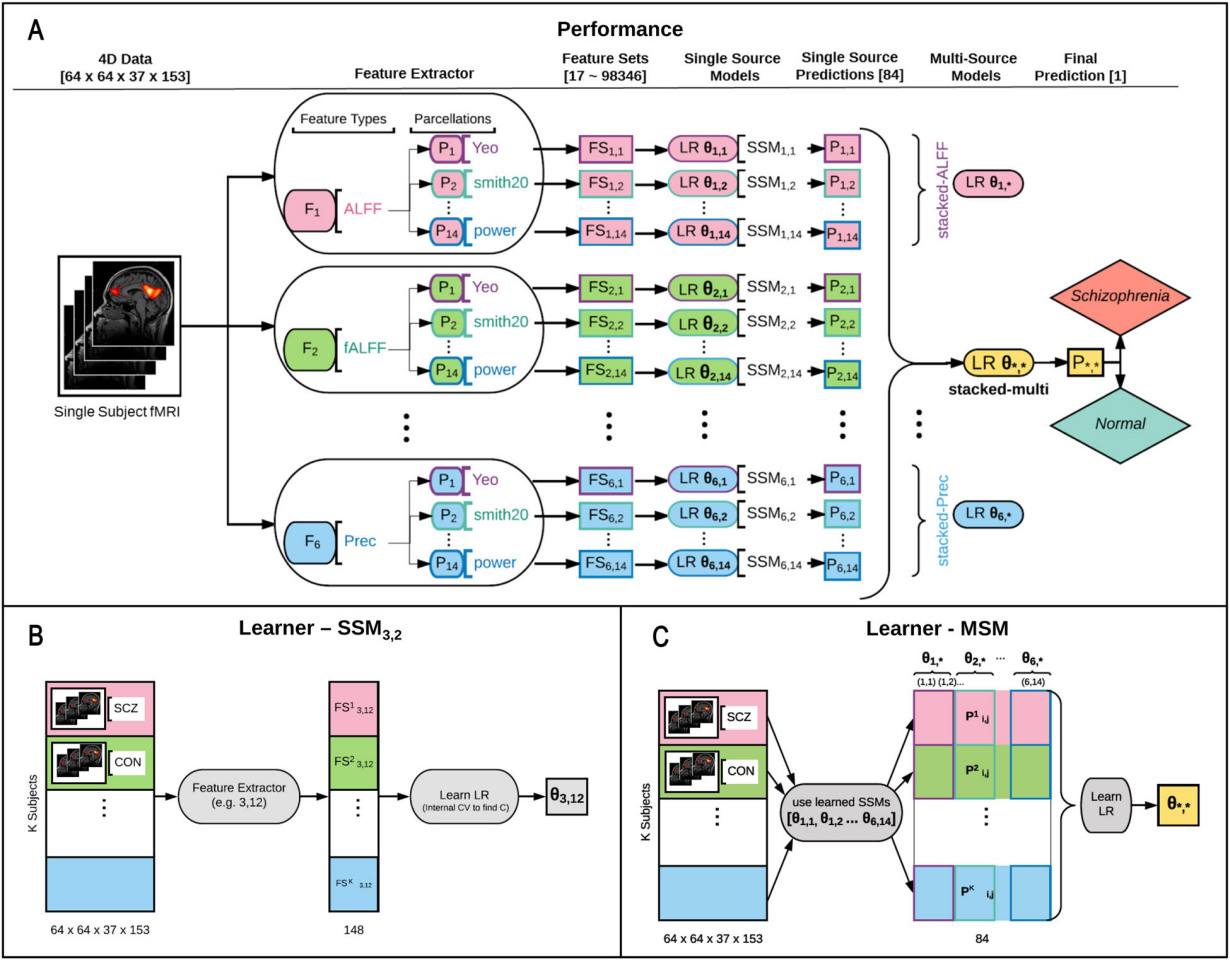
Schematic representation for performance of learned EMPaSchiz model **a** and process of learning that model **b**, **c**—see main text for explanation

**Fig. 8:**
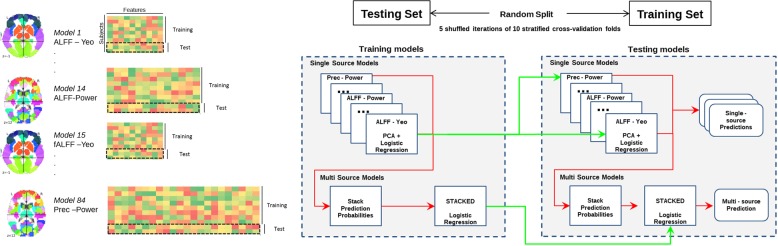
Schematic representation for evaluation of the model with cross-validation. We use 5×10-fold cross-validation to evaluate each learner (the original EMPaSchiz-Learner, and its six variants). Here, we first divide the entire dataset of 174 subjects into 10 sets; we then use 9/10 of them to train the EMPaSchiz model (see Fig. 7b, c); we then run that model on the remaining 1/10 of the data (see Fig. 7a). We then compute accuracy as the number of correctly labelled instances, over all 10 folds, and use this as an estimate of the score of the learned EMPaSchiz -Performance system. We run this entire process five times—over five different partitionings and compute the overall accuracy of predictions over these 50 train-test splits. Trained models are depicted in green lines and predictions are depicted in red lines

EMPaSchiz produced a classifier from our multi-source data, in two levels. For the first level, EMPaSchiz trained 84 different L2-regularized logistic regression classifiers, using the ‘liblinear’ solver^80^ – one for each individual data source to predict the diagnosis; we consider each to be a single-source model (SSM). For the second level, EMPaSchiz then trained a single L2-regularized logistic regression model to take the prediction probabilities computed by each SSM, to predict the schizophrenia-vs-normal label; hence, this is a multi-source model (MSM). Figures 7 and 8 show schematic representations of our prediction and evaluation framework. These computations were performed using the scikit-learn package^36^ and mlxtend extensions.^81^

Figure 7a shows performance of learned EMPaSchiz-Performance model. Given a resting state fMRI time series for a subject, the EMPaSchiz-Performance first extracts 6 different feature types (F_1_ to F_6_; coded here with different fill colours) over each of 14 brain parcellation schemes (P_1_ to P_14_; coded here with border colour) to obtain 84 feature sets (FS_1,1_ to FS_6,14_). Each is given to a “single-source model” (SSM), which is a learned logistic regression (LR) classifier of the PCA-projection of that data with learned parameter *θ*_i,j_ (i.e, *θ*_1,1_ to *θ*_6,14_ each correspond to a specific feature set) trained to predict schizophrenia. This produces a vector of the resulting 84 prediction probability values (P_1,1_ to P_6,14_)—one from each LR—which is given to a final trained LR classifier with learned parameter θ_*,*_. The final prediction probability P_*,*_ is used to predict whether the given subject is “schizophrenia” or “normal”. We also considered 6 other multi-source models, with learned parameters *θ*_1,*_ to *θ*_6,*_—one for each feature type.

Figure 7b, c shows the process for learning the EMPaSchiz-Performance model. The EMPaSchiz-Learner first learns 84 different single-source models SSM_i,j_: For the *i*th feature type (*i* = 1..6) and the *j*th parcellation (*j* = 1..14), EMPaSchiz-Learner computes the (*i*,*j*)-feature set for the resting state fMRI time series for each of the *K* labelled subjects in training set, to obtain the feature sets FS^*^_i,*j*_ = { FS^*k*^_*i*,*j*_ } over *k* = 1..*K*. It then trains a regularized logistic regression (LR) model *θ*_*i*,*j*_ to predict schizophrenia, from each feature set FS ^*^_i,*j*_, where the regularization strength *C* is obtained using internal CV. For example, *θ*_3,12_ is learned by fitting LR on FS^*^_3,12_ (which corresponds to the 3rd feature type: ReHo with the 12th parcellation: destrieux). After learning all the 84 SSM parameters {*θ*_*i*,*j*_} in this manner, EMPaSchiz-Learner as shown in Fig. 7c, then runs each of these 84 resulting SSMs on each of the *K* training instances; this produces a new training set *P* = {*P* ^*k*^_*i*,*j*_ }, where *P* ^*k*^_*i*,*j*_ is the probability produced by running the (*i*,*j*)-th SSM predictor, with learned parameter *θ*_*i*,*j*_, on the *k*-th instance. It then learns the multi-source model (MSM) by training the regularized logistic regression (LR) on the set *P* to predict schizophrenia. This produces the parameter θ_*,*_. Similarly, six other MSMs *θ*_1,*_ to *θ*_6,*_ are learned by training LR with each set *P* ^*^_1,*j* = _{*P* ^*k*^_1,*j*_ } over *k* = 1..*K*, *j* = 1..14 to *P* ^*^_6,*j*_ = {*P* ^*k*^_6,*j*_ } over *k* = 1..*K*, *j* = 1..14.

In more detail: EMPaSchiz first used singular value decomposition of each data source to project it to a lower dimensional space. We extracted principal components from the training instances, then projected each instance onto the eigenvectors (PCA). We used all the components—i.e., set the number of principal components to the smaller of the number of original features or the number of instances. Note these components captured all the variance, but reduced the dimensionality by a huge factor, for most datasets, as the final number of features for each data source was at most the number of instances in training set (~157 subjects in our 10-fold cross-validation). For the few data sources that had fewer features than training instances (e.g., yeo-regional has 17 features), this transformation would not change the number of features, but changed the data to a new basis. The motivation for this procedure was to have a uniform pipeline of PCA transformations for all data sources, irrespective of the varying number of features.

For SSM, EMPaSchiz set the C parameter (inverse of regularization strength) by internal 10-fold cross-validation on the training split (5 shuffled iterations). We call the MSM that combined predictions from all 14 x 6 = 84 SSMs, ‘stacked-multi’. We also considered six other versions of MSM, each combining SSMs for a specific feature type (14 each): stacked-ALFF, stacked-fALFF, stacked-ReHo, stacked-FC-correlation, stacked-FC-partial correlation, stacked-FC-precision.

The EMPaSchiz model was evaluated in five shuffled iterations of a 10-fold balanced cross-validation approach (90% training set, 10% test set; for a total of 50 train-test splits). We evaluated the model’s generalization performance on the test set (in outer cross-validation), computing:

- accuracy (Overall, how often is the classifier correct?)
- sensitivity (When the actual label is ‘patient’, how often is the prediction correct?)
- specificity (When the actual label is ‘control’, how often is the prediction correct?)
- precision (When the predicted label is ‘patient’, how often is the prediction correct?)

For each variant, we report the mean and standard errors for these metrics over all 50 train-test splits. To compare MSM models, we used parametric statistical tests (two sided *t*-test) on the accuracy, using the SciPy package.^82^

We also performed two additional analyses. First, we explored the effect of feature selection with respect to SSM, using the top-*r* percentage of the total set of features, based on univariate testing (F-value score) on the model performance. For example, when *r* = 20%, the EMPaSchiz-Learner would use only 20% of the original features, in each of its 84 SSMs. Note this is instead of using PCA. (So, for the regional features of the ‘aal’ parcellation, instead of using all 116 features, it only considered the top 0.2 × 116 = 23 features, etc.) While computing the cross-validation scores, we ran the feature selection process ‘in fold’ using the ‘pipeline’ class of scikit-learn^58^ to avoid obtaining optimistically biased estimates. Second, we examined our ensemble prediction framework to distinguish the least symptomatic schizophrenia patients vs. the most symptomatic patients (based on SAPS and SANS); evaluated using leave-one out cross-validation.

## Supplementary information

Supplementary Materials

## Data Availability

The datasets generated during and/or analysed during the current study as well as relevant computer codes that were used to process the data and to generate the results are available from corresponding authors on a reasonable request.

## Supplementary information

**Supplementary information** accompanies the paper on the *npj Schizophrenia* website (10.1038/s41537-018-0070-8).

## References

[1] Thomas RI (2014). The NIMH Research Domain Criteria (RDoC) Project: precision medicine for psychiatry. Am. J. Psychiatry.

[2] Gejman PV, Sanders AR, Kendler KS (2011). Genetics of schizophrenia: new findings and challenges. Annu. Rev. Genom. Hum. Genet..

[3] Sass LA, Parnas J (2003). Schizophrenia, consciousness, and the self. Schizophr. Bull..

[4] Kapur S (2003). Psychosis as a state of aberrant salience: a framework linking biology, phenomenology, and pharmacology in schizophrenia. Am. J. Psychiatry.

[5] Cuthbert BN, Insel TR (2013). Toward the future of psychiatric diagnosis: the seven pillars of RDoC. BMC Med..

[6] Hyman SE (2007). Can neuroscience be integrated into the DSM-V?. Nat. Rev. Neurosci..

[7] Pieper AA, Baraban JM (2017). Moving beyond serendipity to mechanism-driven psychiatric therapeutics. Neurotherapeutics.

[8] Goldberg JF, Ernst CL (2016). Core concepts involving adverse psychotropic drug effects: assessment, implications, and management. Psychiatr. Clin. North Am..

[9] Kessler RC (2005). Prevalence and treatment of mental disorders, 1990 to 2003. N. Engl. J. Med..

[10] Hunger SP (2012). Improved survival for children and adolescents with acute lymphoblastic leukemia between 1990 and 2005: a report from the children’s oncology group. J. Clin. Oncol..

[11] National Heart, Lung, and Blood Institute. (2011). NHLBI Fact Book, Fiscal Year.

[12] Kahn RS (2015). Schizophrenia. Nat. Rev. Dis. Prim..

[13] Jablensky A (2010). The diagnostic concept of schizophrenia: its history, evolution, and future prospects. Dialog. Clin. Neurosci..

[14] Kendell R, Jablensky A (2003). Distinguishing between the validity and utility of psychiatric diagnoses. Am. J. Psychiatry.

[15] Huys QJM, Maia TV, Frank MJ (2016). Computational psychiatry as a bridge from neuroscience to clinical applications. Nat. Neurosci..

[16] Orrù G, Pettersson-Yeo W, Marquand AF, Sartori G, Mechelli A (2012). Using support vector machine to identify imaging biomarkers of neurological and psychiatric disease: a critical review. Neurosci. Biobehav. Rev..

[17] Lesh TA (2015). A multimodal analysis of antipsychotic effects on brain structure and function in first-episode schizophrenia. JAMA Psychiatry.

[18] Schnack HG, Kahn RS (2016). Detecting neuroimaging biomarkers for psychiatric disorders: sample size matters. Front. Psychiatry.

[19] Zilles K, Amunts K (2009). Receptor mapping: architecture of the human cerebral cortex. Curr. Opin. Neurol..

[20] Eickhoff SB, Rottschy C, Kujovic M, Palomero-Gallagher N, Zilles K (2008). Organizational principles of human visual cortex revealed by receptor mapping. Cereb. Cortex.

[21] Talairach, J. & Tournoux, P. *Co-Planar Stereotaxic Atlas of the Human Brain: 3-Dimensional Proportional System: An Approach to Cerebral Imaging* (G. Thieme, New York, 1988).

[22] Desikan RS (2006). An automated labeling system for subdividing the human cerebral cortex on MRI scans into gyral based regions of interest. Neuroimage.

[23] Damoiseaux JS, Greicius MD (2009). Greater than the sum of its parts: a review of studies combining structural connectivity and resting-state functional connectivity. Brain Struct. Funct..

[24] Roca P (2010). Inter-subject connectivity-based parcellation of a patch of cerebral cortex. Med Image Comput. Comput. Assist Interv..

[25] Yeo BT (2011). The organization of the human cerebral cortex estimated by intrinsic functional connectivity. J. Neurophysiol..

[26] Smith SM (2009). Correspondence of the brain’s functional architecture during activation and rest. Proc. Natl Acad. Sci. USA.

[27] Lashkari D (2012). Search for patterns of functional specificity in the brain: a nonparametric hierarchical Bayesian model for group fMRI data. Neuroimage.

[28] Dosenbach NU (2010). Prediction of individual brain maturity using fMRI. Science.

[29] Eickhoff SB (2011). Co-activation patterns distinguish cortical modules, their connectivity and functional differentiation. Neuroimage.

[30] Cordes D, Haughton V, Carew JD, Arfanakis K, Maravilla K (2002). Hierarchical clustering to measure connectivity in fMRI resting-state data. Magn. Reson. Imaging.

[31] McKeown MJ (1998). Analysis of fMRI data by blind separation into independent spatial components. Hum. Brain. Mapp..

[32] Yao Z, Hu B, Xie Y, Moore P, Zheng J (2015). A review of structural and functional brain networks: small world and atlas. Brain Inform..

[33] Thirion, B., Varoquaux, G., Dohmatob, E. & Poline, J.-B. Which fMRI clustering gives good brain parcellations? *Front. Neurosci.* **8**, 167 (2014). 10.3389/fnins.2014.00167 PMC4076743 25071425

[34] Cabral C (2016). Classifying schizophrenia using multimodal multivariate pattern recognition analysis: evaluating the impact of individual clinical profiles on the neurodiagnostic performance. Schizophr. Bull..

[35] Xia M, Wang J, He Y (2013). BrainNet viewer: a network visualization tool for human brain connectomics. PLoS ONE.

[36] Menon V, Uddin LQ (2010). Saliency, switching, attention and control: a network model of insula function. Brain. Struct. Funct..

[37] Takamura T, Hanakawa T (2017). Clinical utility of resting-state functional connectivity magnetic resonance imaging for mood and cognitive disorders. J. Neural Transm..

[38] van Amelsvoort T, Hernaus D (2016). Effect of pharmacological interventions on the fronto-cingulo-parietal cognitive control network in psychiatric disorders: a transdiagnostic systematic review of fMRI studies. Front. Psychiatry.

[39] Hu ML (2017). A review of the functional and anatomical default mode network in schizophrenia. Neurosci. Bull..

[40] Vega Romero, R., Brown, M. & Greiner, R. The challenge of applying machine learning techniques to diagnose schizophrenia using multi-site fMRI data. MSc Thesis, University of Alberta (2017).

[41] Alderson-Day B, McCarthy-Jones S, Fernyhough C (2015). Hearing voices in the resting brain: A review of intrinsic functional connectivity research on auditory verbal hallucinations. Neurosci. Biobehav. Rev..

[42] Curcic-Blake B (2017). Interaction of language, auditory and memory brain networks in auditory verbal hallucinations. Prog. Neurobiol..

[43] Alderson-Day B (2016). Auditory hallucinations and the brain’s resting-state networks: findings and methodological observations. Schizophr. Bull..

[44] Damaraju E (2014). Dynamic functional connectivity analysis reveals transient states of dysconnectivity in schizophrenia. NeuroImage.

[45] Yu Q (2012). Brain connectivity networks in schizophrenia underlying resting state functional magnetic resonance imaging. Curr. Top. Med. Chem..

[46] Zhou Y, Fan L, Qiu C, Jiang T (2015). Prefrontal cortex and the dysconnectivity hypothesis of schizophrenia. Neurosci. Bull..

[47] Pettersson-Yeo W, Allen P, Benetti S, McGuire P, Mechelli A (2011). Dysconnectivity in schizophrenia: where are we now?. Neurosci. Biobehav. Rev..

[48] Fornito A, Bullmore ET (2015). Reconciling abnormalities of brain network structure and function in schizophrenia. Curr. Opin. Neurobiol..

[49] Friston K, Brown HR, Siemerkus J, Stephan KE (2016). The dysconnection hypothesis. Schizophr. Res..

[50] Monte-Silva K (2013). Induction of late LTP-like plasticity in the human motor cortex by repeated non-invasive brain stimulation. Brain Stimul..

[51] Moseley P, Alderson-Day B, Ellison A, Jardri R, Fernyhough C (2015). Non-invasive brain stimulation and auditory verbal hallucinations: new techniques and future directions. Front. Neurosci..

[52] Bose A (2018). Efficacy of fronto-temporal transcranial direct current stimulation for refractory auditory verbal hallucinations in schizophrenia: a randomized, double-blind, sham-controlled study. Schizophr. Res..

[53] Mondino M (2016). Effects of fronto-temporal transcranial direct current stimulation on auditory verbal hallucinations and resting-state functional connectivity of the left temporo-parietal junction in patients with schizophrenia. Schizophr. Bull..

[54] Sheehan DV (1998). The Mini-International Neuropsychiatric Interview (M.I.N.I.): the development and validation of a structured diagnostic psychiatric interview for DSM-IV and ICD-10. J. Clin. Psychiatry.

[55] Andreasen NC, Arndt S, Miller D, Flaum M, Nopoulos P (1995). Correlational studies of the scale for the assessment of negative symptoms and the scale for the assessment of positive symptoms: an overview and update. Psychopathology.

[56] Chao-Gan Y, Yu-Feng Z (2010). DPARSF: a MATLAB toolbox for “Pipeline” data analysis of resting-state fMRI. Front. Syst. Neurosci..

[57] Abraham A (2014). Machine learning for neuroimaging with scikit-learn. Front. Neuroinform..

[58] Pedregosa F (2011). Scikit-learn: machine learning in Python. J. Mach. Learn. Res..

[59] Power JD, Barnes KA, Snyder AZ, Schlaggar BL, Petersen SE (2012). Spurious but systematic correlations in functional connectivity MRI networks arise from subject motion. Neuroimage.

[60] Satterthwaite TD (2012). Impact of in-scanner head motion on multiple measures of functional connectivity: relevance for studies of neurodevelopment in youth. Neuroimage.

[61] Van Dijk KR, Sabuncu MR, Buckner RL (2012). The influence of head motion on intrinsic functional connectivity MRI. Neuroimage.

[62] Chang X (2015). Distinct inter-hemispheric dysconnectivity in schizophrenia patients with and without auditory verbal hallucinations. Sci. Rep..

[63] Friston KJ (1994). Statistical parametric maps in functional imaging: a general linear approach. Hum. Brain Mapp..

[64] Varoquaux G, Gramfort A, Pedregosa F, Michel V, Thirion B (2011). Multi-subject dictionary learning to segment an atlas of brain spontaneous activity. Inf. Process. Med. Imaging.

[65] Tzourio-Mazoyer N (2002). Automated anatomical labeling of activations in SPM using a macroscopic anatomical parcellation of the MNI MRI single-subject brain. Neuroimage.

[66] Bellec P, Rosa-Neto P, Lyttelton OC, Benali H, Evans AC (2010). Multi-level bootstrap analysis of stable clusters in resting-state fMRI. Neuroimage.

[67] Destrieux C, Fischl B, Dale A, Halgren E (2009). A sulcal depth-based anatomical parcellation of the cerebral cortex. Neuroimage.

[68] Power JD (2011). Functional network organization of the human brain. Neuron.

[69] Zou QH (2008). An improved approach to detection of amplitude of low-frequency fluctuation (ALFF) for resting-state fMRI: fractional ALFF. J. Neurosci. Methods.

[70] Hoptman MJ (2010). Amplitude of low-frequency oscillations in schizophrenia: a resting state fMRI study. Schizophr. Res..

[71] Zang YF (2007). Altered baseline brain activity in children with ADHD revealed by resting-state functional MRI. Brain Dev..

[72] Kendall MG (1948). Rank Correlation Methods.

[73] Zang Y, Jiang T, Lu Y, He Y, Tian L (2004). Regional homogeneity approach to fMRI data analysis. Neuroimage.

[74] Crowley S (2018). Considering total intracranial volume and other nuisance variables in brain voxel based morphometry in idiopathic PD. Brain Imaging Behav..

[75] Turner J (2013). A multi-site resting state fMRI study on the amplitude of low frequency fluctuations in schizophrenia. Front. Neurosci..

[76] Liu H (2006). Decreased regional homogeneity in schizophrenia: a resting state functional magnetic resonance imaging study. Neuroreport.

[77] Chen J (2013). Comparative study of regional homogeneity in schizophrenia and major depressive disorder. Am. J. Med. Genet..

[78] Lynall ME (2010). Functional connectivity and brain networks in schizophrenia. J. Neurosci..

[79] Das A (2017). Interpretation of the precision matrix and its application in estimating sparse brain connectivity during sleep spindles from human electrocorticography recordings. Neural Comput..

[80] Fan RE, Chang KW, Hsieh CJ, Wang XR, Lin CJ (2008). LIBLINEAR: A library for large linear classification. J. Mach. Learn. Res..

[81] Raschka, S. MLxtend: Providing machine learning and data science utilities and extensions to Python’s scientific computing stack. *J. Open Source Softw.* 3, 24 10.21105/joss.00638 (2018). http://joss.theoj.org/papers/10.21105/joss.00638.

[82] Jones, E., Oliphant, T. & Peterson, P. SciPy: Open source scientific tools for Python (2001). http://www.scipy.org/.

[83] Shen H, Wang L, Liu Y, Hu D (2010). Discriminative analysis of resting-state functional connectivity patterns of schizophrenia using low dimensional embedding of fMRI. Neuroimage.

[84] Fan Y (2011). Discriminant analysis of functional connectivity patterns on Grassmann manifold. Neuroimage.

[85] Yu Y, Shen H, Zeng LL, Ma Q, Hu D (2013). Convergent and divergent functional connectivity patterns in schizophrenia and depression. PLoS ONE.

[86] Anderson A, Cohen MS (2013). Decreased small-world functional network connectivity and clustering across resting state networks in schizophrenia: an fMRI classification tutorial. Front. Hum. Neurosci..

[87] Arbabshirani M, Kiehl K, Pearlson G, Calhoun V (2013). Classification of schizophrenia patients based on resting-state functional network connectivity. Front. Neurosci..

[88] Yu Y (2013). Functional connectivity-based signatures of schizophrenia revealed by multiclass pattern analysis of resting-state fMRI from schizophrenic patients and their healthy siblings. Biomed. Eng. Online.

[89] Guo S, Kendrick KM, Yu R, Wang HLS, Feng J (2014). Key functional circuitry altered in schizophrenia involves parietal regions associated with sense of self. Hum. Brain. Mapp..

[90] Brodersen KH (2014). Dissecting psychiatric spectrum disorders by generative embedding. NeuroImage. Clin..

[91] Anticevic A (2014). Characterizing thalamo-cortical disturbances in schizophrenia and bipolar illness. Cereb. Cortex.

[92] Watanabe T, Kessler D, Scott C, Angstadt M, Sripada C (2014). Disease prediction based on functional connectomes using a scalable and spatially-informed support vector machine. Neuroimage.

[93] Chyzhyk D, Grana M, Ongur D, Shinn AK (2015). Discrimination of schizophrenia auditory hallucinators by machine learning of resting-state functional MRI. Int. J. Neural Syst..

[94] Cheng W (2015). Voxel-based, brain-wide association study of aberrant functional connectivity in schizophrenia implicates thalamocortical circuitry. NPJ Schizophr..

[95] Peters H (2016). More consistently altered connectivity patterns for cerebellum and medial temporal lobes than for amygdala and striatum in schizophrenia. Front. Hum. Neurosci..

[96] Mikolas P (2016). Connectivity of the anterior insula differentiates participants with first-episode schizophrenia spectrum disorders from controls: a machine-learning study. Psychol. Med..

[97] Yang H, He H, Zhong J (2016). Multimodal MRI characterisation of schizophrenia: a discriminative analysis. Lancet.

[98] Iwabuchi SJ, Palaniyappan L (2017). Abnormalities in the effective connectivity of visuothalamic circuitry in schizophrenia. Psychol. Med..

[99] Lottman KK (2017). Risperidone effects on brain dynamic connectivity—a prospective resting-state fMRI study in schizophrenia. Front. Psychiatry.

[100] Guo W (2017). Family-based case-control study of homotopic connectivity in first-episode, drug-naive schizophrenia at rest. Sci. Rep..

