# Supplementary material for: Towards artificial intelligence in mental health by improving schizophrenia prediction with multiple brain parcellation ensemble-learning
Source: NPJ Schizophr. 2019 Jan 18;5:2. doi: 10.1038/s41537-018-0070-8 (PMC6386753; doi:10.1038/s41537-018-0070-8)
Supplement: Supplementary file 1 — Supplementary Materials [file 41537_2018_70_MOESM1_ESM.pdf]

**Supplementary Table 1: Description of studies that provided machine learning model for predicting schizophrenia using resting-state brain patterns (SCZ: Schizophrenia, HC: Healthy controls; AH: Auditory Hallucinations, FE: First episode, MDD: Major Depression, CV: Cross-validation, LOO : leave one out)**

| Study | Year | Sample size | Multi/Single Site | Data Acquisition | Feature selection/reduction | Classifier | CV | Accuracy | Reference |
|---|---|---|---|---|---|---|---|---|---|
| **Shen** | 2010 | 32 SCZ, 20 HC | single-site (Second Xiangya Hospital of Central South University in Changsha, China) | Resting state | Kendall tau correlation - low-dimensional embedding | C-Means clustering | LOO | 86.50% | Shen, H., Wang, L., Liu, Y., & Hu, D. (2010). Discriminative analysis of resting-state functional connectivity patterns of schizophrenia using low dimensional embedding of fMRI. NeuroImage, 49(4), 3110–3121. https://doi.org/10.1016/j.neuroimage.2009.11.011 |
| **Fan** | 2011 | 31 SCZ, 31 HC | Single-site ( Institute of Mental Health, Second Xiangya Hospital, China) | Resting state | ICA + Grassmann manifold | SVM - various kernels | LOO | upto 87.1% | Fan, Y., Liu, Y., Wu, H., Hao, Y., Liu, H., Liu, Z., & Jiang, T. (2011). Discriminant analysis of functional connectivity patterns on Grassmann manifold. NeuroImage, 56(4), 2058–2067. https://doi.org/10.1016/j.neuroimage.2011.03.051 |
| **Anderson** | 2013 | 74 SCZ, 72 HC | single-site (COBRE dataset -multiple studies from University of New Mexico) | Resting state | ICA, graph-theoretic connectivity measures such as graph density, average path length, and small-worldness. | SVM | 10-fold | 65% | Anderson A, & Cohen MS. (2013). Decreased small-world functional network connectivity and clustering across resting state networks in schizophrenia: an fMRI classification tutorial. Frontiers in human neuroscience, 7, 520-520. doi: 10.3389/fnhum.2013.00520 |
| **Arbabshirani** | 2013 | 28 SCZ, 28 HC | single-site (Hartford hospital and Yale) | Resting state | ICA | Multiple Learners | LOO | upto 96% | Arbabshirani, M., Kiehl, K., Pearlson, G., & Calhoun, V. (2013). Classification of schizophrenia patients based on resting-state functional network connectivity . *Frontiers in Neuroscience* . Retrieved from http://journal.frontiersin.org/article/10.3389/fnins.2013.00133 |
| **Yu** | 2013 | 24 SCZ, 25 healthy siblings of SCZ, 22 HC | single-site (Second Xiangya Hospital of Central South University) | Resting state | PCA | SVM | LOO | 62.00% | Yu, Y., Shen, H., Zhang, H., Zeng, L.-L., Xue, Z., & Hu, D. (2013). Functional connectivity-based signatures of schizophrenia revealed by multiclass pattern analysis of resting-state fMRI from schizophrenic patients and their healthy siblings. *Biomedical Engineering Online*, *12*, 10. https://doi.org/10.1186/1475-925X-12-10 |
| **Yu** | 2013 | 32 SCZ, 38 HC (+19 MDD) | Single-site (Second Xiangya Hospital of Central South University) | Resting state | intrinsic discriminant analysis | SVM | LOO | 80.9% | Yu, Y., Shen, H., Zeng, L.-L., Ma, Q., & Hu, D. (2013). Convergent and divergent functional connectivity patterns in schizophrenia and depression. PloS One, 8(7), e68250. https://doi.org/10.1371/journal.pone.0068250 |
| **Anticevic** | 2014 | 90 SCZ, 90 HC | single-site (outpatient clinics and community mental health facilities in the Hartford area) | Resting state | MVPA | linear SVM | LOO | 73.9% | Anticevic, A., Cole, M. W., Repovs, G., Murray, J. D., Brumbaugh, M. S., Winkler, A. M., … Glahn, D. C. (2014). Characterizing thalamo-cortical disturbances in schizophrenia and bipolar illness. Cerebral Cortex (New York, N.Y. : 1991), 24(12), 3116–3130. https://doi.org/10.1093/cercor/bht165 |

| | | | | | | | | | |
|---|---|---|---|---|---|---|---|---|---|
| **Watanabe** | 2014 | 54 SCZ, 67 HC | single-site (COBRE dataset -multiple studies from University of New Mexico) | Resting state | Fused Lasso or the GraphNet regularizer. | SVM | 10-fold | 73.50% | Watanabe, T., Kessler, D., Scott, C., Angstadt, M., & Sripada, C. (2014). Disease prediction based on functional connectomes using a scalable and spatially-informed support vector machine. NeuroImage, 96, 183–202. https://doi.org/10.1016/j.neuroimage.2014.03.067 |
| **Guo S** | 2014 | 69 SCZ, 62 HC | Single-site (National Taiwan University Hospital) | Resting state | FC link effect | SVM | LOO | 80% | Guo, S., Kendrick, K. M., Yu, R., Wang, H.-L. S., & Feng, J. (2014). Key functional circuitry altered in schizophrenia involves parietal regions associated with sense of self. Human Brain Mapping, 35(1), 123–139. https://doi.org/10.1002/hbm.22162 |
| **Brodersen** | 2014 | 41 SCZ, 42 HC | Single-site (Department of Psychiatry and Psychotherapy (Campus Charité Mitte) of the Charité Universitätsmedizin) | n-back working-memory task | dynamic causal model (DCM), FC & regional activity | linear SVM, variational Bayesian Gaussian mixture model | 5 fold CV | upto 78% | Brodersen, K. H., Deserno, L., Schlagenhauf, F., Lin, Z., Penny, W. D., Buhmann, J. M., & Stephan, K. E. (2014). Dissecting psychiatric spectrum disorders by generative embedding. NeuroImage. Clinical, 4, 98–111. https://doi.org/10.1016/j.nicl.2013.11.002 |
| **Arbabshirani** | 2014 | 195 SCZ, 175 HC | multi-site (7 sites) | Resting state | GICA + FC / Autoconnectivity (AR1) + minimum redundancy maximum relevancy | SVM (various kernels) | 10 fold CV ?? (not clear) | 85% ? (table says 88.2%) | Arbabshirani, M. R., Castro, E., & Calhoun, V. D. (2014). Accurate classification of schizophrenia patients based on novel resting-state fMRI features. Conference Proceedings : ... Annual International Conference of the IEEE Engineering in Medicine and Biology Society. IEEE Engineering in Medicine and Biology Society. Annual Conference, 2014, 6691–6694. https://doi.org/10.1109/EMBC.2014.6945163 |
| **Chyzhyk** | 2015 | 26 SCZ with history of AH, 14 SCZ without a history of AH, 28 HC | | Resting state | Heschl's gyrus seed based FC computed by lattice auto-associative memories | SVM | 10-fold | upto 97.1% | Chyzhyk, D., Grana, M., Ongur, D., & Shinn, A. K. (2015). Discrimination of schizophrenia auditory hallucinators by machine learning of resting-state functional MRI. *International Journal of Neural Systems*, *25*(3), 1550007. https://doi.org/10.1142/S0129065715500070 |
| **Cheng** | 2015 | 19 SCZ, 29 HC | single-site (Indiana University) | Resting state | Betweenness centrality | Linear SVM | LOO | 79% | Cheng, H., Newman, S., Goni, J., Kent, J. S., Howell, J., Bolbecker, A., … Hetrick, W. P. (2015). Nodal centrality of functional network in the differentiation of schizophrenia. *Schizophrenia Research*, *168*(1–2), 345–352. https://doi.org/10.1016/j.schres.2015.08.011 |
| **Cheng** | 2015 | 415 SCZ, 405 HC | multi-site (UK, USA, Taiwan, and China) | Resting state | region and voxel-based brain-wide association | SVM | LOO | 75.81 (overall) | Cheng, W., Palaniyappan, L., Li, M., Kendrick, K. M., Zhang, J., Luo, Q., … Feng, J. (2015). Voxel-based, brain-wide association study of aberrant functional connectivity in schizophrenia implicates thalamocortical circuitry. NPJ Schizophrenia, 1, 15016. https://doi.org/10.1038/npjschz.2015.16 |

| | | | | | | | | | |
|---|---|---|---|---|---|---|---|---|---|
| **Peters** | 2016 | 18 SCZ, 18 HC | single-site (Department of Psychiatry, Klinikum Rechts der Isar, TU München) | Resting state | Network and ROI FC | SVM | LOO | 91% with allROIs-NWs | Peters, H., Shao, J., Scherr, M., Schwerthoffer, D., Zimmer, C., Forstl, H., … Sorg, C. (2016). More Consistently Altered Connectivity Patterns for Cerebellum and Medial Temporal Lobes than for Amygdala and Striatum in Schizophrenia. Frontiers in Human Neuroscience, 10, 55. https://doi.org/10.3389/fnhum.2016.00055 |
| **Mikolas** | 2016 | 63 SCZ with FES, 63 HC | single-site (Bohnice Psychiatric Hospital, Prague) | Resting state | seed-based connectivity | Linear SVM | LOO | 73.00% | Mikolas, P., Melicher, T., Skoch, A., Matejka, M., Slovakova, A., Bakstein, E., … Spaniel, F. (2016). Connectivity of the anterior insula differentiates participants with first-episode schizophrenia spectrum disorders from controls: a machine-learning study. Psychological Medicine, 46(13), 2695–2704. https://doi.org/10.1017/S0033291716000878 |
| **Cabral** | 2016 | 66 SCZ, 66 HC | single-site (COBRE dataset -multiple studies from University of New Mexico) | Resting state (+ Structural MRI) | mutual Information + PCA | L2-regularized Logistic Regression | 20 x 20 repeated double CV framework | 70.5% (75% when combined with structural) | Cabral, C., Kambeitz-Ilankovic, L., Kambeitz, J., Calhoun, V. D., Dwyer, D. B., von Saldern, S., … Koutsouleris, N. (2016). Classifying Schizophrenia Using Multimodal Multivariate Pattern Recognition Analysis: Evaluating the Impact of Individual Clinical Profiles on the Neurodiagnostic Performance. *Schizophrenia Bulletin*, *42 Suppl 1*, S110-7. https://doi.org/10.1093/schbul/sbw053 |
| **Skåtun** | 2016 | 182 SCZ spectrum, 348 HC | multi-site (1 from the University of Oslo, Norway and 2 from Karolinska Institutet, Stockholm, Sweden) | Resting state | ICA | regularized linear discriminant analysis | | upto 78.3% | Skatun, K. C., Kaufmann, T., Doan, N. T., Alnaes, D., Cordova-Palomera, A., Jonsson, E. G., … Westlye, L. T. (2016). Consistent Functional Connectivity Alterations in Schizophrenia Spectrum Disorder: A Multisite Study. Schizophrenia Bulletin. https://doi.org/10.1093/schbul/sbw145 |
| **Yang** | 2016 | 40 SCZ, 46 HC | single-site (University of New Mexico, USA.) | Resting state(+ Structural MRI) | GICA | SVM, Maximum-uncertainty linear discriminant analysis | | 77·91% (combined) | Yang, H., He, H., & Zhong, J. (2016). Multimodal MRI characterisation of schizophrenia: a discriminative analysis. *Lancet (London, England)*, *388 Suppl*, S36. https://doi.org/10.1016/S0140-6736(16)31963-8 |
| **Iwabuchi** | 2017 | 62 SCZ, 71 HC | single-site (COBRE dataset -multiple studies from University of New Mexico) | Resting state | seed-based Granger causality analysis | SVM-multiple kernel learning classifier | LOO | 78.04% (ReHO) - combined was lesser | Iwabuchi, S. J., & Palaniyappan, L. (2017). Abnormalities in the effective connectivity of visuothalamic circuitry in schizophrenia. Psychological Medicine, 1–11. https://doi.org/10.1017/S0033291716003469 |
| **Lottman** | 2017 | 34 unmedicated (17 drug-naïve) SCZ + follow-up post-treatment, 35 HC | single-site (University of Alabama at Birmingham) | Resting state | GICA -Static & Dynamic FC | linear SVM | LOO | 83.8% (combined static & Dynamic FC) - static only: | Lottman, K. K., Kraguljac, N. V, White, D. M., Morgan, C. J., Calhoun, V. D., Butt, A., & Lahti, A. C. (2017). Risperidone Effects on Brain Dynamic Connectivity-A Prospective Resting-State fMRI Study in Schizophrenia. Frontiers in Psychiatry, 8, 14. https://doi.org/10.3389/fpsyt.2017.00014 |

| | | | | | | | | | |
|---|---|---|---|---|---|---|---|---|---|
| | | | | | | | | 73.5% | |
| **Guo W** | 2017 | 28 FE drug-naive SCZ, 28 family-based controls, 40 HC | single-site (Mental Health Center of the First Affiliated Hospital, Guangxi Medical University in China) | Resting state | Voxel-mirrored homotopic connectivity | rbf SVM | LOO | 92.86% (overfitting ??) | Guo, W., Liu, F., Chen, J., Wu, R., Li, L., Zhang, Z., & Zhao, J. (2017). Family-based case-control study of homotopic connectivity in first-episode, drug-naive schizophrenia at rest. *Scientific Reports*, *7*, 43312. https://doi.org/10.1038/srep43312 |

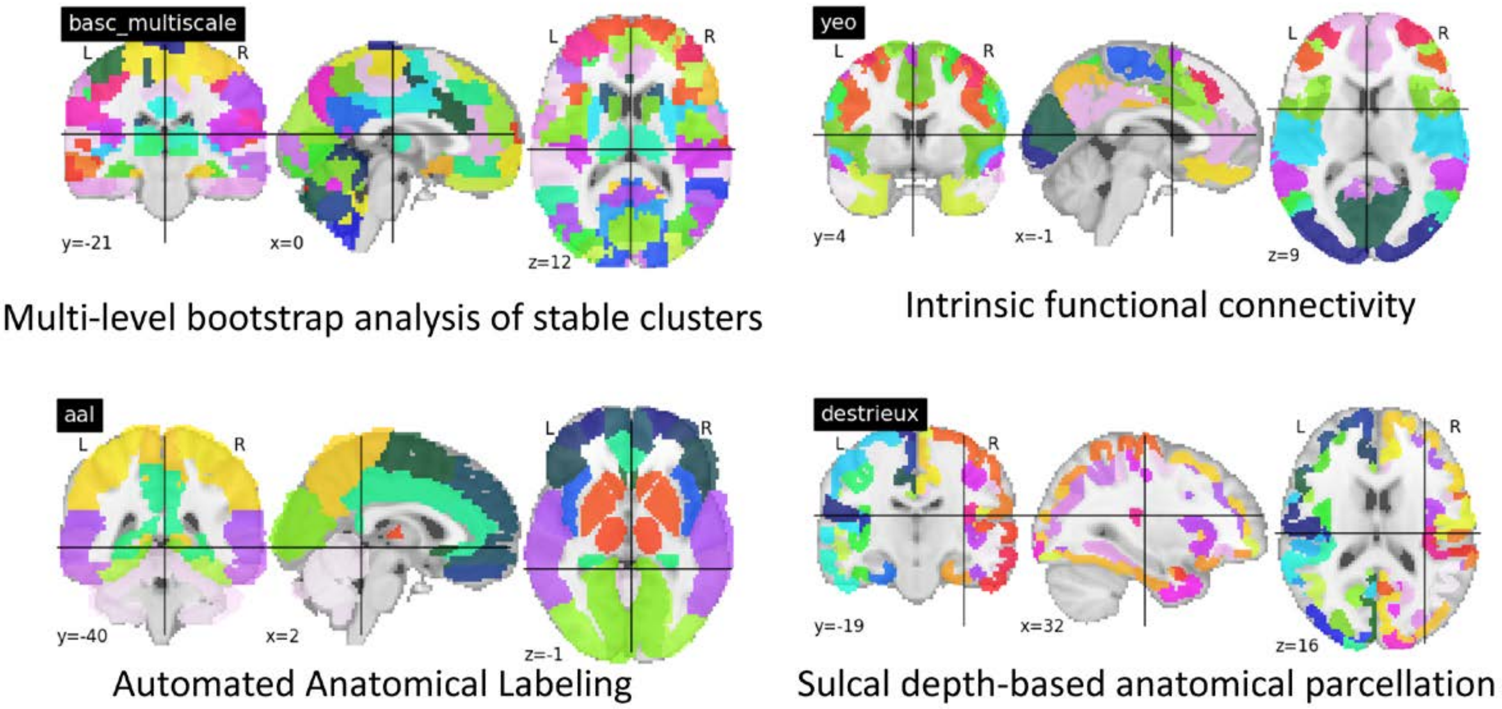


**Supplementary Figure 1 : MRI overlay of representative atlases that were used for parcellation in the study.**

**The coordinate mapping information used to these create figures comes from public data made available by the studies cited in the manuscript. Here, the basic boundaries of 3D areas from the schematic atlases is redrawn on one of our study subjects.**

**Supplementary Table 2. Demographic and clinical profile of study subjects**

| Characteristic | Schizophrenia | Control | Stat | p |
|---|---|---|---|---|
| **N** | 81 | 93 | | |
| **Sex [M:F]** | 53:28 | 60:33 | 0.001 ¶ | 0.97 |
| **Age** | 30.72 ± 6.16 | 29.41 ± 5.71 | 1.45 $ | 0.15 |
| **Total Intracranial Volume (TICV, mL)** | 1400 ± 135 | 1480 ± 144 | 3.76 $ | <0.001 |
| **Age at onset** | 26.6 ± 6.17 | - | - | - |
| **Duration of untreated illness (months)** | 36.95 ± 51.08 | - | - | - |
| **Total positive symptoms (SAPS)** | 27.65 ± 12.22 | - | - | - |
| **Total negative symptoms (SANS)** | 32.11 ± 27.79 | - | - | - |

**$** Independent Samples Test [t]

¶ Chi-Square test [$\chi^2$]

Men had significantly greater TICV than women (Stat$ = 6.7, p < 0.001), and age showed significant negative correlation with TICV (pearson r = -0.24, p = 0.001)

**Supplementary Table 3. Performance accuracy in percentage of single-source and stacked models for various feature-type and parcellations**

| | FC_corr | | FC_part | | FC_prec | | ALFF | | ReHo | | fALFF | |
|---|---|---|---|---|---|---|---|---|---|---|---|---|
| | Mean | Stdev | Mean | Stdev | Mean | Stdev | Mean | Stdev | Mean | Stdev | Mean | Stdev |
| **harvard_sub_25** | 0.67 | 0.12 | 0.55 | 0.12 | 0.52 | 0.10 | 0.67 | 0.10 | 0.69 | 0.10 | 0.61 | 0.11 |
| **smith20** | 0.68 | 0.12 | 0.63 | 0.11 | 0.65 | 0.10 | 0.64 | 0.12 | 0.67 | 0.12 | 0.67 | 0.12 |
| **msdl** | 0.72 | 0.12 | 0.59 | 0.11 | 0.59 | 0.11 | 0.67 | 0.09 | 0.71 | 0.10 | 0.70 | 0.11 |
| **harvard_cort_25** | 0.67 | 0.12 | 0.67 | 0.10 | 0.70 | 0.11 | 0.70 | 0.11 | 0.70 | 0.12 | 0.73 | 0.12 |
| **yeo** | 0.67 | 0.11 | 0.71 | 0.10 | 0.71 | 0.09 | 0.69 | 0.11 | 0.71 | 0.10 | 0.72 | 0.10 |
| **smith70** | 0.79 | 0.11 | 0.73 | 0.12 | 0.76 | 0.09 | 0.69 | 0.11 | 0.73 | 0.10 | 0.70 | 0.11 |
| **aal** | 0.71 | 0.09 | 0.75 | 0.11 | 0.78 | 0.12 | 0.72 | 0.11 | 0.72 | 0.11 | 0.73 | 0.10 |
| **destrieux** | 0.73 | 0.11 | 0.74 | 0.09 | 0.75 | 0.09 | 0.74 | 0.10 | 0.75 | 0.11 | 0.72 | 0.11 |
| **dosenbach** | 0.75 | 0.09 | 0.74 | 0.10 | 0.75 | 0.10 | 0.73 | 0.10 | 0.74 | 0.11 | 0.75 | 0.11 |
| **basc_multiscale_122** | 0.76 | 0.09 | 0.78 | 0.09 | 0.78 | 0.09 | 0.74 | 0.10 | 0.73 | 0.11 | 0.75 | 0.10 |
| **power** | 0.75 | 0.11 | 0.75 | 0.08 | 0.77 | 0.10 | 0.79 | 0.10 | 0.76 | 0.10 | 0.75 | 0.11 |
| **basc_multiscale_197** | 0.82 | 0.11 | 0.80 | 0.08 | 0.82 | 0.09 | 0.77 | 0.10 | 0.76 | 0.11 | 0.72 | 0.11 |
| **basc_multiscale_325** | 0.81 | 0.10 | 0.79 | 0.09 | 0.81 | 0.09 | 0.78 | 0.09 | 0.76 | 0.10 | 0.75 | 0.11 |
| **basc_multiscale_444** | 0.81 | 0.09 | 0.77 | 0.08 | 0.83 | 0.10 | 0.77 | 0.10 | 0.77 | 0.11 | 0.78 | 0.08 |
| **stack_*** | 0.82 | 0.09 | 0.79 | 0.10 | 0.84 | 0.08 | 0.76 | 0.10 | 0.74 | 0.11 | 0.75 | 0.10 |

**Supplementary Table 4. Model performance (in percentage) of the various parcellation-wise stacked learners: average (standard errors). All results are 10-fold CV.**

| | Accuracy | Precision | Sensitivity | Specificity |
|---|---|---|---|---|
| **stacked-multi** | 86.9 (1.1) | 91.9 (1.4) | 79.8 (1.8) | 93.1 (1.2) |
| | | | | |
| **stacked-aal** | 81.0 (1.4) | 83.1 (1.9) | 76.1 (2.1) | 85.3 (1.6) |
| **stacked-basc_multiscale_122** | 82.4 (1.3) | 86.0 (1.8) | 76.1 (1.6) | 87.9 (1.7) |
| **stacked-basc_multiscale_197** | 85.9 (1.0) | 89.9 (1.3) | 79.9 (1.8) | 91.1 (1.3) |
| **stacked-basc_multiscale_325** | 85.3 (1.1) | 89.6 (1.4) | 78.2 (1.8) | 91.5 (1.2) |
| **stacked-basc_multiscale_444** | 85.8 (1.3) | 89.8 (1.6) | 80.2 (2.3) | 90.7 (1.6) |
| **stacked-destrieux** | 81.2 (1.3) | 86.2 (1.8) | 72.7 (2.2) | 88.6 (1.6) |
| **stacked-dosenbach** | 82.8 (1.4) | 87.3 (1.7) | 76.1 (2.2) | 88.6 (1.8) |
| **stacked-harvard_cort_25** | 71.4 (1.7) | 74.7 (2.3) | 61.9 (2.7) | 79.7 (2.2) |
| **stacked-harvard_sub_25** | 65.1 (1.8) | 64.5 (2.4) | 58.8 (2.7) | 70.5 (2.2) |
| **stacked-msdl** | 69.5 (1.6) | 69.7 (1.9) | 62.1 (2.5) | 75.9 (1.9) |
| **stacked-power** | 81.1 (1.5) | 82.3 (1.9) | 77.8 (2.3) | 84.0 (1.9) |
| **stacked-smith20** | 69.9 (1.4) | 71.8 (2.2) | 62.5 (2.1) | 76.4 (2.0) |
| **stacked-smith70** | 78.9 (1.2) | 83.8 (1.8) | 70.8 (2.2) | 85.9 (1.8) |
| **stacked-yeo** | 72.8 (1.3) | 71.8 (1.5) | 69.9 (2.2) | 75.3 (1.6) |

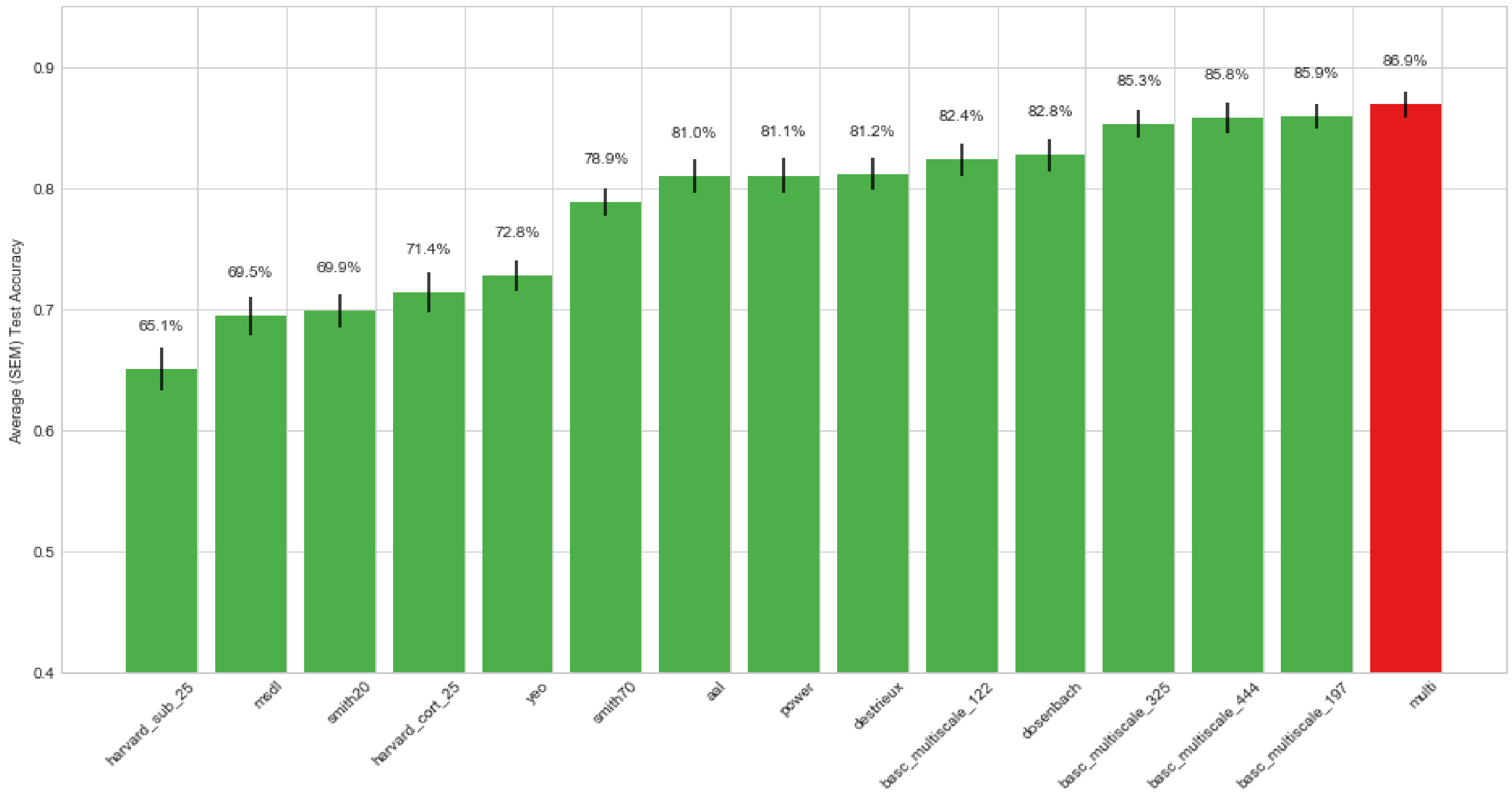


**Supplementary Figure 2. Comparison of 10-fold cross-validation prediction accuracies for parcellation-wise stacked learners.**

**Supplementary Table 5. Performance in percentage of multi-source ensemble models with various feature selection (FS) / reduction criteria (Average and standard errors)**

| | Accuracy | Precision | Sensitivity | Specificity |
|---|---|---|---|---|
| **PCA** | **0.87 (0.01)** | **0.92 (0.01)** | **0.8 (0.02)** | **0.93 (0.01)** |
| | | | | |
| **Top 0.5 % FS** | 0.82 (0.01) | 0.92 (0.01) | 0.67 (0.02) | 0.95 (0.01) |
| **Top 1 % FS** | 0.82 (0.01) | 0.92 (0.01) | 0.69 (0.02) | 0.94 (0.01) |
| **Top 2 % FS** | 0.83 (0.01) | 0.93 (0.01) | 0.69 (0.02) | 0.95 (0.01) |
| **Top 5 % FS** | 0.83 (0.01) | 0.93 (0.01) | 0.69 (0.02) | 0.95 (0.01) |
| **Top 10 % FS** | 0.83 (0.01) | 0.93 (0.01) | 0.69 (0.02) | 0.95 (0.01) |
| **Top 20 % FS** | 0.82 (0.01) | 0.91 (0.01) | 0.7 (0.02) | 0.94 (0.01) |
| **Top 30 % FS** | 0.83 (0.01) | 0.93 (0.02) | 0.69 (0.02) | 0.95 (0.01) |
| **Top 30 % FC, 100 % Regional FS** | 0.84 (0.01) | 0.92 (0.01) | 0.72 (0.02) | 0.94 (0.01) |
| **All 100 % FS (No FS)** | 0.85 (0.01) | 0.93 (0.01) | 0.73 (0.02) | 0.95 (0.01) |

Comparison of prediction accuracy for stacked models with various feature selection/reduction methods.
Final ensemble model shown in red.

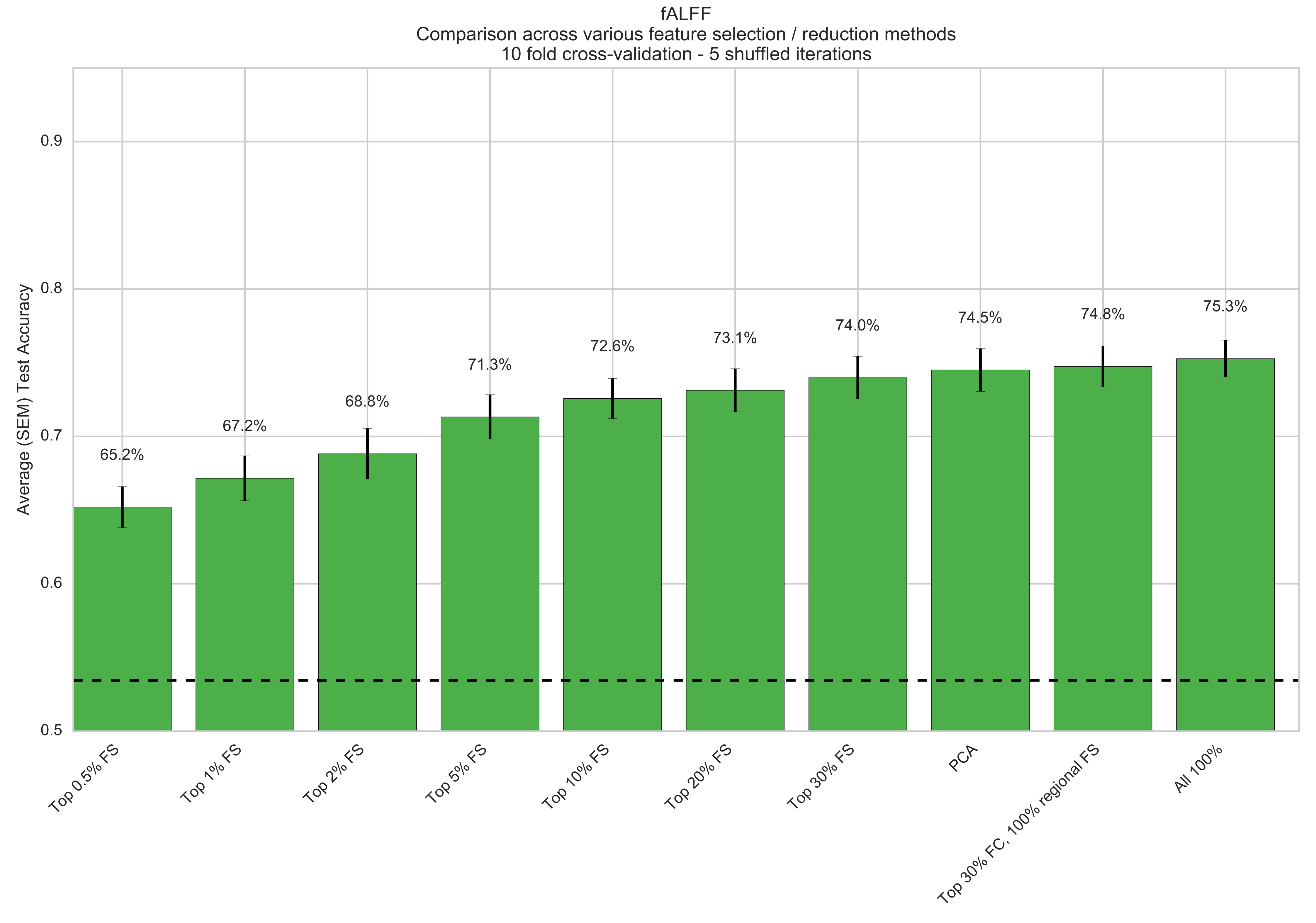
fALFF
Comparison across various feature selection / reduction methods
10 fold cross-validation - 5 shuffled iterations
Average (SEM) Test Accuracy
0.5
0.6
0.7
0.8
0.9
65.2%
67.2%
68.8%
71.3%
72.6%
73.1%
74.0%
74.5%
74.8%
75.3%
Top 0.5% FS
Top 1% FS
Top 2% FS
Top 5% FS
Top 10% FS
Top 20% FS
Top 30% FS
PCA
Top 30% FC, 100% regional FS
All 100%

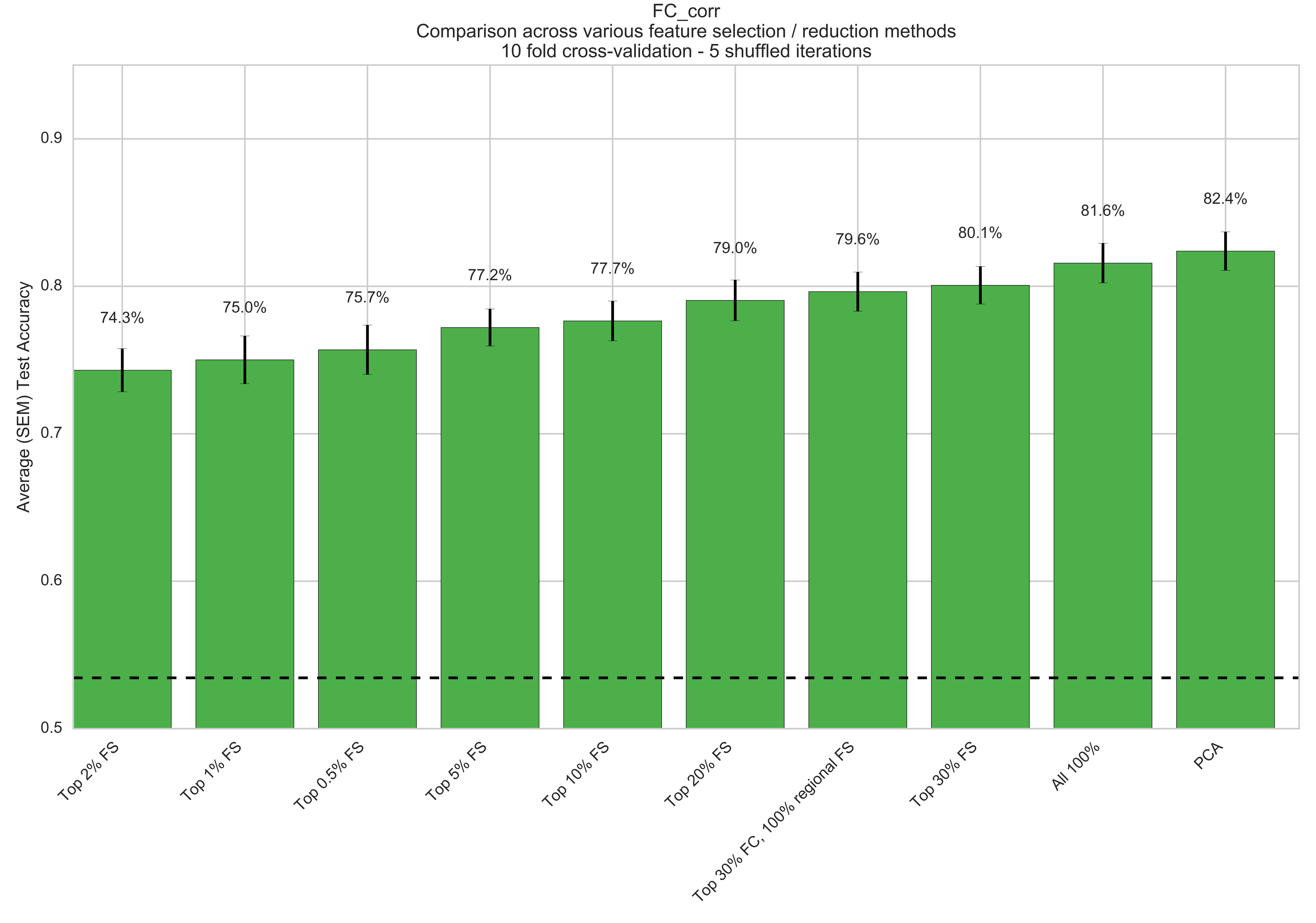
FC_corr
Comparison across various feature selection / reduction methods
10 fold cross-validation - 5 shuffled iterations
Average (SEM) Test Accuracy
0.9
0.8
0.7
0.6
0.5
74.3%
75.0%
75.7%
77.2%
77.7%
79.0%
79.6%
80.1%
81.6%
82.4%
Top 2% FS
Top 1% FS
Top 0.5% FS
Top 5% FS
Top 10% FS
Top 20% FS
Top 30% FC, 100% regional FS
Top 30% FS
All 100%
PCA

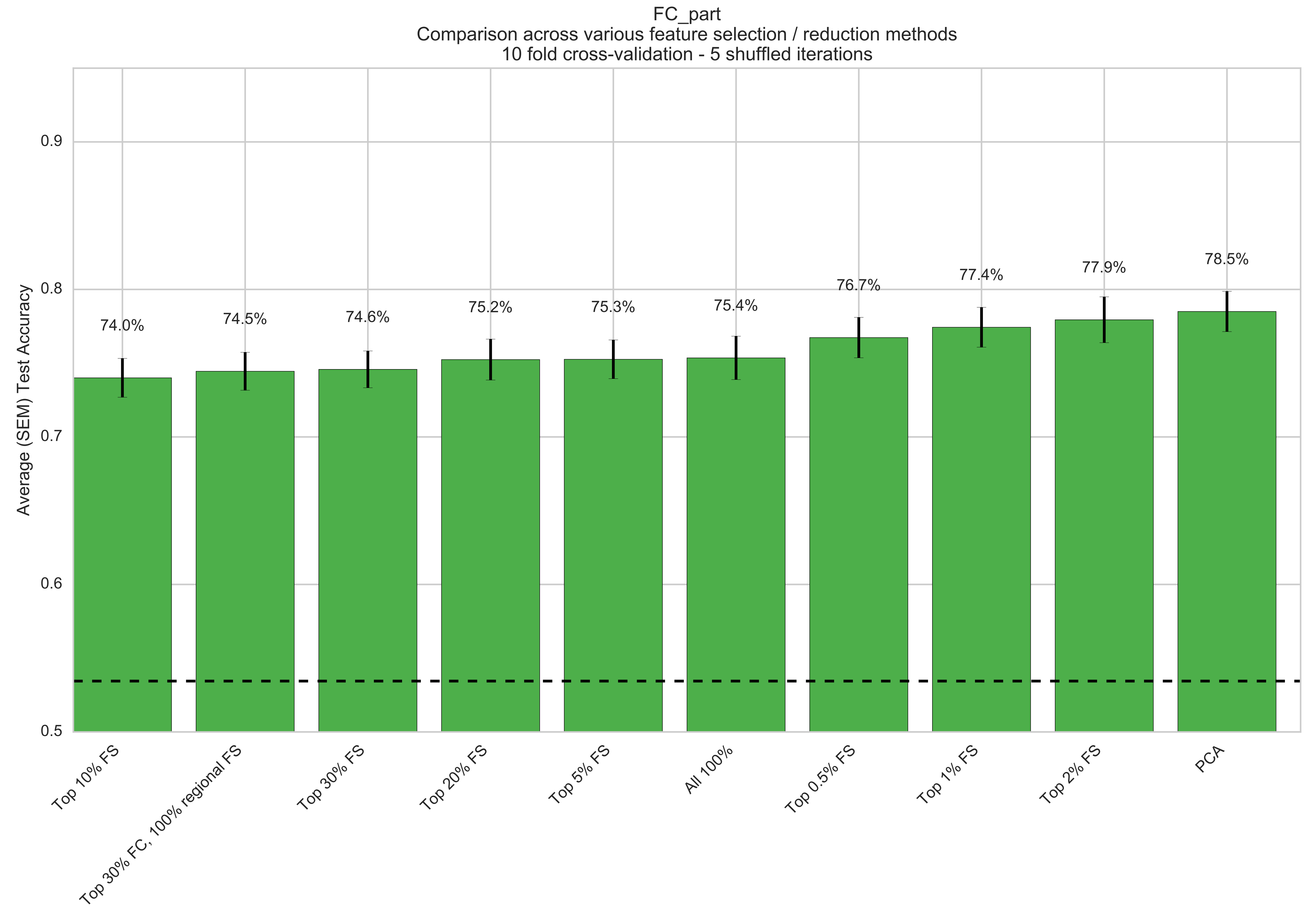
FC_part
Comparison across various feature selection / reduction methods
10 fold cross-validation - 5 shuffled iterations
Average (SEM) Test Accuracy
0.9
0.8
0.7
0.6
0.5
74.0%
74.5%
74.6%
75.2%
75.3%
75.4%
76.7%
77.4%
77.9%
78.5%
Top 10% FS
Top 30% FC, 100% regional FS
Top 30% FS
Top 20% FS
Top 5% FS
All 100%
Top 0.5% FS
Top 1% FS
Top 2% FS
PCA

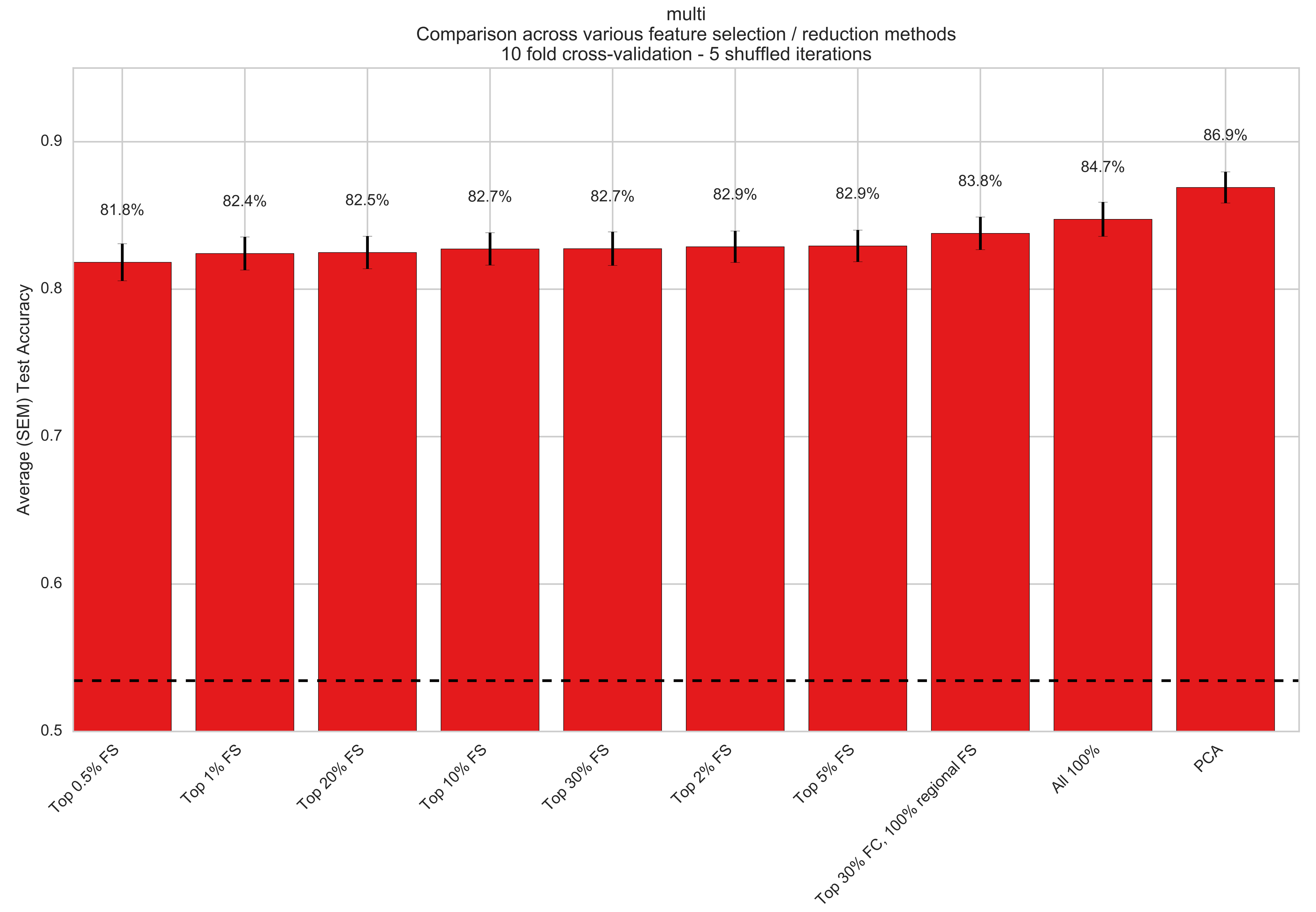

multi
Comparison across various feature selection / reduction methods
10 fold cross-validation - 5 shuffled iterations
Average (SEM) Test Accuracy
0.9
0.8
0.7
0.6
0.5
81.8%
82.4%
82.5%
82.7%
82.7%
82.9%
82.9%
83.8%
84.7%
86.9%
Top 0.5% FS
Top 1% FS
Top 20% FS
Top 10% FS
Top 30% FS
Top 2% FS
Top 5% FS
Top 30% FC, 100% regional FS
All 100%
PCA

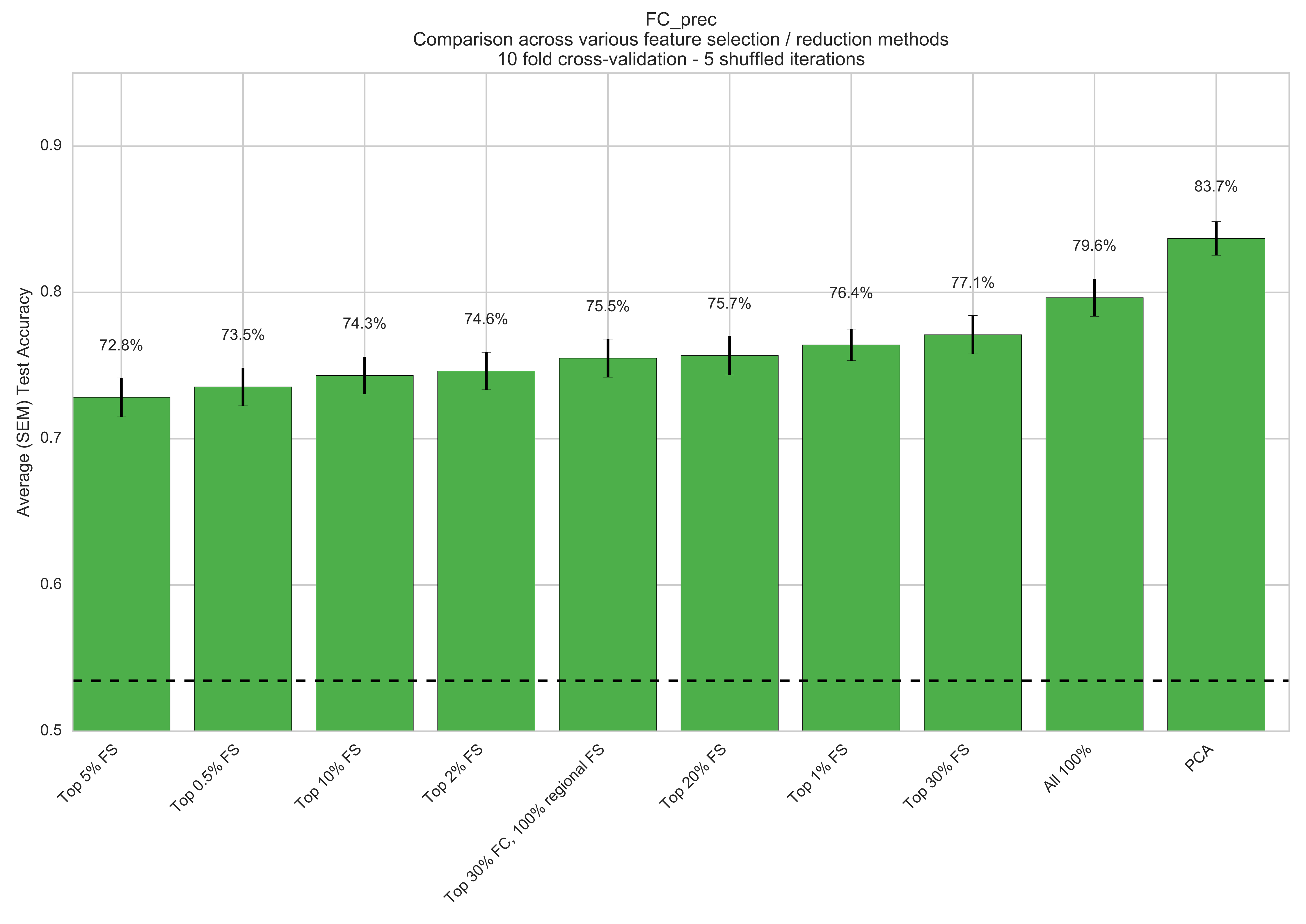
FC_prec
Comparison across various feature selection / reduction methods
10 fold cross-validation - 5 shuffled iterations
Average (SEM) Test Accuracy
0.9
0.8
0.7
0.6
0.5
72.8%
73.5%
74.3%
74.6%
75.5%
75.7%
76.4%
77.1%
79.6%
83.7%
Top 5% FS
Top 0.5% FS
Top 10% FS
Top 2% FS
Top 30% FC, 100% regional FS
Top 20% FS
Top 1% FS
Top 30% FS
All 100%
PCA

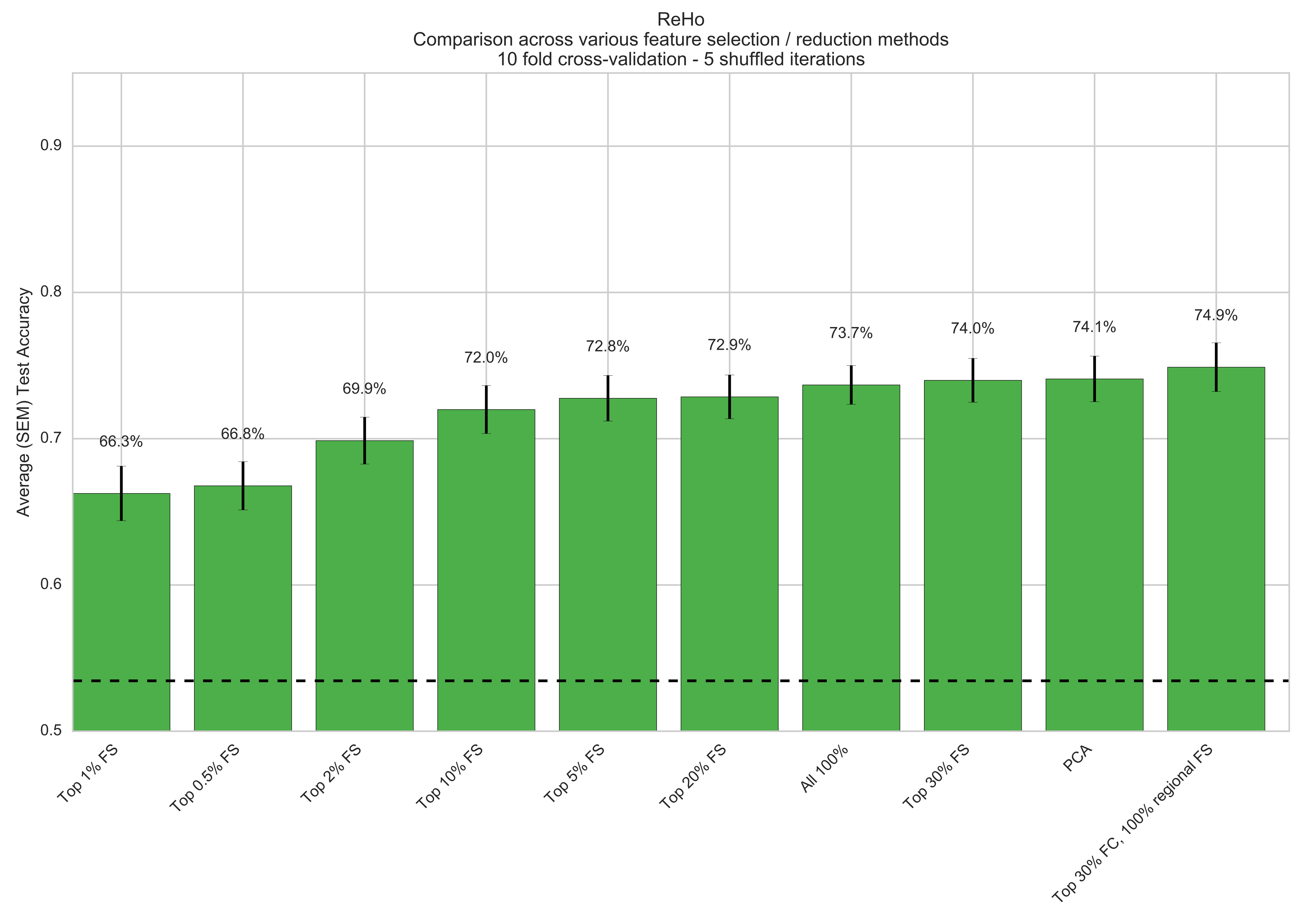
ReHo
Comparison across various feature selection / reduction methods
10 fold cross-validation - 5 shuffled iterations
Average (SEM) Test Accuracy
0.9
0.8
0.7
0.6
0.5
66.3%
66.8%
69.9%
72.0%
72.8%
72.9%
73.7%
74.0%
74.1%
74.9%
Top 1% FS
Top 0.5% FS
Top 2% FS
Top 10% FS
Top 5% FS
Top 20% FS
All 100%
Top 30% FS
PCA
Top 30% FC, 100% regional FS

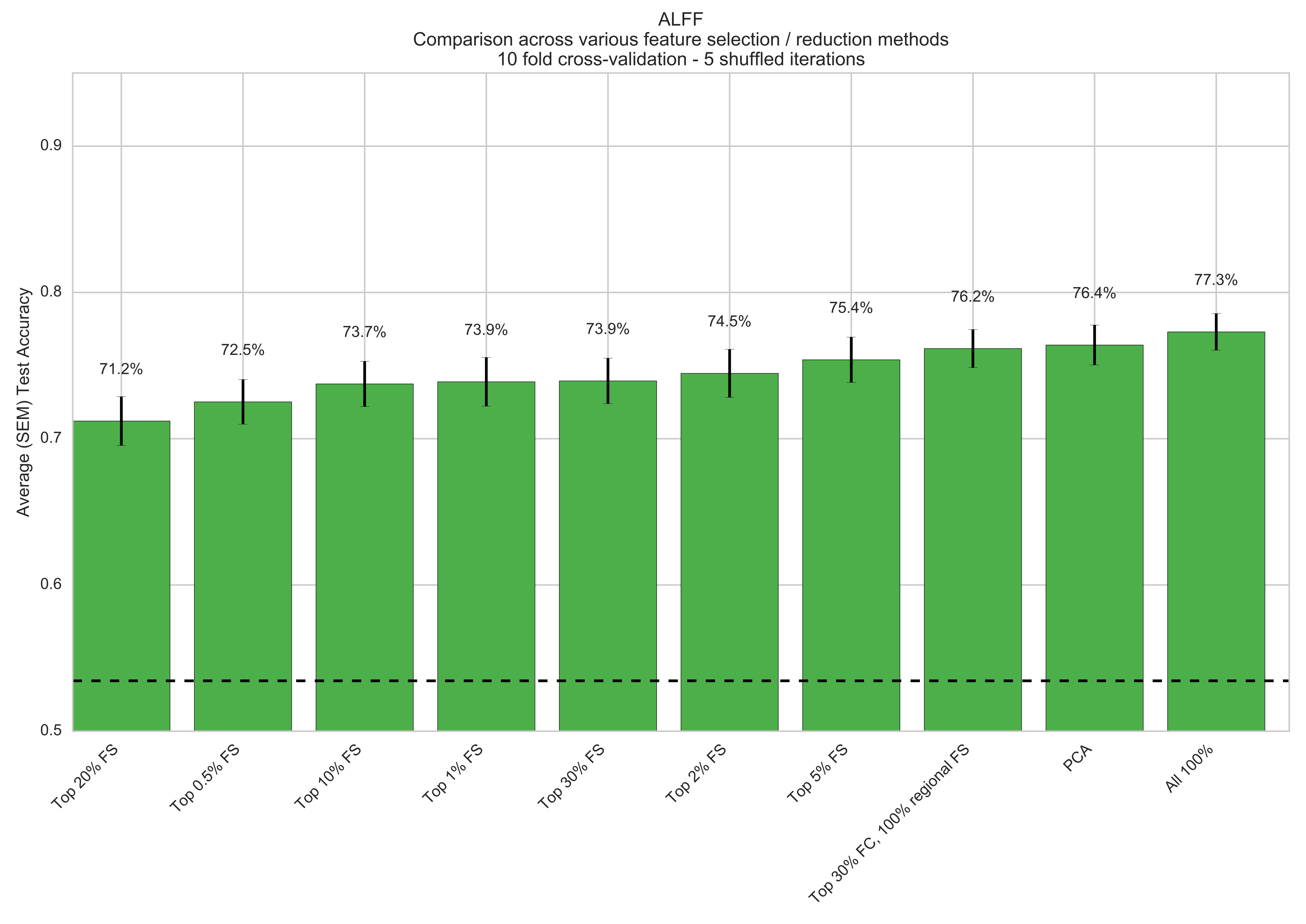

ALFF
Comparison across various feature selection / reduction methods
10 fold cross-validation - 5 shuffled iterations
Average (SEM) Test Accuracy
0.5
0.6
0.7
0.8
0.9
71.2%
72.5%
73.7%
73.9%
73.9%
74.5%
75.4%
76.2%
76.4%
77.3%
Top 20% FS
Top 0.5% FS
Top 10% FS
Top 1% FS
Top 30% FS
Top 2% FS
Top 5% FS
Top 30% FC, 100% regional FS
PCA
All 100%

Comparison of prediction accuracy for stacked models of various feature types.
Final ensemble model shown in red.

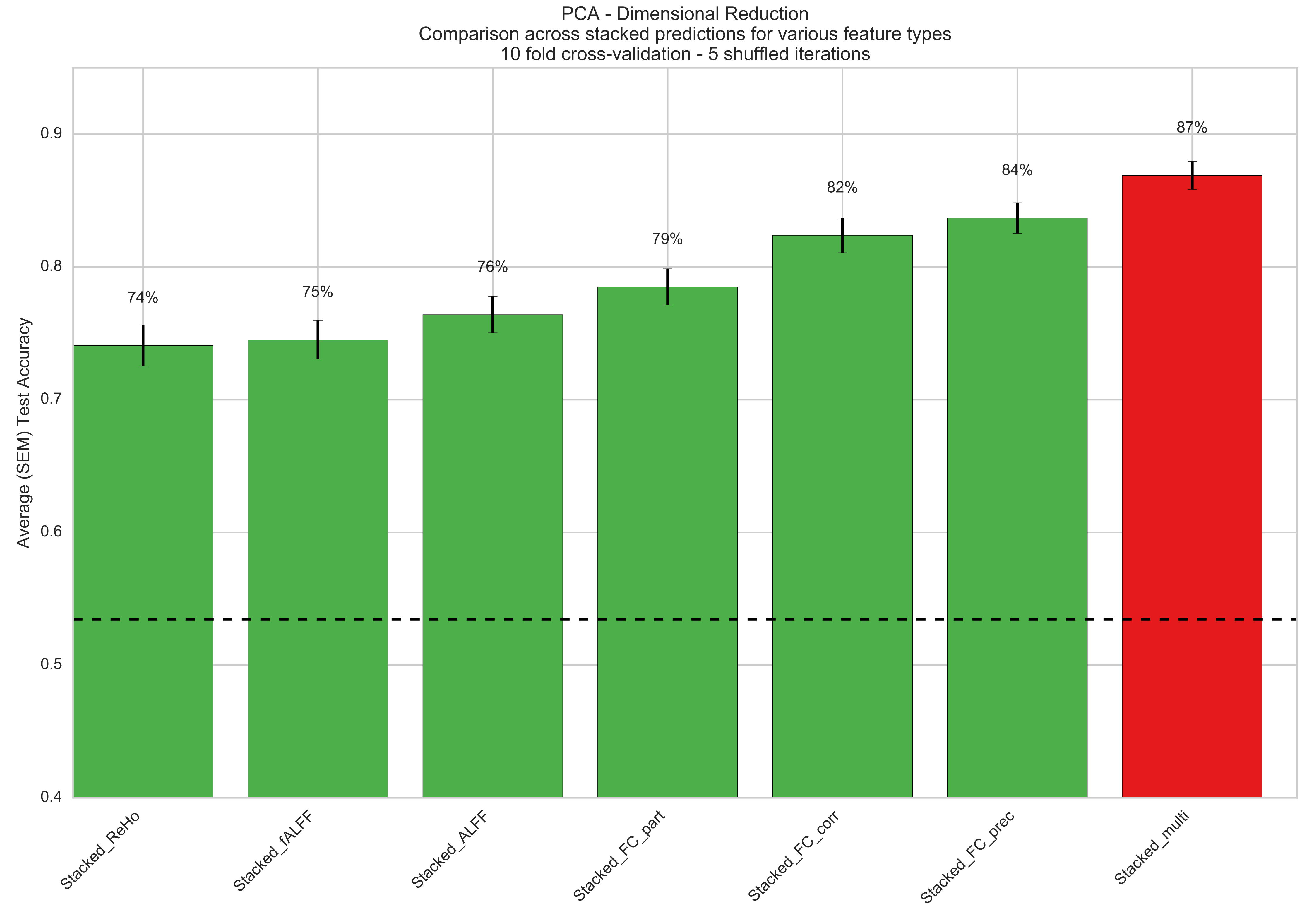
PCA - Dimensional Reduction
Comparison across stacked predictions for various feature types
10 fold cross-validation - 5 shuffled iterations
Average (SEM) Test Accuracy
0.4
0.5
0.6
0.7
0.8
0.9
74%
75%
76%
79%
82%
84%
87%
Stacked_ReHo
Stacked_fALFF
Stacked_ALFF
Stacked_FC_part
Stacked_FC_corr
Stacked_FC_prec
Stacked_multi

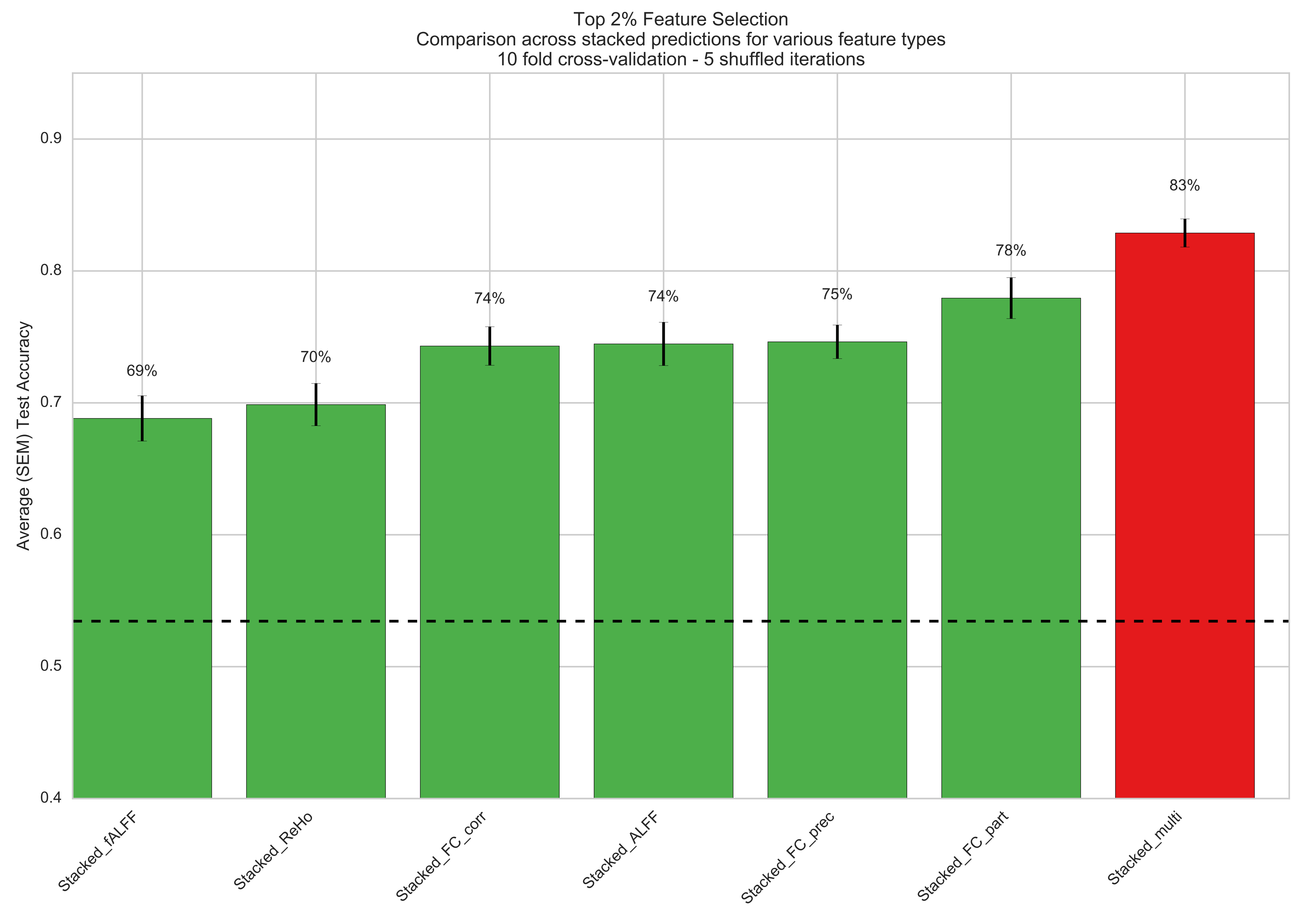
Top 2% Feature Selection
Comparison across stacked predictions for various feature types
10 fold cross-validation - 5 shuffled iterations
Average (SEM) Test Accuracy
0.9
0.8
0.7
0.6
0.5
0.4
69%
70%
74%
74%
75%
78%
83%
Stacked_fALFF
Stacked_ReHo
Stacked_FC_corr
Stacked_ALFF
Stacked_FC_prec
Stacked_FC_part
Stacked_multi

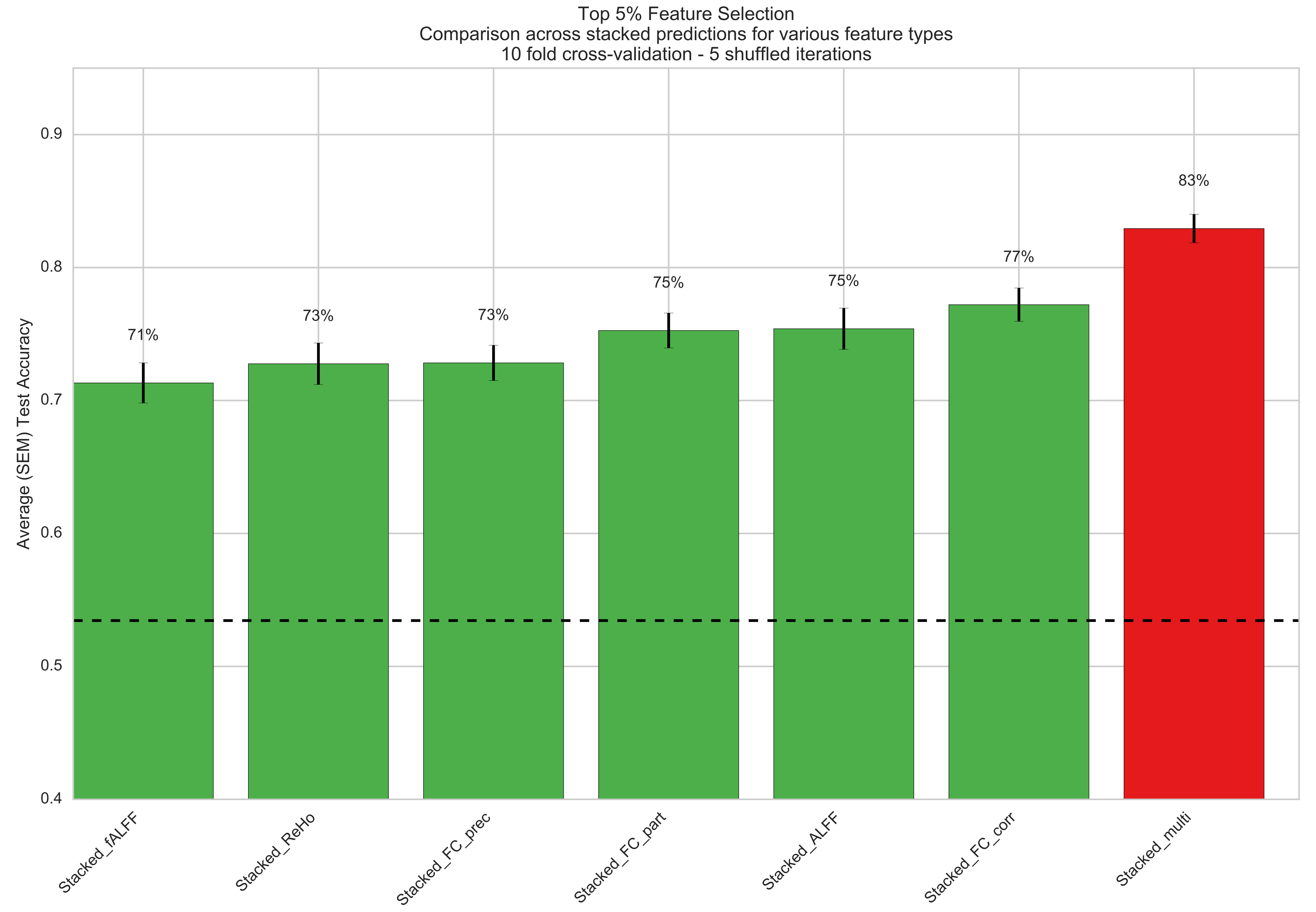

Top 5% Feature Selection
Comparison across stacked predictions for various feature types
10 fold cross-validation - 5 shuffled iterations
Average (SEM) Test Accuracy
0.4
0.5
0.6
0.7
0.8
0.9
71%
73%
73%
75%
75%
77%
83%
Stacked_fALFF
Stacked_ReHo
Stacked_FC_prec
Stacked_FC_part
Stacked_ALFF
Stacked_FC_corr
Stacked_multi

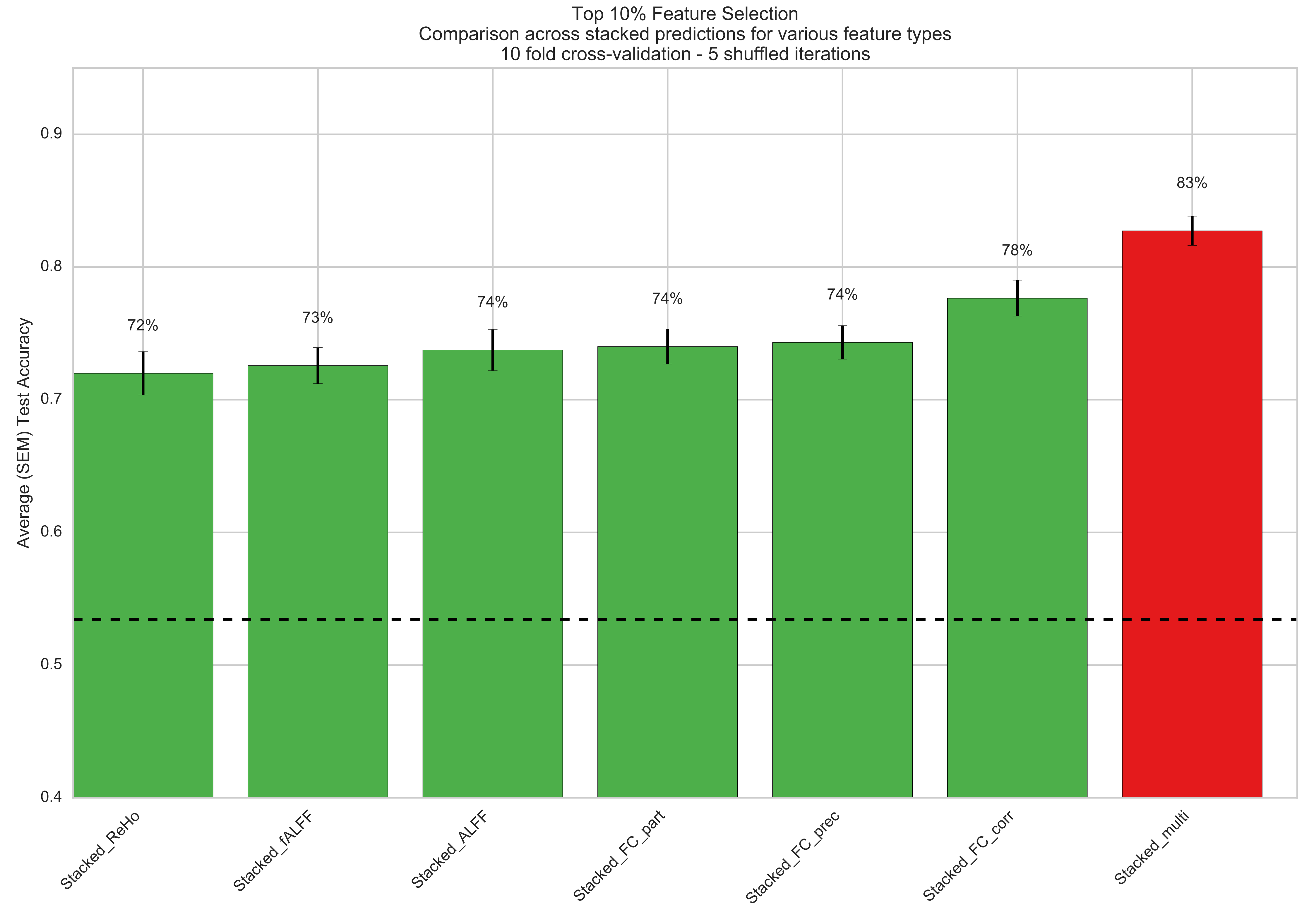

Top 10% Feature Selection
Comparison across stacked predictions for various feature types
10 fold cross-validation - 5 shuffled iterations
Average (SEM) Test Accuracy
0.4
0.5
0.6
0.7
0.8
0.9
72%
73%
74%
74%
74%
78%
83%
Stacked_ReHo
Stacked_fALFF
Stacked_ALFF
Stacked_FC_part
Stacked_FC_prec
Stacked_FC_corr
Stacked_multi

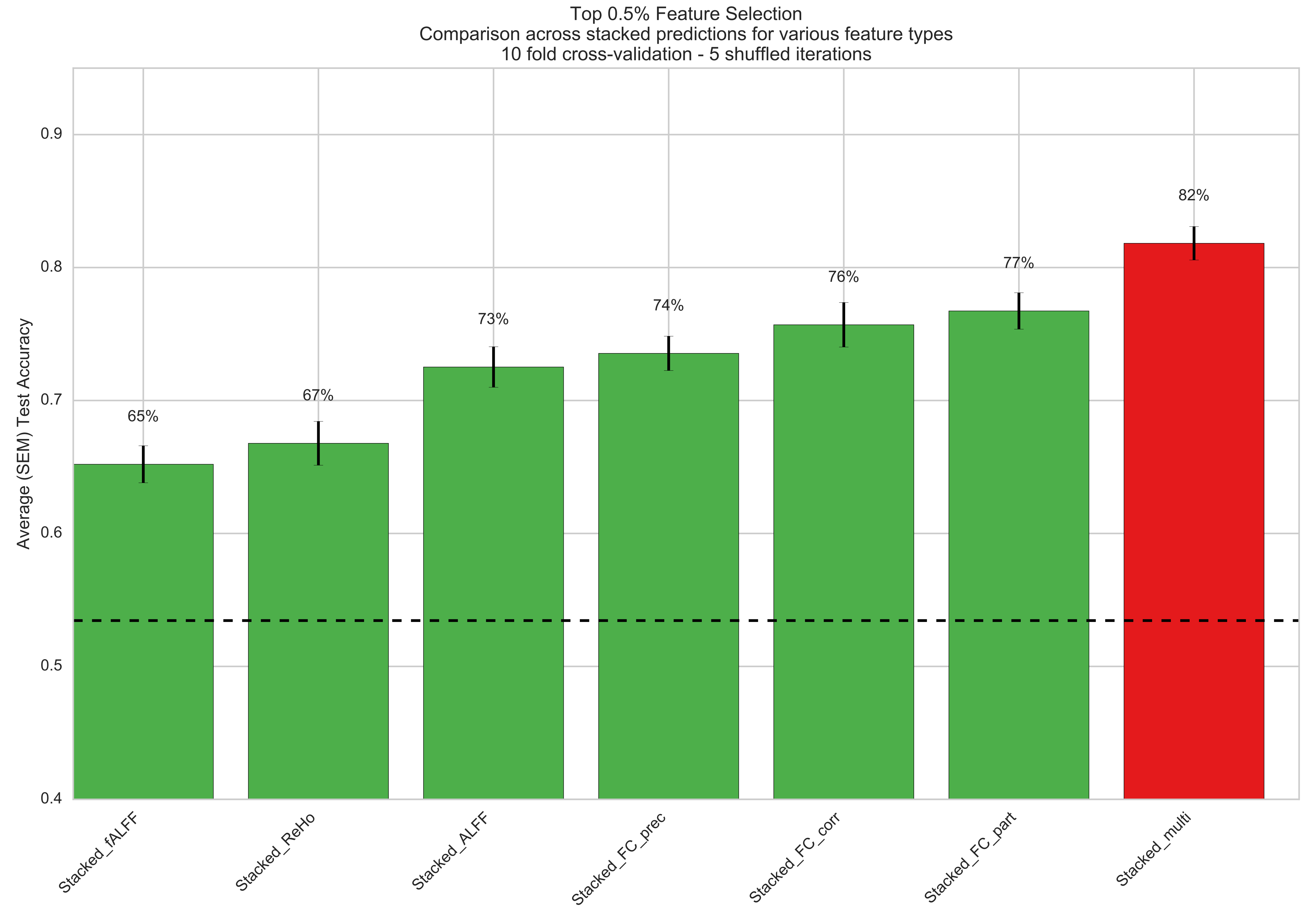

Top 0.5% Feature Selection
Comparison across stacked predictions for various feature types
10 fold cross-validation - 5 shuffled iterations
Average (SEM) Test Accuracy
0.4
0.5
0.6
0.7
0.8
0.9
65%
67%
73%
74%
76%
77%
82%
Stacked_fALFF
Stacked_ReHo
Stacked_ALFF
Stacked_FC_prec
Stacked_FC_corr
Stacked_FC_part
Stacked_multi

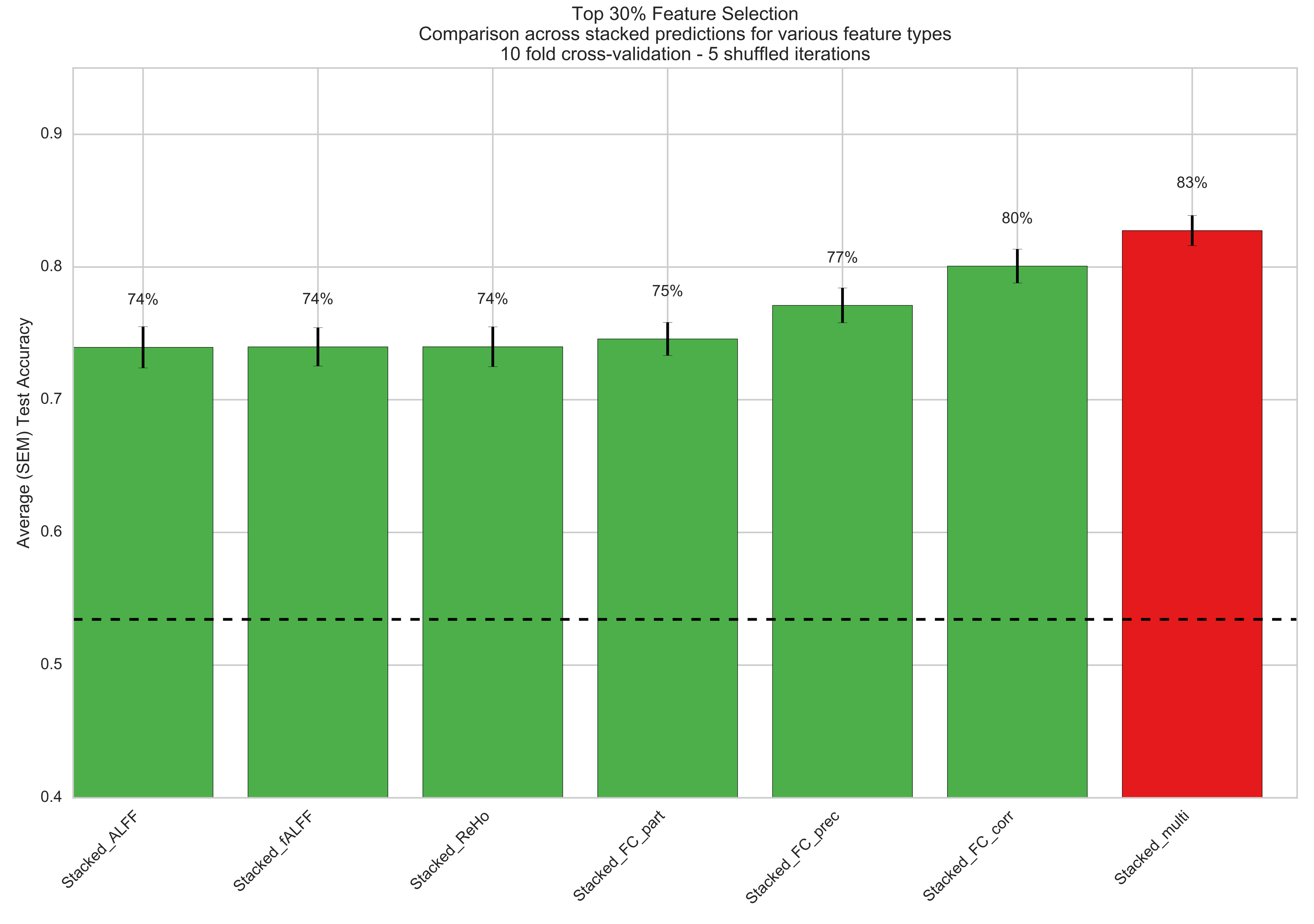
Top 30% Feature Selection
Comparison across stacked predictions for various feature types
10 fold cross-validation - 5 shuffled iterations
Average (SEM) Test Accuracy
0.9
0.8
0.7
0.6
0.5
0.4
74%
74%
74%
75%
77%
80%
83%
Stacked_ALFF
Stacked_fALFF
Stacked_ReHo
Stacked_FC_part
Stacked_FC_prec
Stacked_FC_corr
Stacked_multi

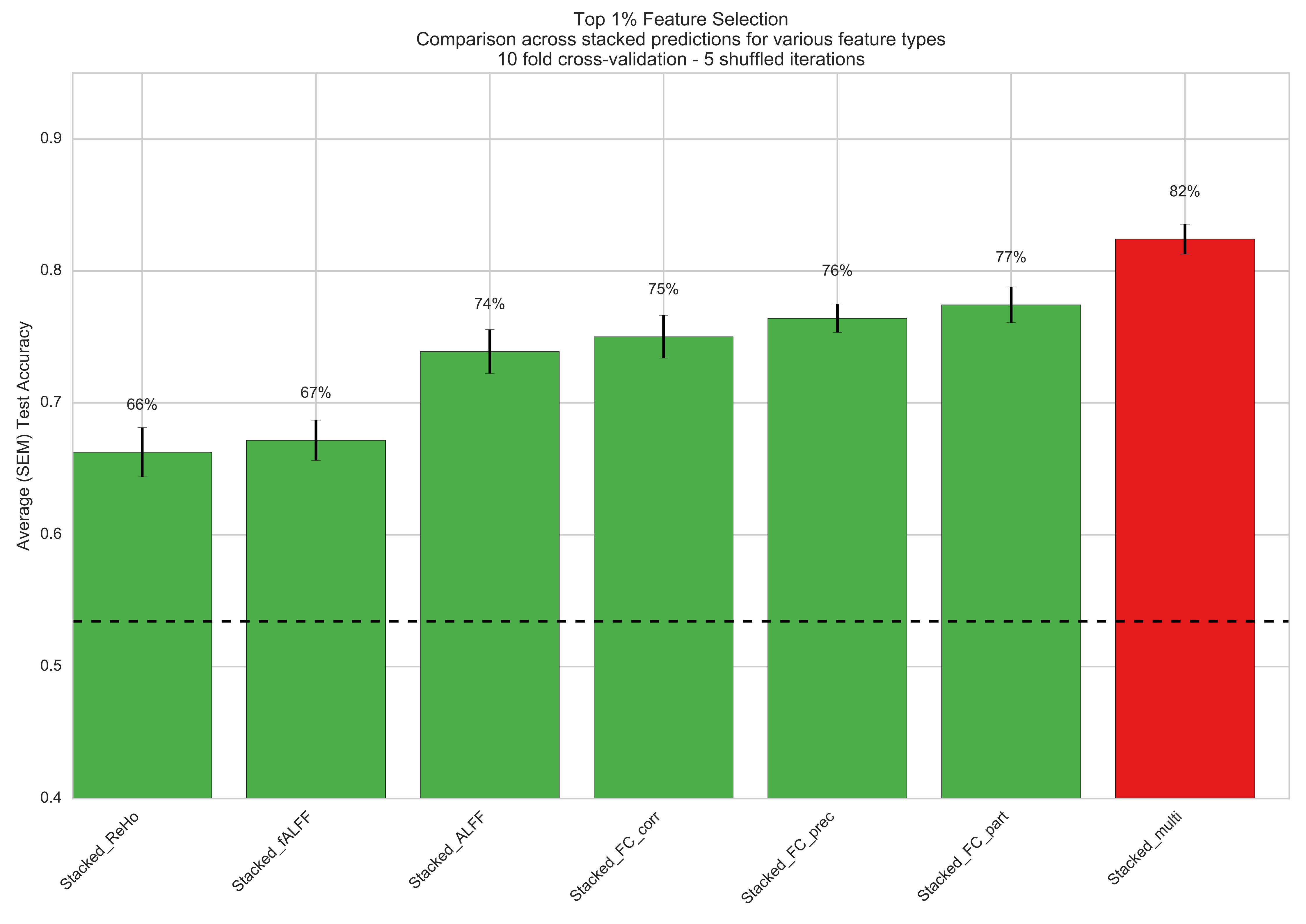

Top 1% Feature Selection
Comparison across stacked predictions for various feature types
10 fold cross-validation - 5 shuffled iterations
Average (SEM) Test Accuracy
0.4
0.5
0.6
0.7
0.8
0.9
66%
67%
74%
75%
76%
77%
82%
Stacked_ReHo
Stacked_fALFF
Stacked_ALFF
Stacked_FC_corr
Stacked_FC_prec
Stacked_FC_part
Stacked_multi

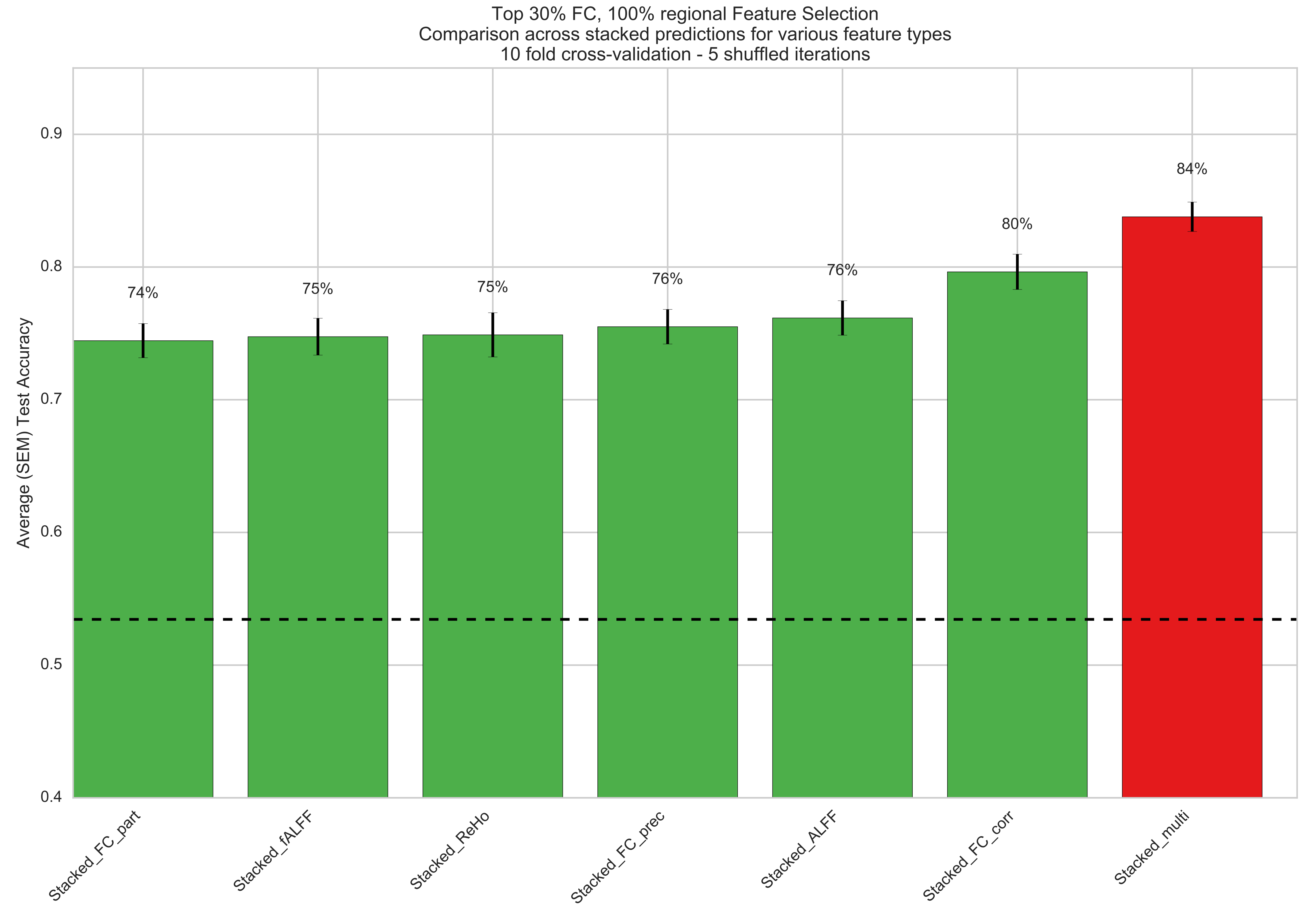
Top 30% FC, 100% regional Feature Selection
Comparison across stacked predictions for various feature types
10 fold cross-validation - 5 shuffled iterations
Average (SEM) Test Accuracy
0.4
0.5
0.6
0.7
0.8
0.9
74%
75%
75%
76%
76%
80%
84%
Stacked_FC_part
Stacked_fALFF
Stacked_ReHo
Stacked_FC_prec
Stacked_ALFF
Stacked_FC_corr
Stacked_multi

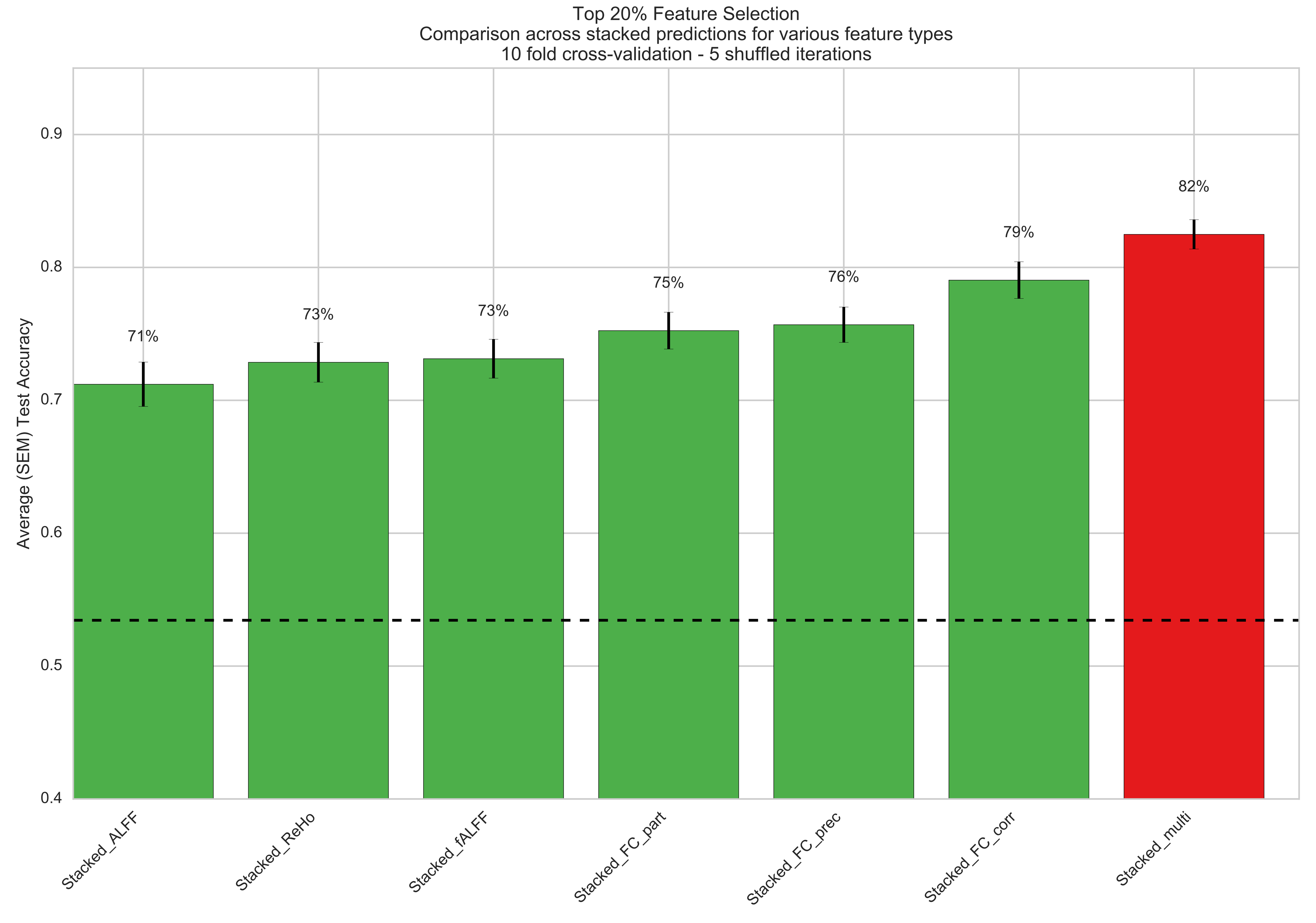
Top 20% Feature Selection
Comparison across stacked predictions for various feature types
10 fold cross-validation - 5 shuffled iterations
Average (SEM) Test Accuracy
0.4
0.5
0.6
0.7
0.8
0.9
71%
73%
73%
75%
76%
79%
82%
Stacked_ALFF
Stacked_ReHo
Stacked_fALFF
Stacked_FC_part
Stacked_FC_prec
Stacked_FC_corr
Stacked_multi

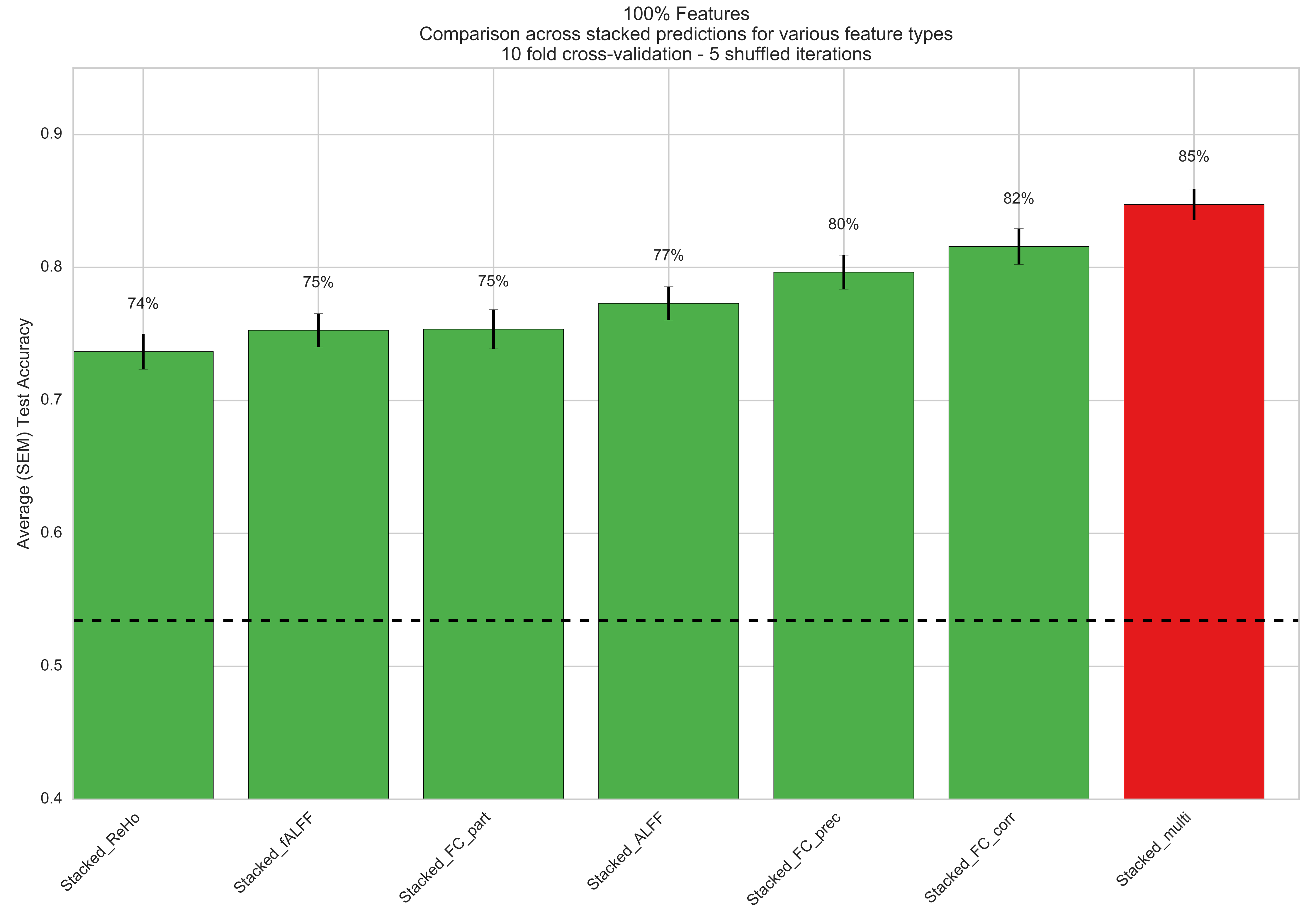

100% Features
Comparison across stacked predictions for various feature types
10 fold cross-validation - 5 shuffled iterations
Average (SEM) Test Accuracy
0.9
0.8
0.7
0.6
0.5
0.4
74%
75%
75%
77%
80%
82%
85%
Stacked_ReHo
Stacked_fALFF
Stacked_FC_part
Stacked_ALFF
Stacked_FC_prec
Stacked_FC_corr
Stacked_multi

Comparison of prediction accuracy for single-source models (blue) and stacked model (green) for a feature type.
Final ensemble model shown in red

# Feature selection / reduction method :PCA - Dimensional Reduction

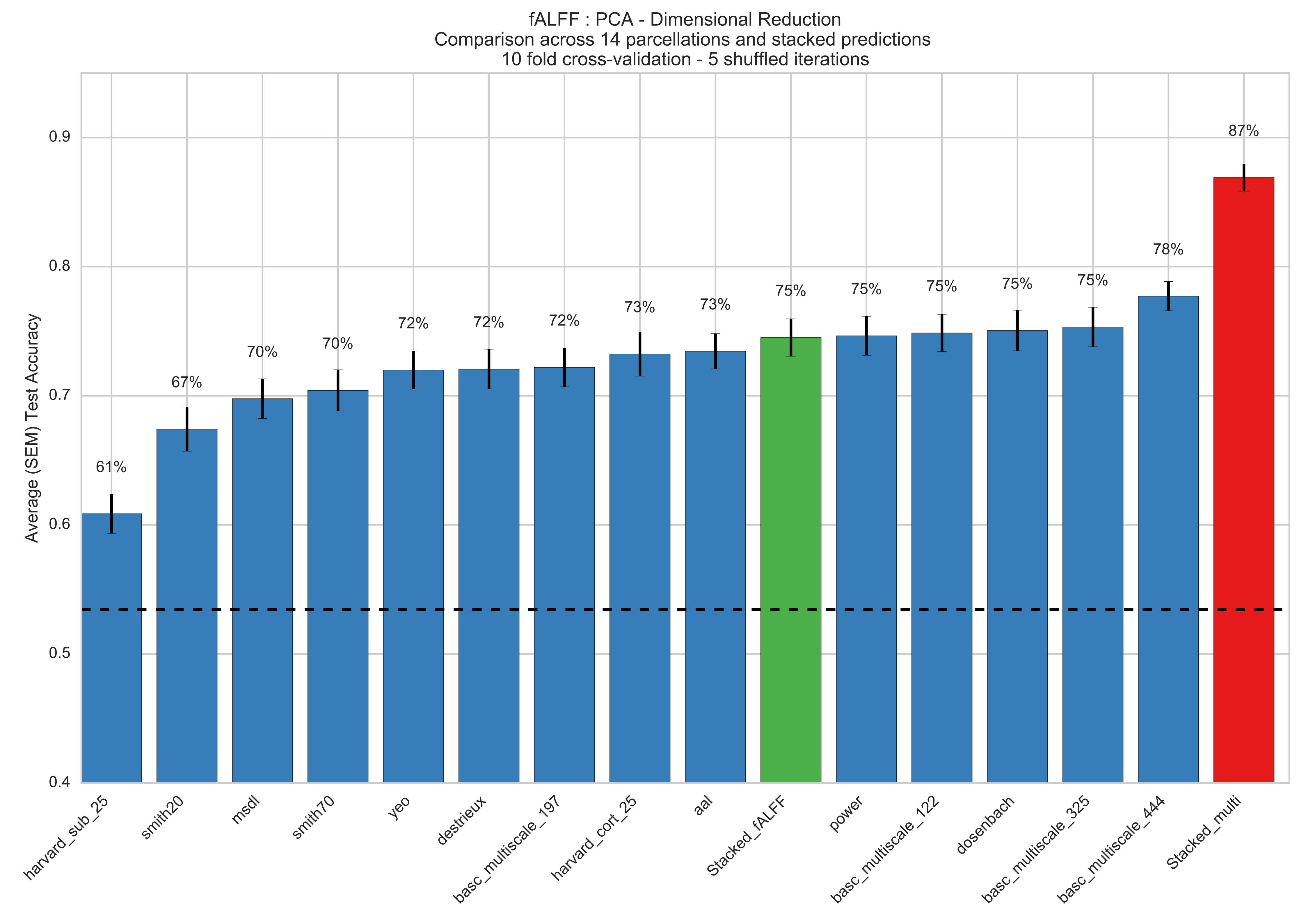
fALFF : PCA - Dimensional Reduction
Comparison across 14 parcellations and stacked predictions
10 fold cross-validation - 5 shuffled iterations
Average (SEM) Test Accuracy
0.4
0.5
0.6
0.7
0.8
0.9
61%
67%
70%
70%
72%
72%
72%
73%
73%
75%
75%
75%
75%
75%
78%
87%
harvard_sub_25
smith20
msdl
smith70
yeo
destrieux
basc_multiscale_197
harvard_cort_25
aal
Stacked_fALFF
power
basc_multiscale_122
dosenbach
basc_multiscale_325
basc_multiscale_444
Stacked_multi

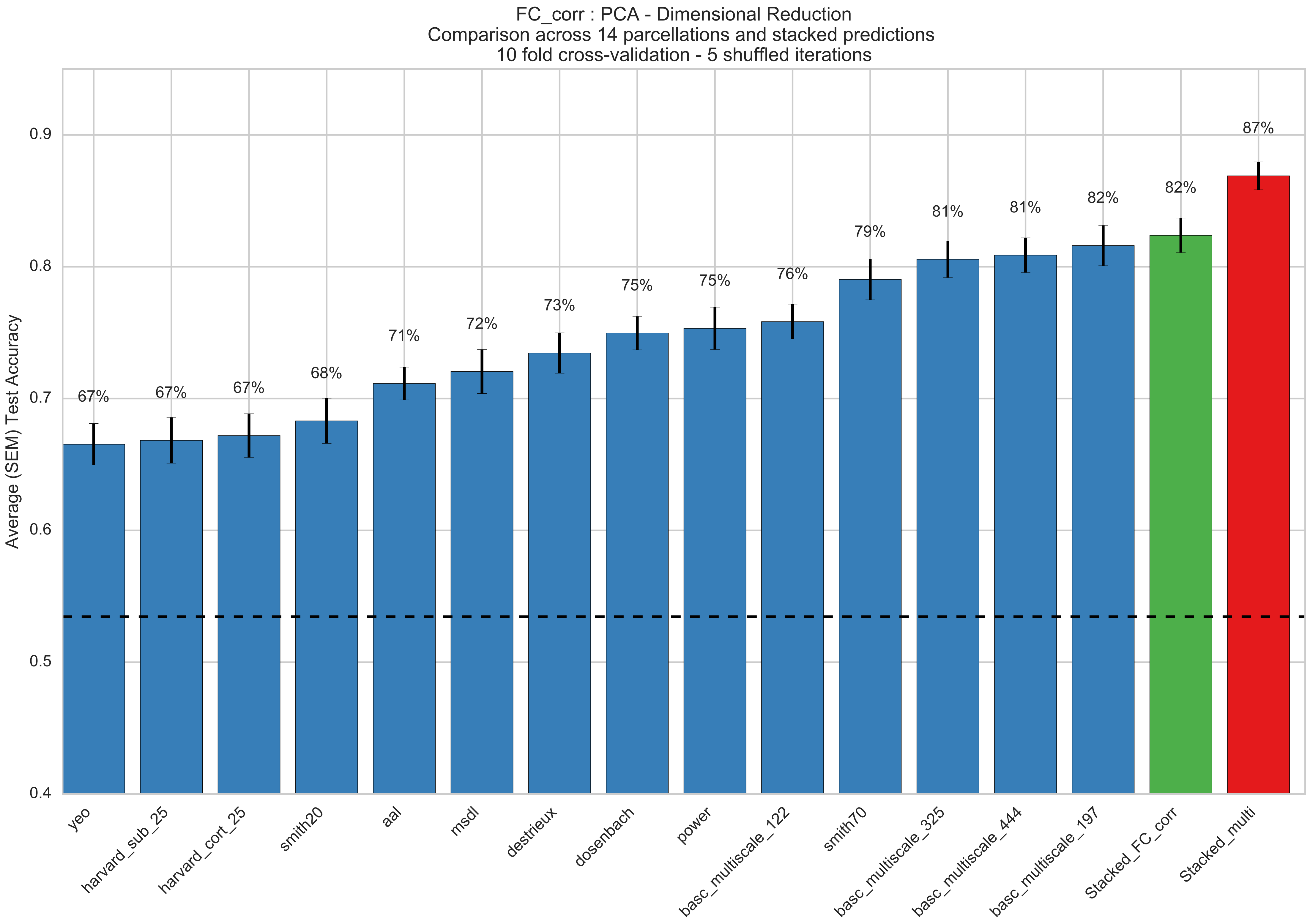
FC_corr : PCA - Dimensional Reduction
Comparison across 14 parcellations and stacked predictions
10 fold cross-validation - 5 shuffled iterations
Average (SEM) Test Accuracy
0.4
0.5
0.6
0.7
0.8
0.9
67%
67%
67%
68%
71%
72%
73%
75%
75%
76%
79%
81%
81%
82%
82%
87%
yeo
harvard_sub_25
harvard_cort_25
smith20
aal
msdl
destrieux
dosenbach
power
basc_multiscale_122
smith70
basc_multiscale_325
basc_multiscale_444
basc_multiscale_197
Stacked_FC_corr
Stacked_multi

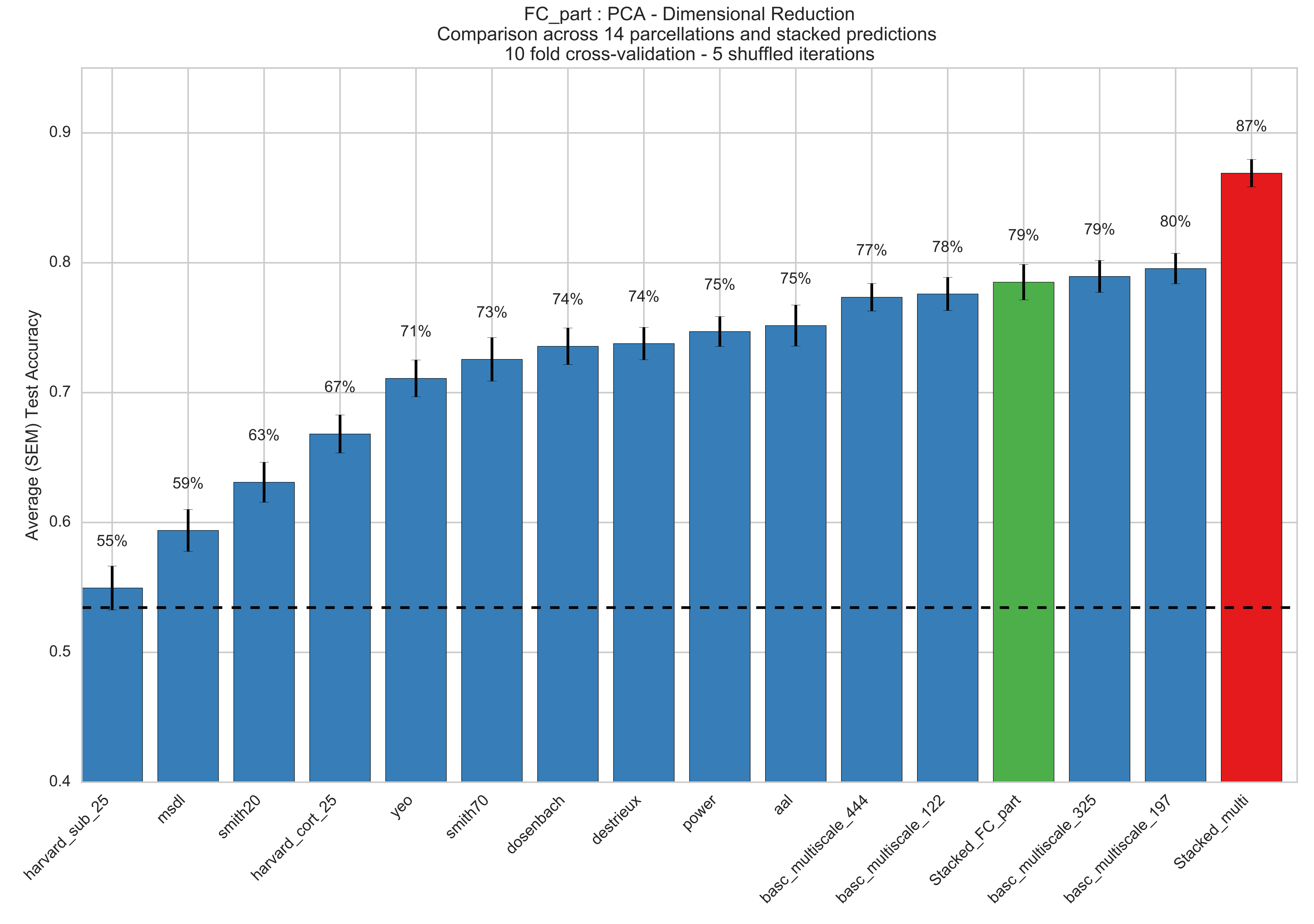

FC_part : PCA - Dimensional Reduction
Comparison across 14 parcellations and stacked predictions
10 fold cross-validation - 5 shuffled iterations
Average (SEM) Test Accuracy
0.4
0.5
0.6
0.7
0.8
0.9
55%
59%
63%
67%
71%
73%
74%
74%
75%
75%
77%
78%
79%
79%
80%
87%
harvard_sub_25
msdl
smith20
harvard_cort_25
yeo
smith70
dosenbach
destrieux
power
aal
basc_multiscale_444
basc_multiscale_122
Stacked_FC_part
basc_multiscale_325
basc_multiscale_197
Stacked_multi

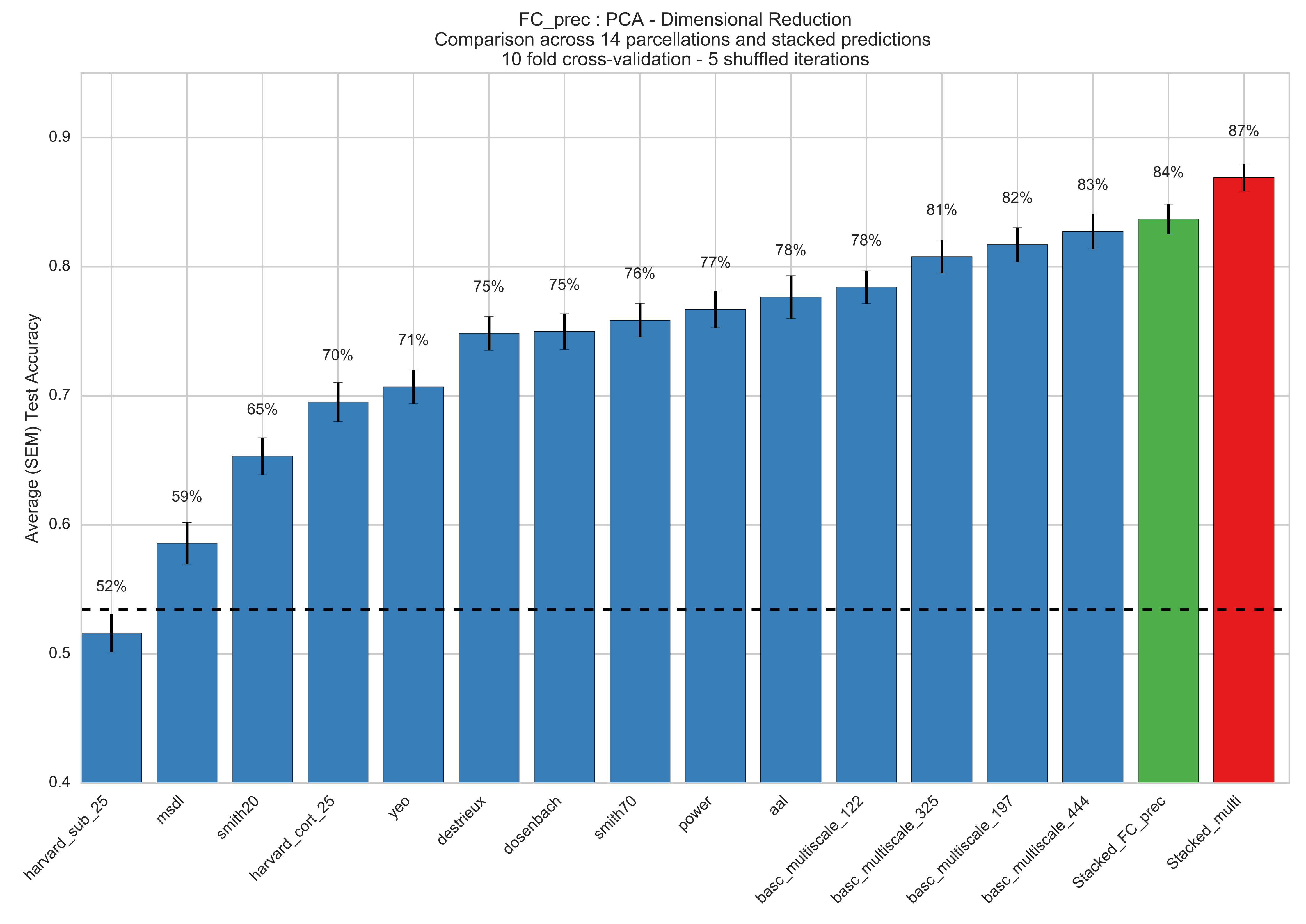

FC_prec : PCA - Dimensional Reduction
Comparison across 14 parcellations and stacked predictions
10 fold cross-validation - 5 shuffled iterations
Average (SEM) Test Accuracy
0.4
0.5
0.6
0.7
0.8
0.9
52%
59%
65%
70%
71%
75%
75%
76%
77%
78%
78%
81%
82%
83%
84%
87%
harvard_sub_25
msdl
smith20
harvard_cort_25
yeo
destrieux
dosenbach
smith70
power
aal
basc_multiscale_122
basc_multiscale_325
basc_multiscale_197
basc_multiscale_444
Stacked_FC_prec
Stacked_multi

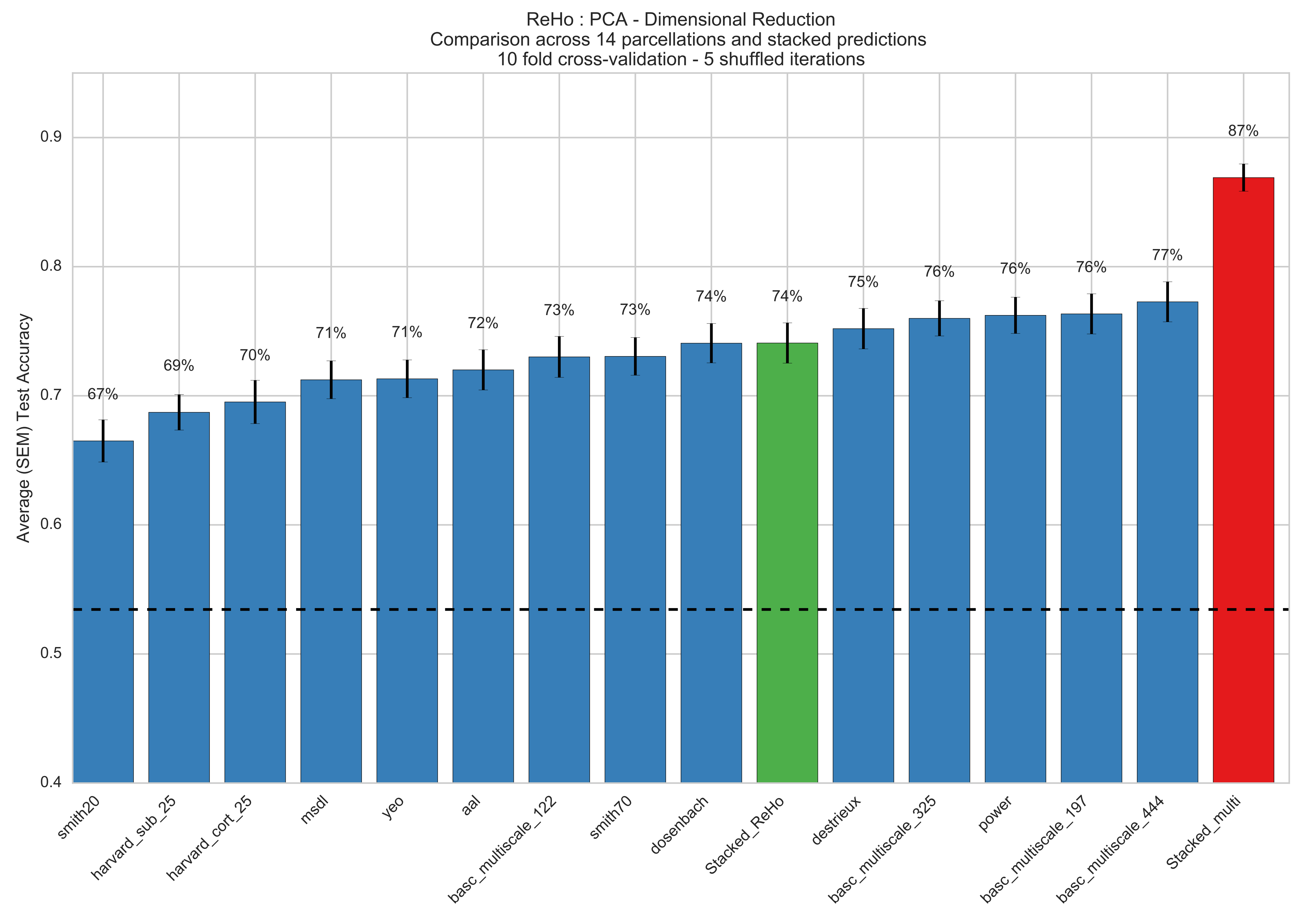
ReHo : PCA - Dimensional Reduction
Comparison across 14 parcellations and stacked predictions
10 fold cross-validation - 5 shuffled iterations
Average (SEM) Test Accuracy
0.4
0.5
0.6
0.7
0.8
0.9
67%
69%
70%
71%
71%
72%
73%
73%
74%
74%
75%
76%
76%
76%
77%
87%
smith20
harvard_sub_25
harvard_cort_25
msdl
yeo
aal
basc_multiscale_122
smith70
dosenbach
Stacked_ReHo
destrieux
basc_multiscale_325
power
basc_multiscale_197
basc_multiscale_444
Stacked_multi

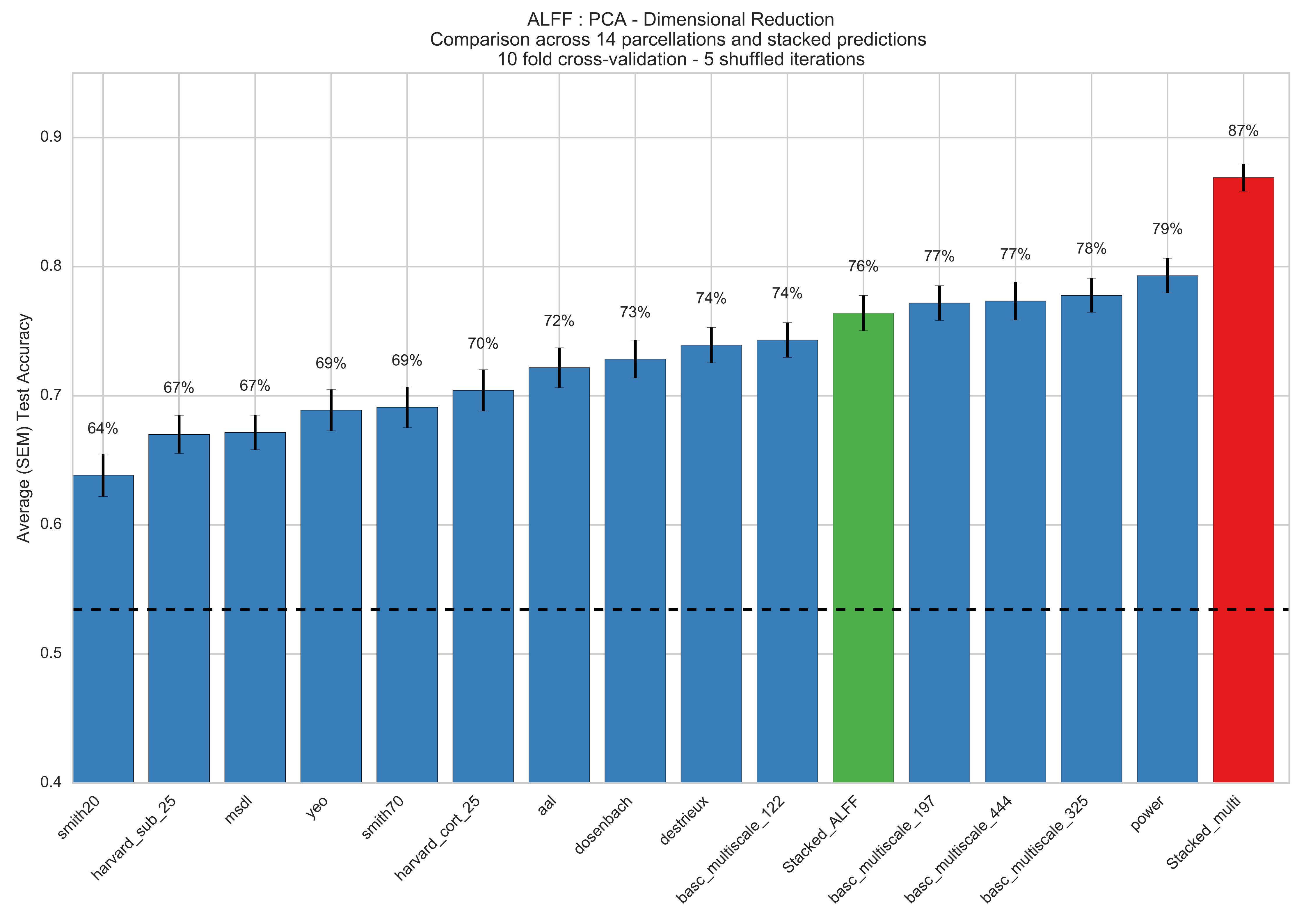
ALFF : PCA - Dimensional Reduction
Comparison across 14 parcellations and stacked predictions
10 fold cross-validation - 5 shuffled iterations
Average (SEM) Test Accuracy
0.4
0.5
0.6
0.7
0.8
0.9
64%
67%
67%
69%
69%
70%
72%
73%
74%
74%
76%
77%
77%
78%
79%
87%
smith20
harvard_sub_25
msdl
yeo
smith70
harvard_cort_25
aal
dosenbach
destrieux
basc_multiscale_122
Stacked_ALFF
basc_multiscale_197
basc_multiscale_444
basc_multiscale_325
power
Stacked_multi

Feature selection / reduction method :Top 2% Feature Selection

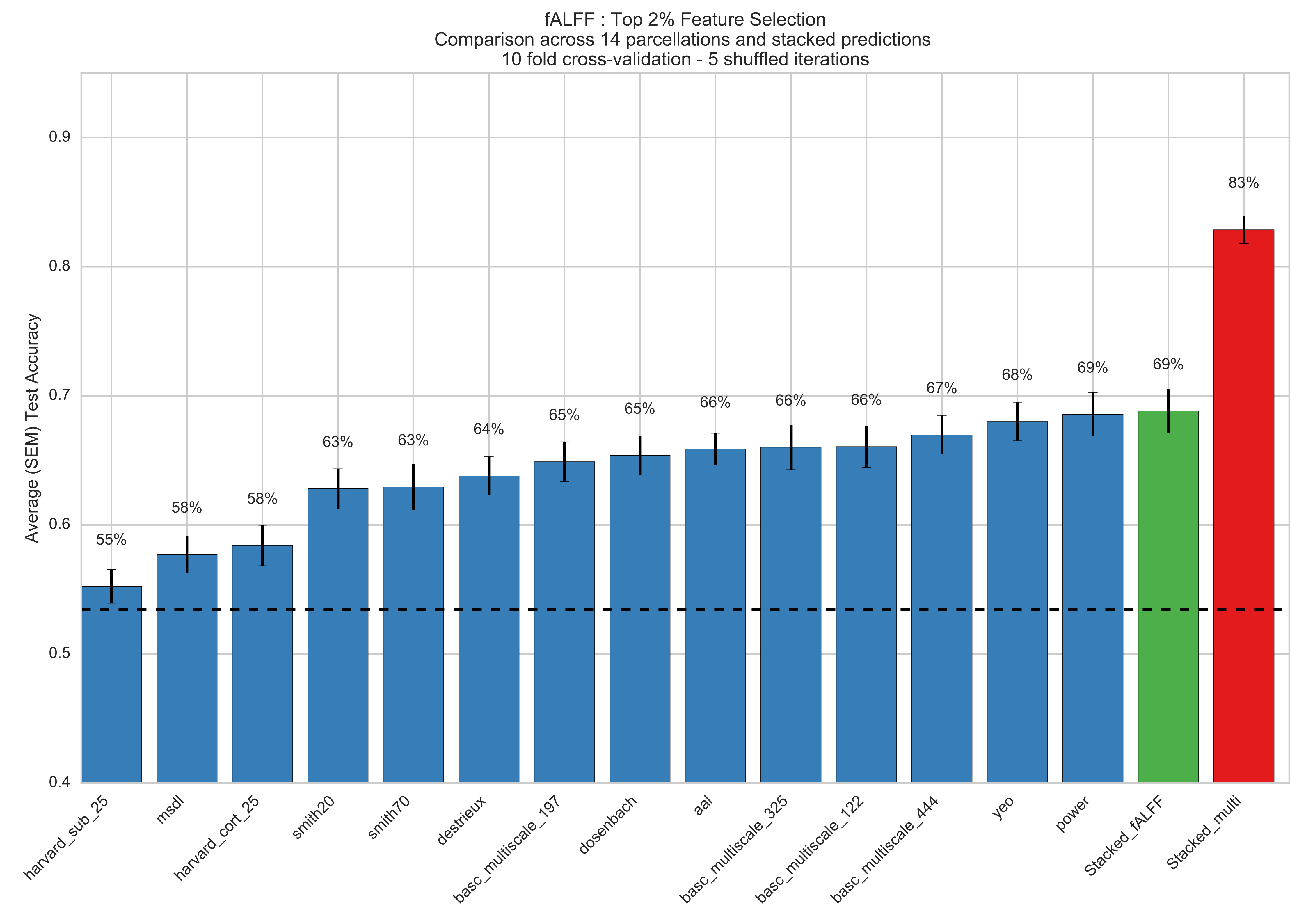

fALFF : Top 2% Feature Selection
Comparison across 14 parcellations and stacked predictions
10 fold cross-validation - 5 shuffled iterations
Average (SEM) Test Accuracy
0.9
0.8
0.7
0.6
0.5
0.4
55%
58%
58%
63%
63%
64%
65%
65%
66%
66%
66%
67%
68%
69%
69%
83%
harvard_sub_25
msdl
harvard_cort_25
smith20
smith70
destrieux
basc_multiscale_197
dosenbach
aal
basc_multiscale_325
basc_multiscale_122
basc_multiscale_444
yeo
power
Stacked_fALFF
Stacked_multi

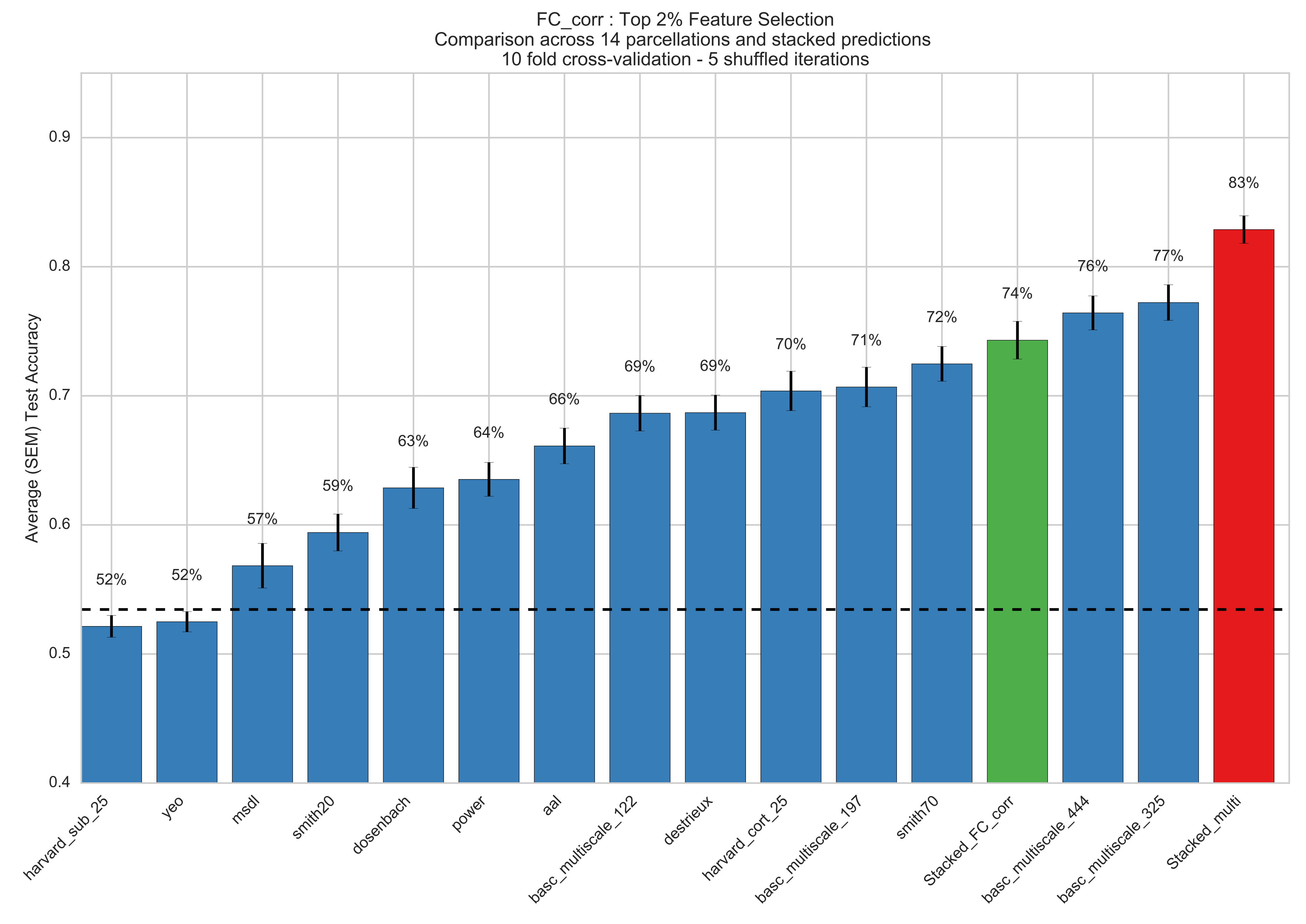
FC_corr : Top 2% Feature Selection
Comparison across 14 parcellations and stacked predictions
10 fold cross-validation - 5 shuffled iterations
Average (SEM) Test Accuracy
0.4
0.5
0.6
0.7
0.8
0.9
52%
52%
57%
59%
63%
64%
66%
69%
69%
70%
71%
72%
74%
76%
77%
83%
harvard_sub_25
yeo
msdl
smith20
dosenbach
power
aal
basc_multiscale_122
destrieux
harvard_cort_25
basc_multiscale_197
smith70
Stacked_FC_corr
basc_multiscale_444
basc_multiscale_325
Stacked_multi

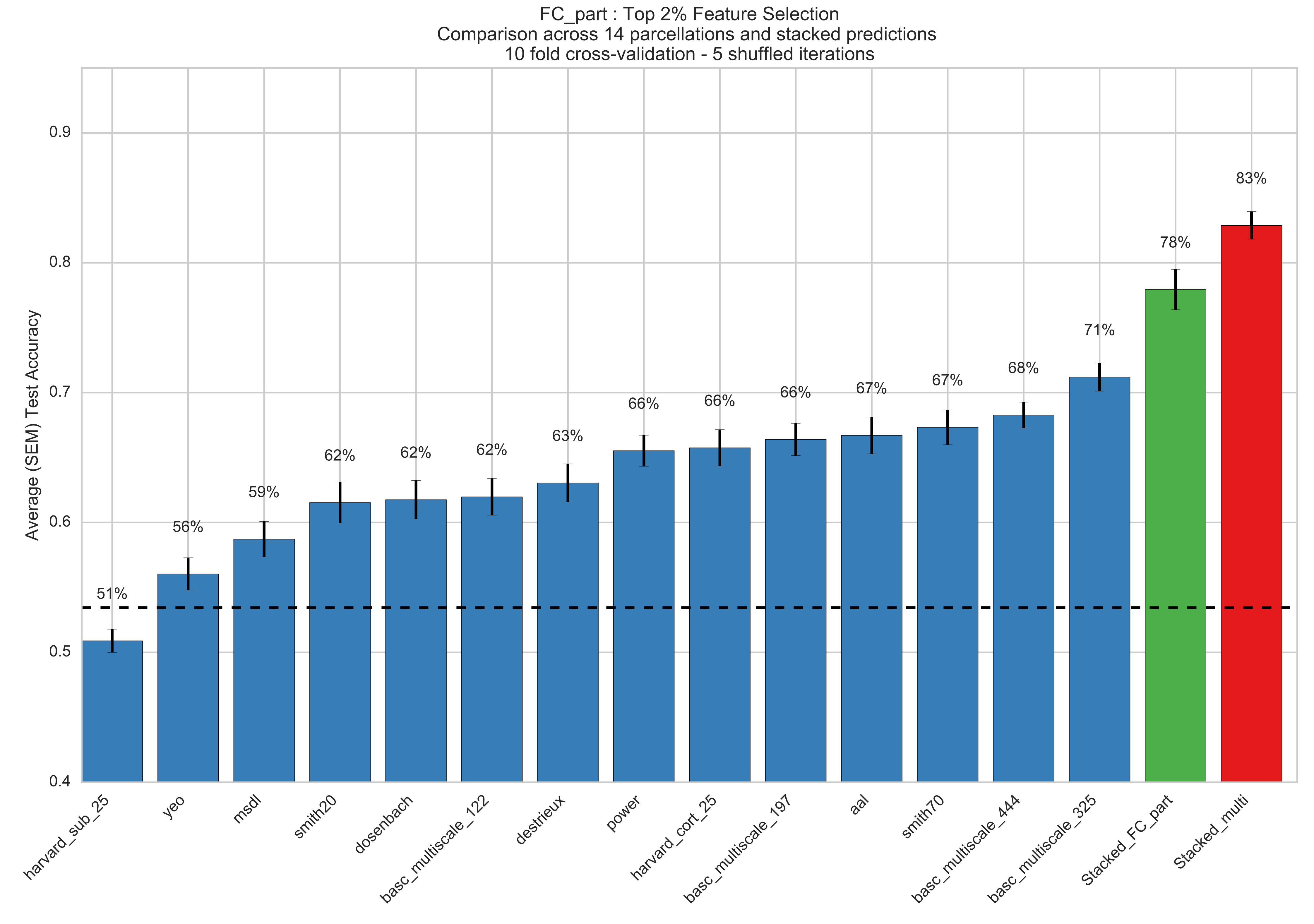
FC_part : Top 2% Feature Selection
Comparison across 14 parcellations and stacked predictions
10 fold cross-validation - 5 shuffled iterations
Average (SEM) Test Accuracy
0.4
0.5
0.6
0.7
0.8
0.9
51%
56%
59%
62%
62%
62%
63%
66%
66%
66%
67%
67%
68%
71%
78%
83%
harvard_sub_25
yeo
msdl
smith20
dosenbach
basc_multiscale_122
destrieux
power
harvard_cort_25
basc_multiscale_197
aal
smith70
basc_multiscale_444
basc_multiscale_325
Stacked_FC_part
Stacked_multi

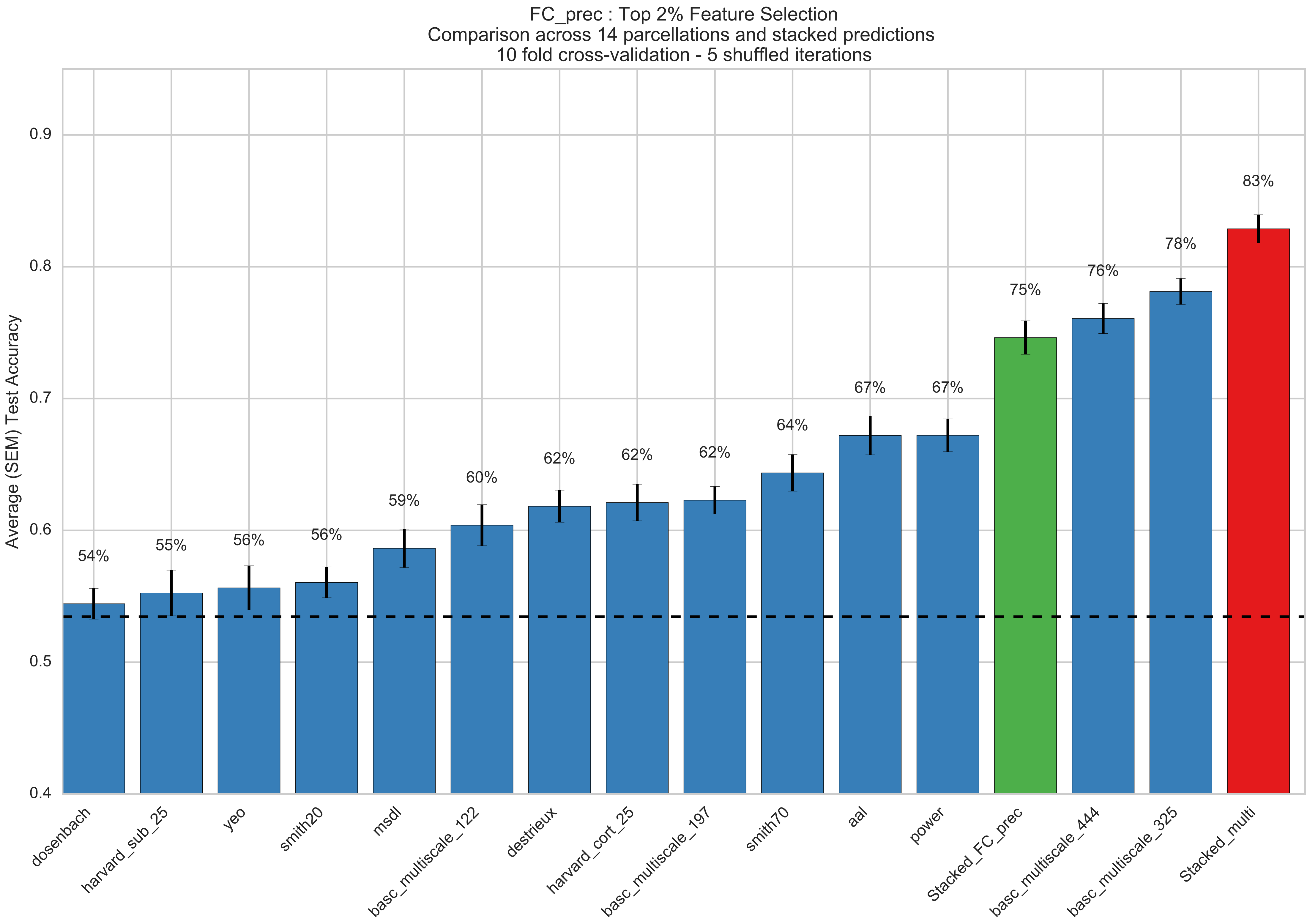

FC_prec : Top 2% Feature Selection
Comparison across 14 parcellations and stacked predictions
10 fold cross-validation - 5 shuffled iterations
Average (SEM) Test Accuracy
0.4
0.5
0.6
0.7
0.8
0.9
54%
55%
56%
56%
59%
60%
62%
62%
62%
64%
67%
67%
75%
76%
78%
83%
dosenbach
harvard_sub_25
yeo
smith20
msdl
basc_multiscale_122
destrieux
harvard_cort_25
basc_multiscale_197
smith70
aal
power
Stacked_FC_prec
basc_multiscale_444
basc_multiscale_325
Stacked_multi

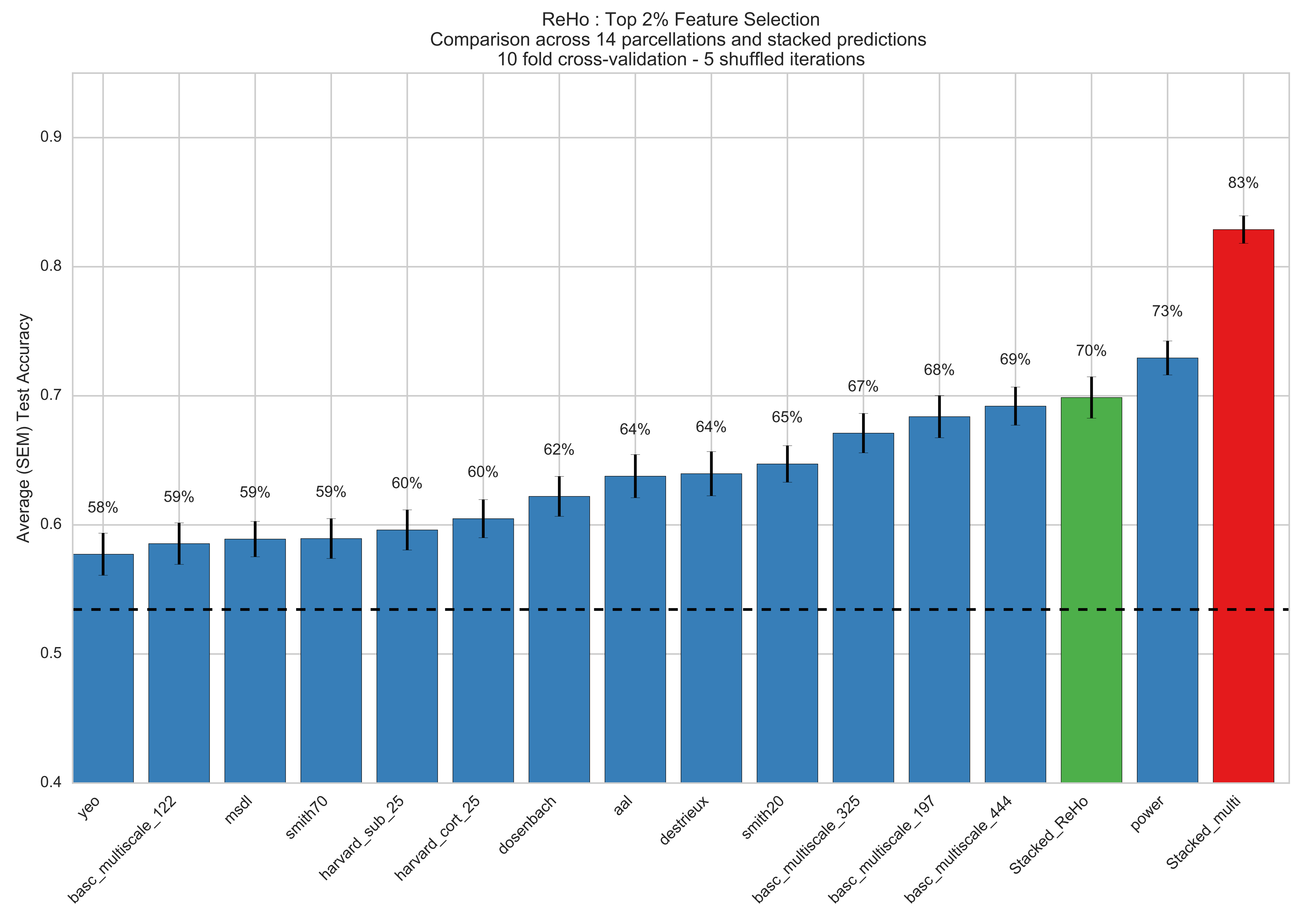

ReHo : Top 2% Feature Selection
Comparison across 14 parcellations and stacked predictions
10 fold cross-validation - 5 shuffled iterations
Average (SEM) Test Accuracy
0.4
0.5
0.6
0.7
0.8
0.9
58%
59%
59%
59%
60%
60%
62%
64%
64%
65%
67%
68%
69%
70%
73%
83%
yeo
basc_multiscale_122
msdl
smith70
harvard_sub_25
harvard_cort_25
dosenbach
aal
destrieux
smith20
basc_multiscale_325
basc_multiscale_197
basc_multiscale_444
Stacked_ReHo
power
Stacked_multi

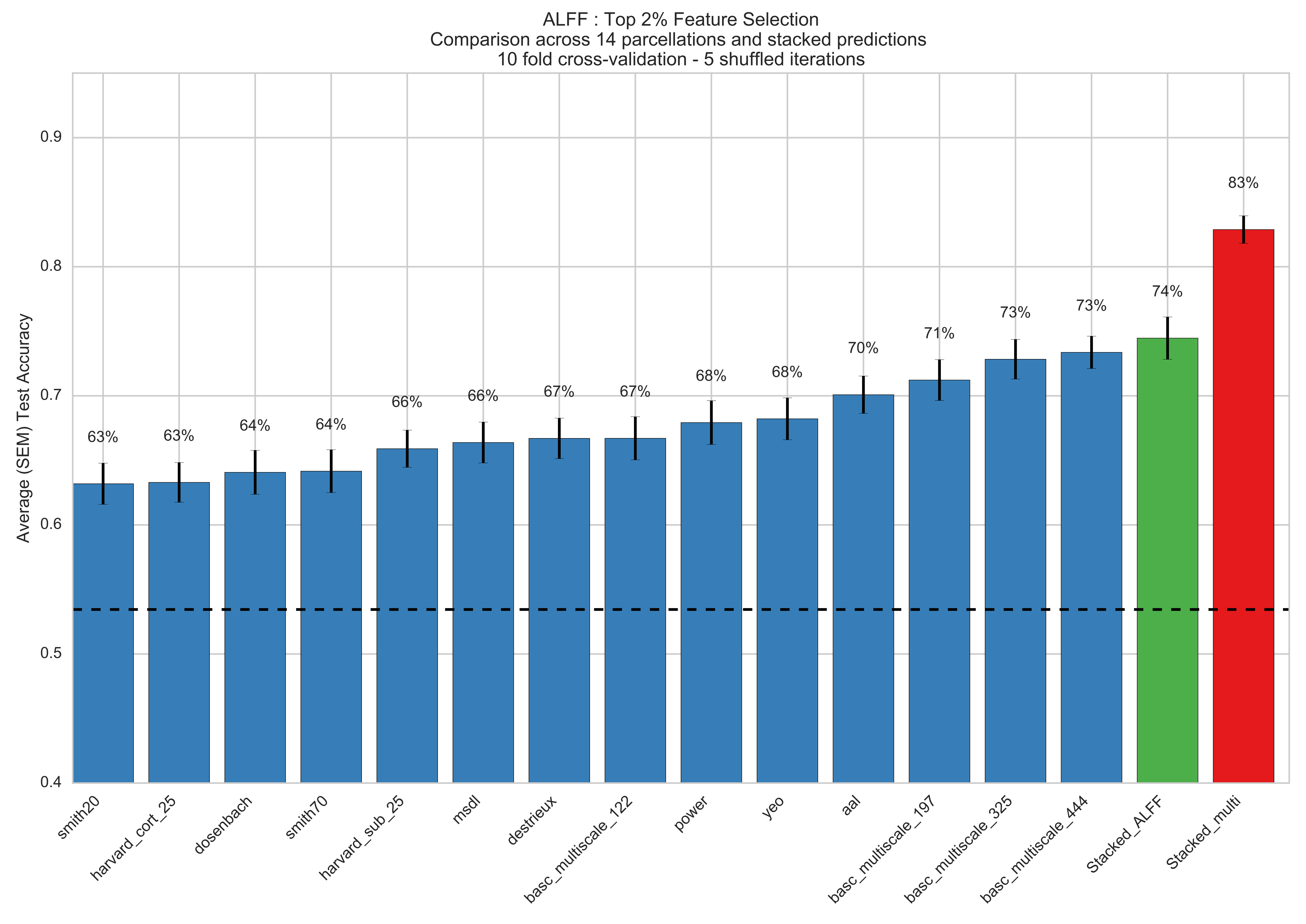

ALFF : Top 2% Feature Selection
Comparison across 14 parcellations and stacked predictions
10 fold cross-validation - 5 shuffled iterations
Average (SEM) Test Accuracy
0.4
0.5
0.6
0.7
0.8
0.9
63%
63%
64%
64%
66%
66%
67%
67%
68%
68%
70%
71%
73%
73%
74%
83%
smith20
harvard_cort_25
dosenbach
smith70
harvard_sub_25
msdl
destrieux
basc_multiscale_122
power
yeo
aal
basc_multiscale_197
basc_multiscale_325
basc_multiscale_444
Stacked_ALFF
Stacked_multi

Feature selection / reduction method :Top 5% Feature Selection

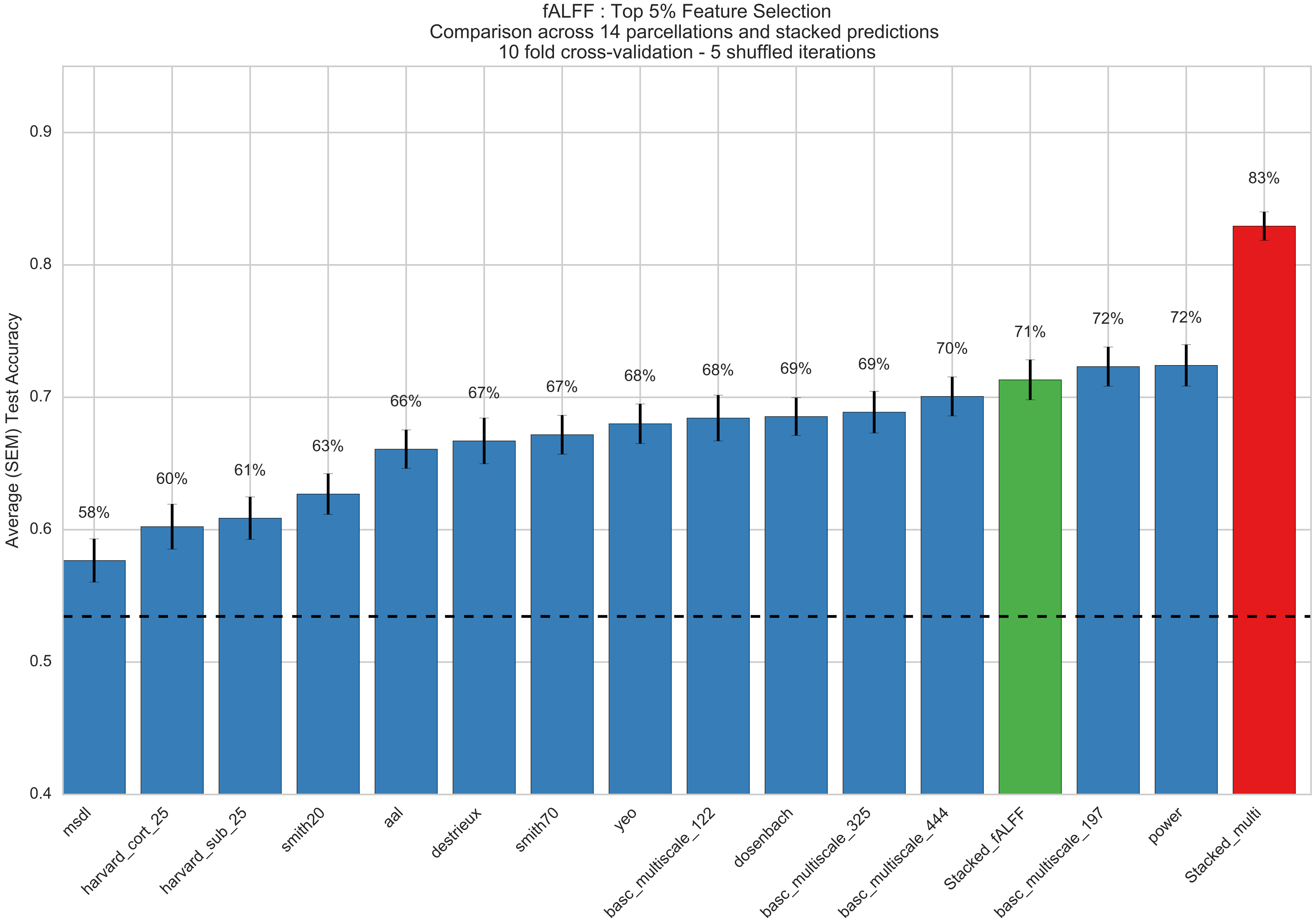
fALFF : Top 5% Feature Selection
Comparison across 14 parcellations and stacked predictions
10 fold cross-validation - 5 shuffled iterations
Average (SEM) Test Accuracy
0.4
0.5
0.6
0.7
0.8
0.9
58%
60%
61%
63%
66%
67%
67%
68%
68%
69%
69%
70%
71%
72%
72%
83%
msdl
harvard_cort_25
harvard_sub_25
smith20
aal
destrieux
smith70
yeo
basc_multiscale_122
dosenbach
basc_multiscale_325
basc_multiscale_444
Stacked_fALFF
basc_multiscale_197
power
Stacked_multi

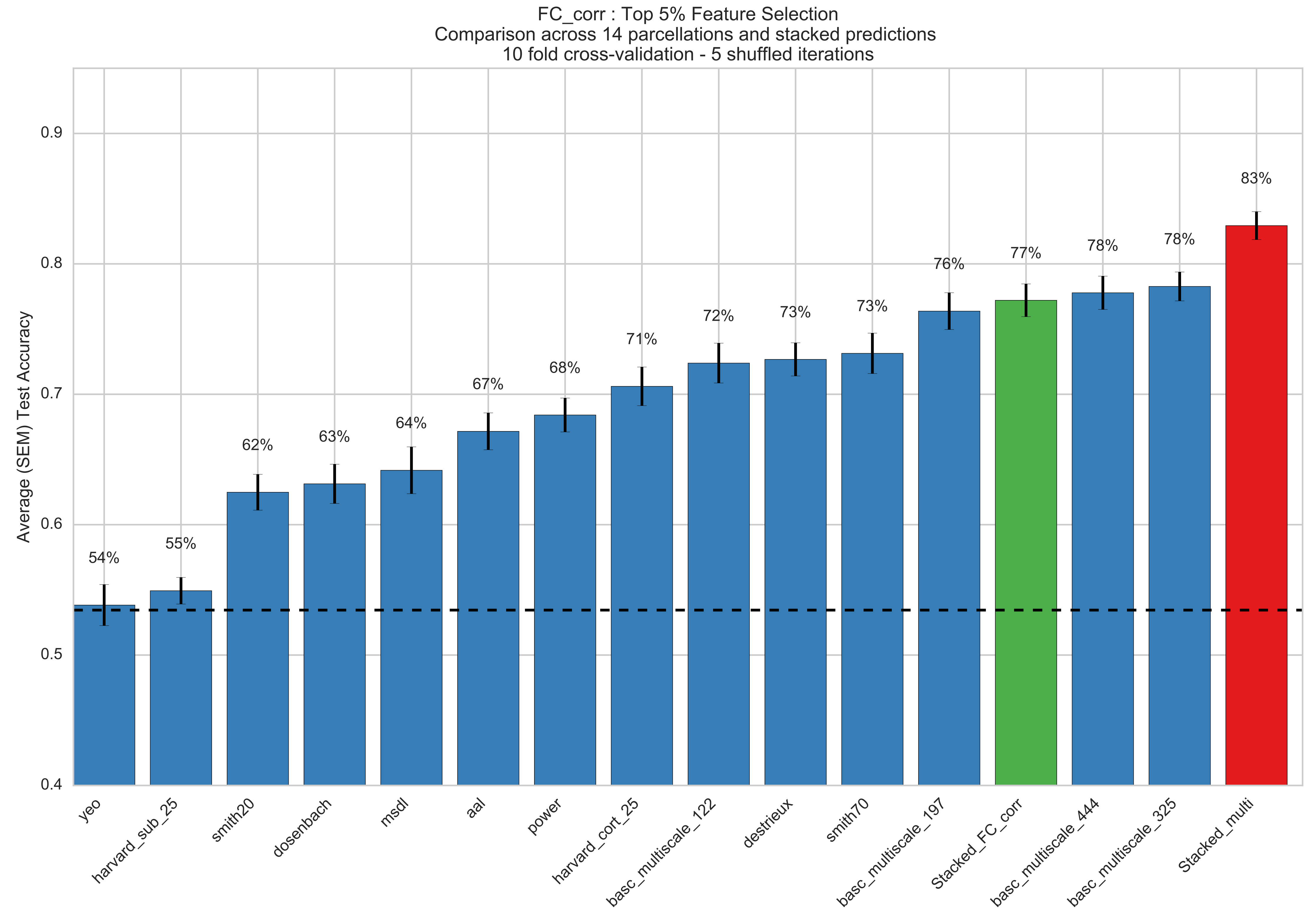
FC_corr : Top 5% Feature Selection
Comparison across 14 parcellations and stacked predictions
10 fold cross-validation - 5 shuffled iterations
Average (SEM) Test Accuracy
0.4
0.5
0.6
0.7
0.8
0.9
54%
55%
62%
63%
64%
67%
68%
71%
72%
73%
73%
76%
77%
78%
78%
83%
yeo
harvard_sub_25
smith20
dosenbach
msdl
aal
power
harvard_cort_25
basc_multiscale_122
destrieux
smith70
basc_multiscale_197
Stacked_FC_corr
basc_multiscale_444
basc_multiscale_325
Stacked_multi

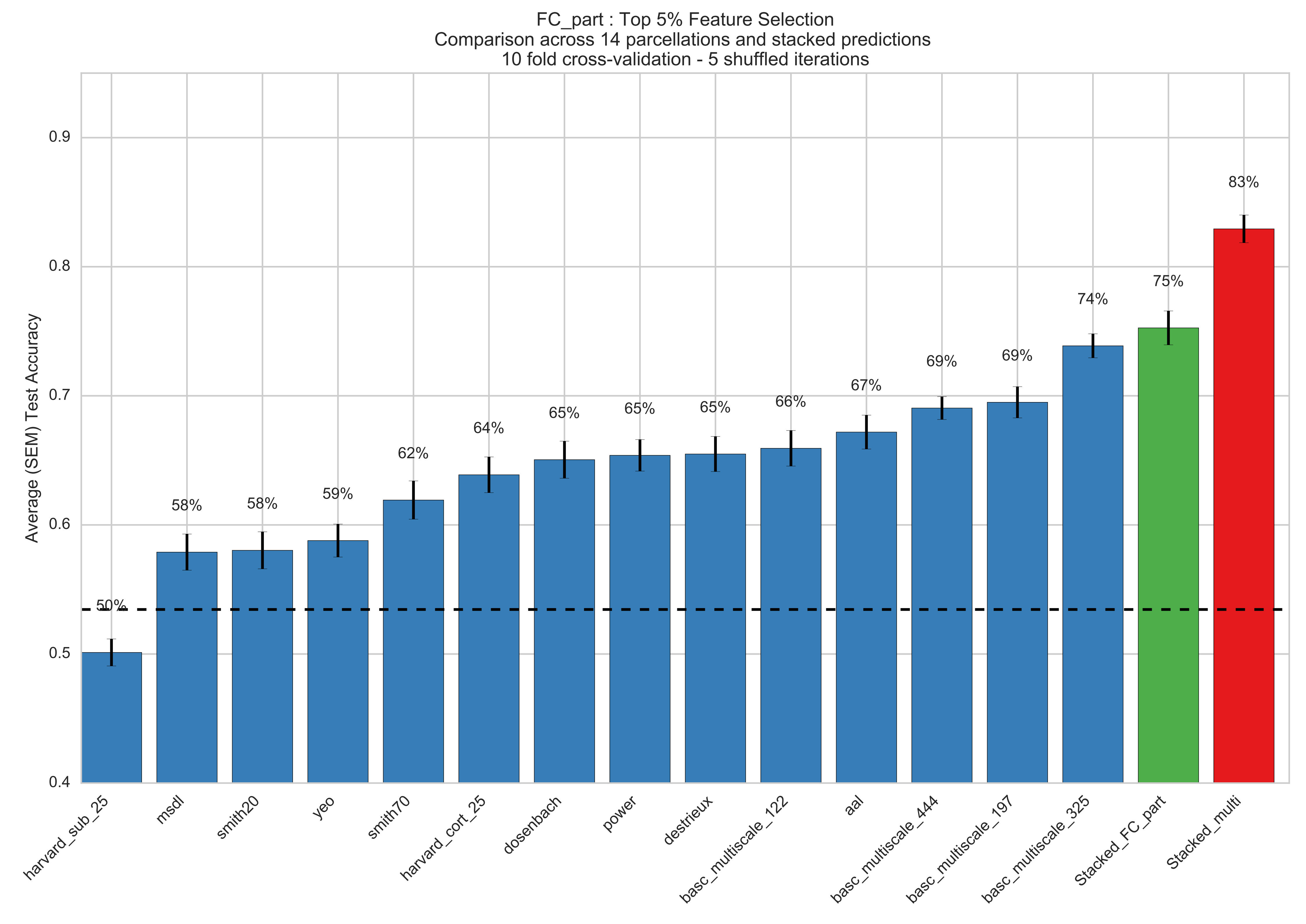

FC_part : Top 5% Feature Selection
Comparison across 14 parcellations and stacked predictions
10 fold cross-validation - 5 shuffled iterations
Average (SEM) Test Accuracy
0.4
0.5
0.6
0.7
0.8
0.9
50%
58%
58%
59%
62%
64%
65%
65%
65%
66%
67%
69%
69%
74%
75%
83%
harvard_sub_25
msdl
smith20
yeo
smith70
harvard_cort_25
dosenbach
power
destrieux
basc_multiscale_122
aal
basc_multiscale_444
basc_multiscale_197
basc_multiscale_325
Stacked_FC_part
Stacked_multi

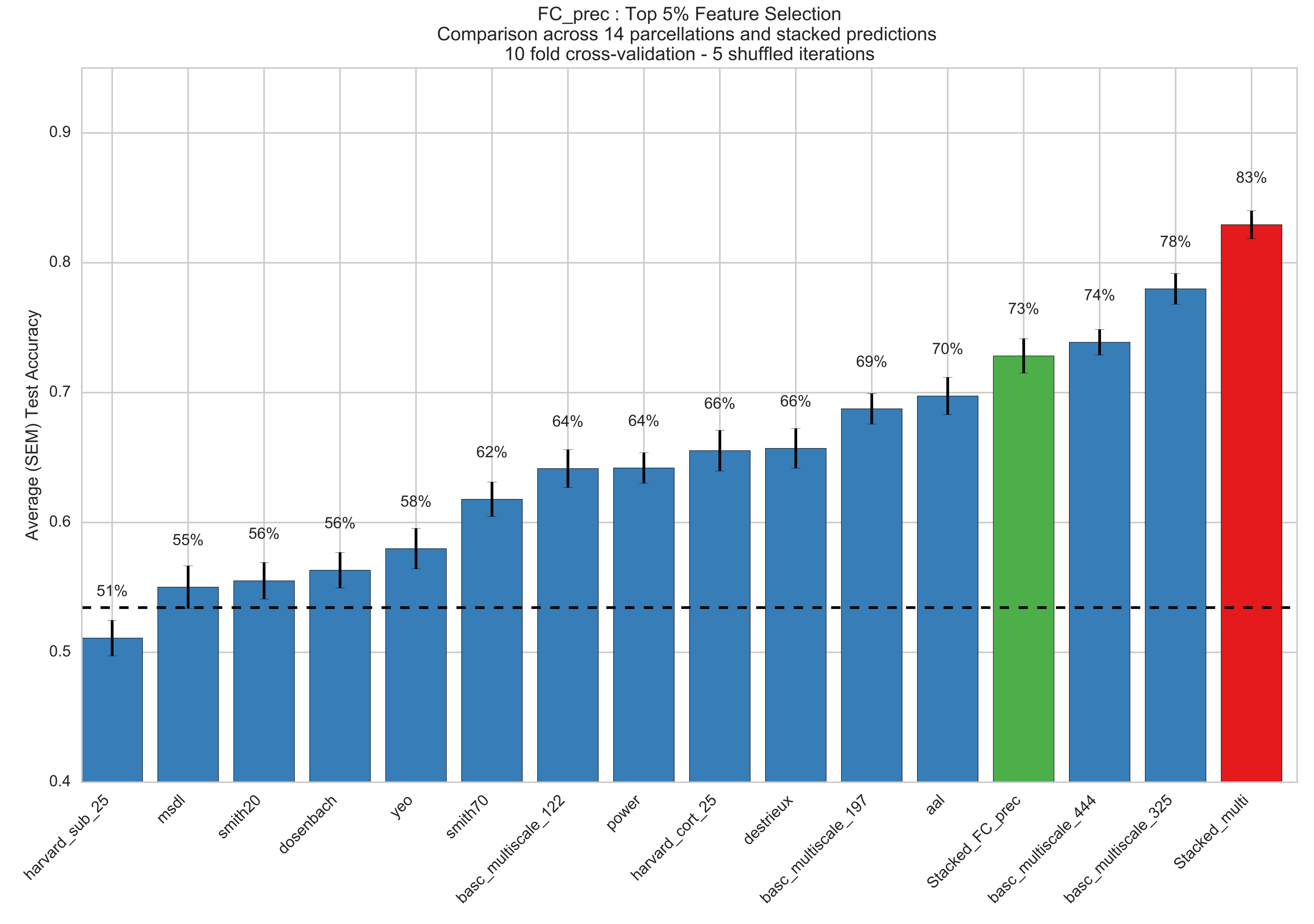

FC_prec : Top 5% Feature Selection
Comparison across 14 parcellations and stacked predictions
10 fold cross-validation - 5 shuffled iterations
Average (SEM) Test Accuracy
0.4
0.5
0.6
0.7
0.8
0.9
51%
55%
56%
56%
58%
62%
64%
64%
66%
66%
69%
70%
73%
74%
78%
83%
harvard_sub_25
msdl
smith20
dosenbach
yeo
smith70
basc_multiscale_122
power
harvard_cort_25
destrieux
basc_multiscale_197
aal
Stacked_FC_prec
basc_multiscale_444
basc_multiscale_325
Stacked_multi

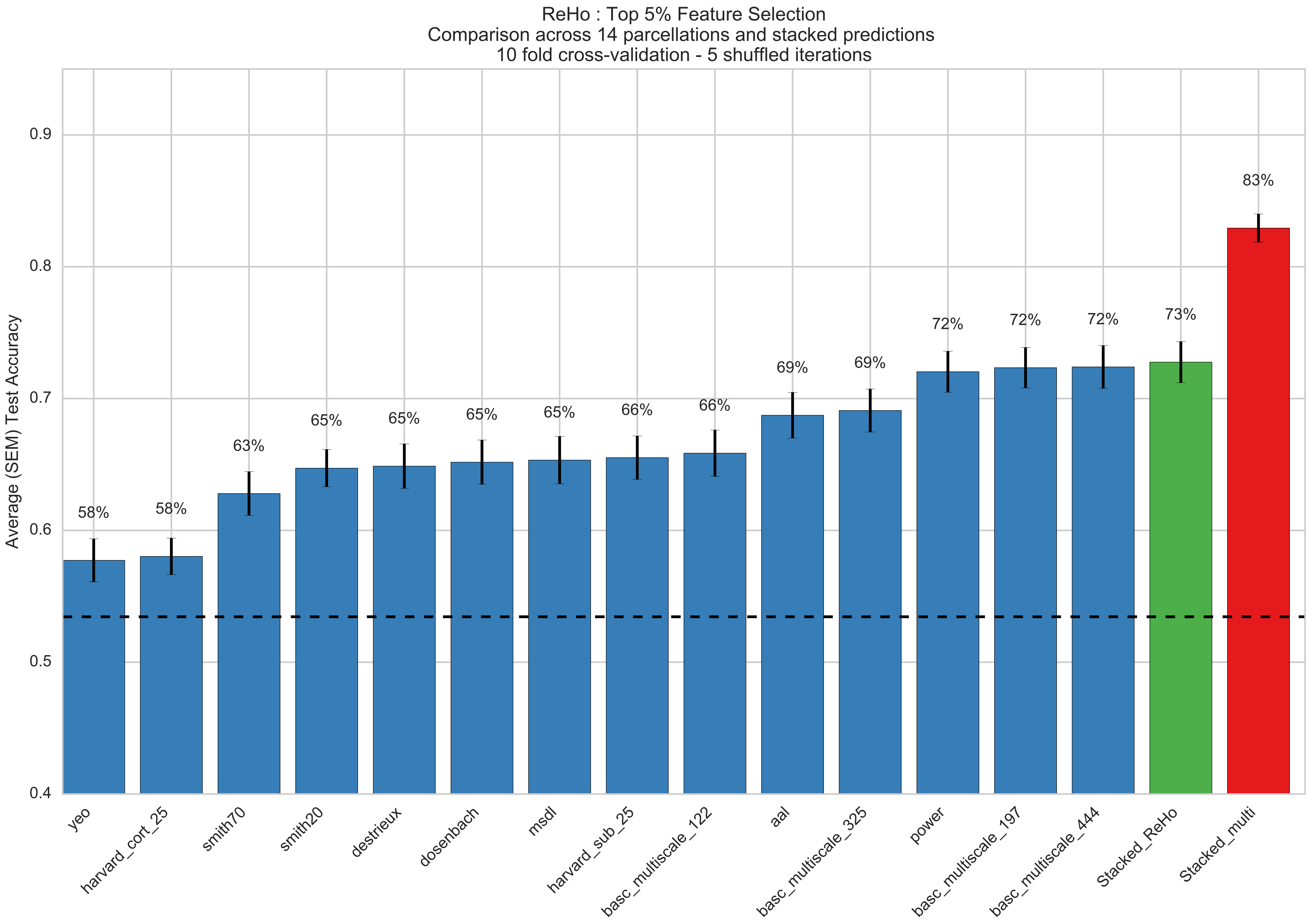
ReHo : Top 5% Feature Selection
Comparison across 14 parcellations and stacked predictions
10 fold cross-validation - 5 shuffled iterations
Average (SEM) Test Accuracy
0.9
0.8
0.7
0.6
0.5
0.4
58%
58%
63%
65%
65%
65%
65%
66%
66%
69%
69%
72%
72%
72%
73%
83%
yeo
harvard_cort_25
smith70
smith20
destrieux
dosenbach
msdl
harvard_sub_25
basc_multiscale_122
aal
basc_multiscale_325
power
basc_multiscale_197
basc_multiscale_444
Stacked_ReHo
Stacked_multi

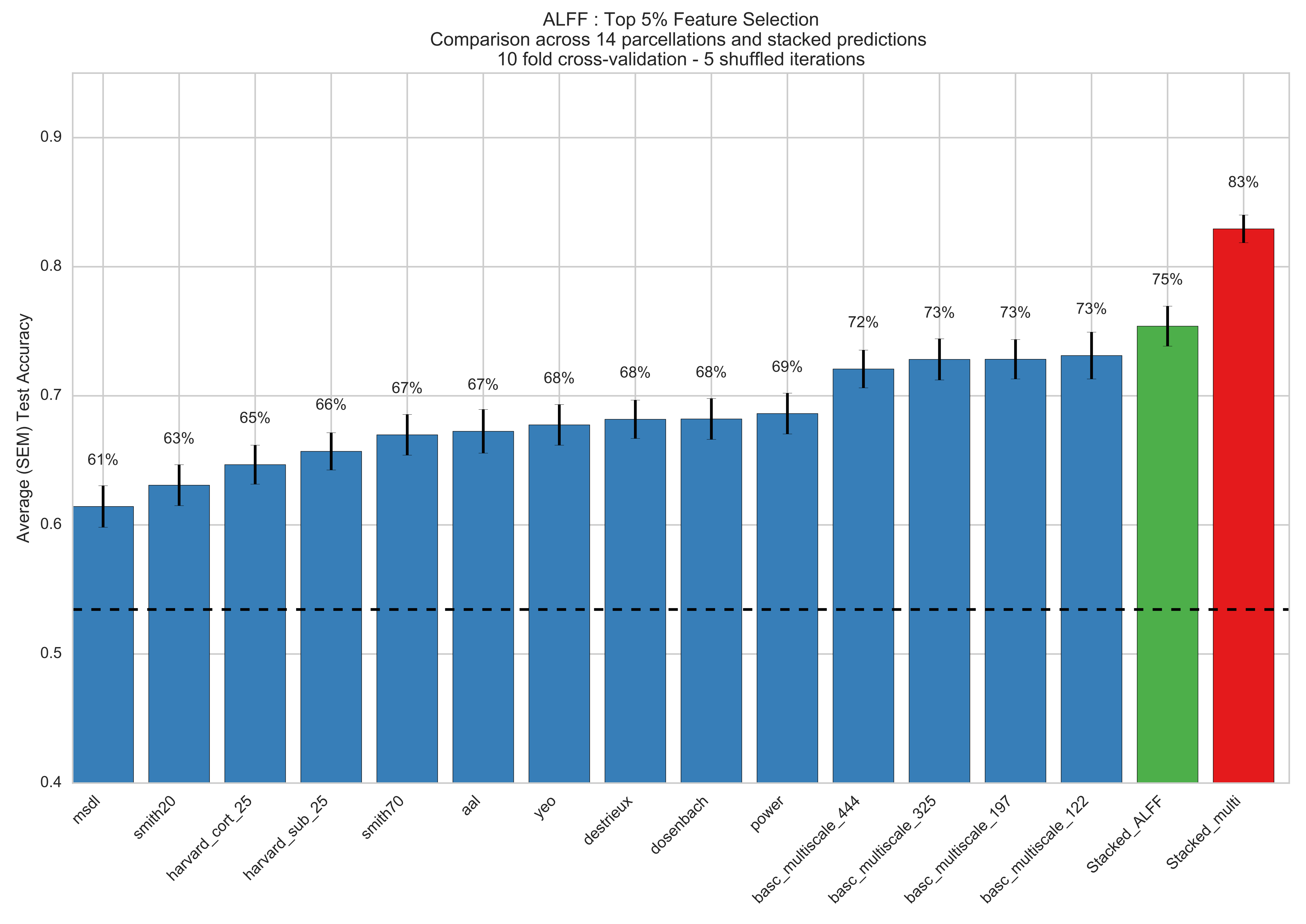
ALFF : Top 5% Feature Selection
Comparison across 14 parcellations and stacked predictions
10 fold cross-validation - 5 shuffled iterations
Average (SEM) Test Accuracy
0.4
0.5
0.6
0.7
0.8
0.9
61%
63%
65%
66%
67%
67%
68%
68%
68%
69%
72%
73%
73%
73%
75%
83%
msdl
smith20
harvard_cort_25
harvard_sub_25
smith70
aal
yeo
destrieux
dosenbach
power
basc_multiscale_444
basc_multiscale_325
basc_multiscale_197
basc_multiscale_122
Stacked_ALFF
Stacked_multi

Feature selection / reduction method :Top 10% Feature Selection

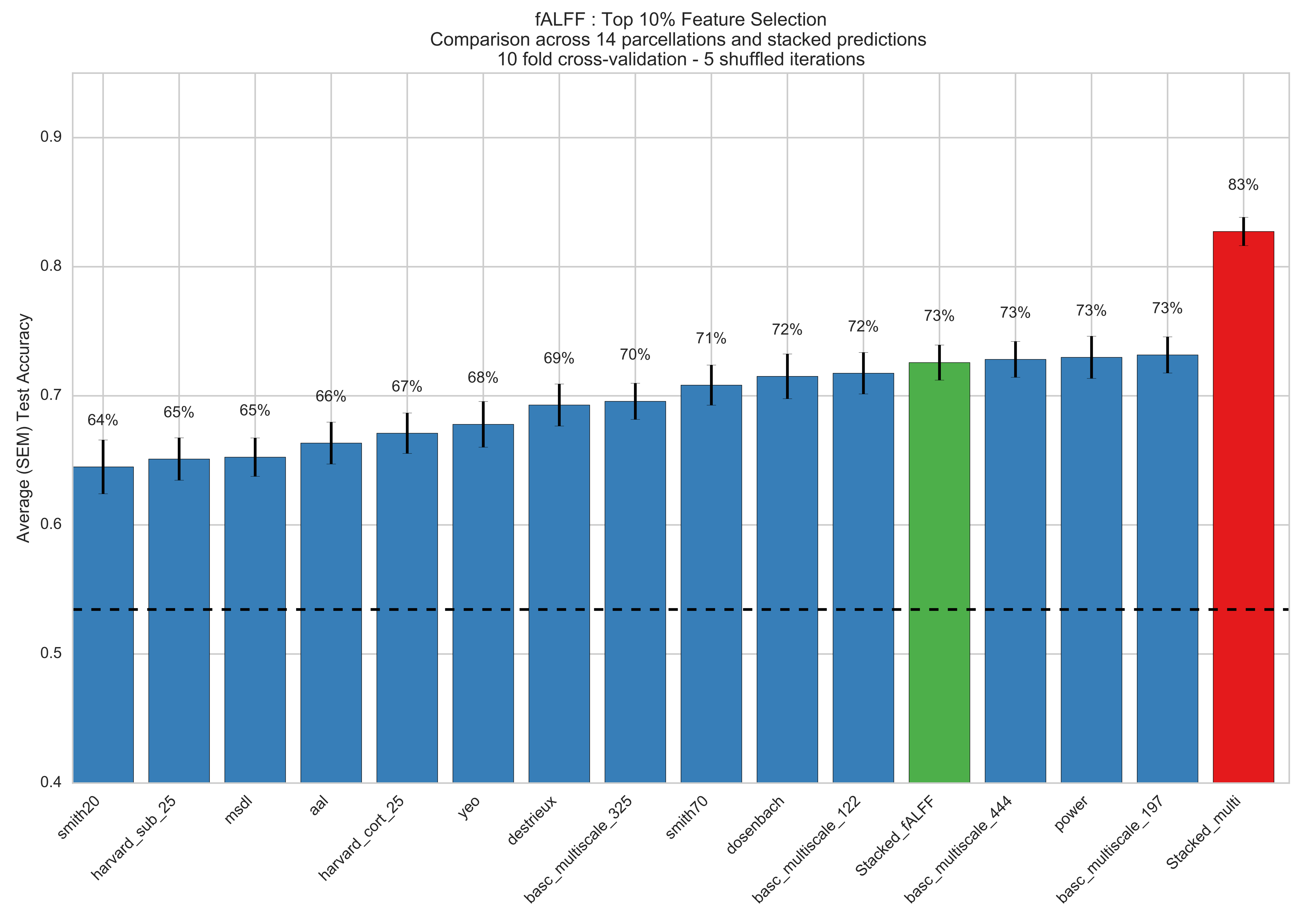
fALFF : Top 10% Feature Selection
Comparison across 14 parcellations and stacked predictions
10 fold cross-validation - 5 shuffled iterations
Average (SEM) Test Accuracy
0.9
0.8
0.7
0.6
0.5
0.4
64%
65%
65%
66%
67%
68%
69%
70%
71%
72%
72%
73%
73%
73%
73%
83%
smith20
harvard_sub_25
msdl
aal
harvard_cort_25
yeo
destrieux
basc_multiscale_325
smith70
dosenbach
basc_multiscale_122
Stacked_fALFF
basc_multiscale_444
power
basc_multiscale_197
Stacked_multi

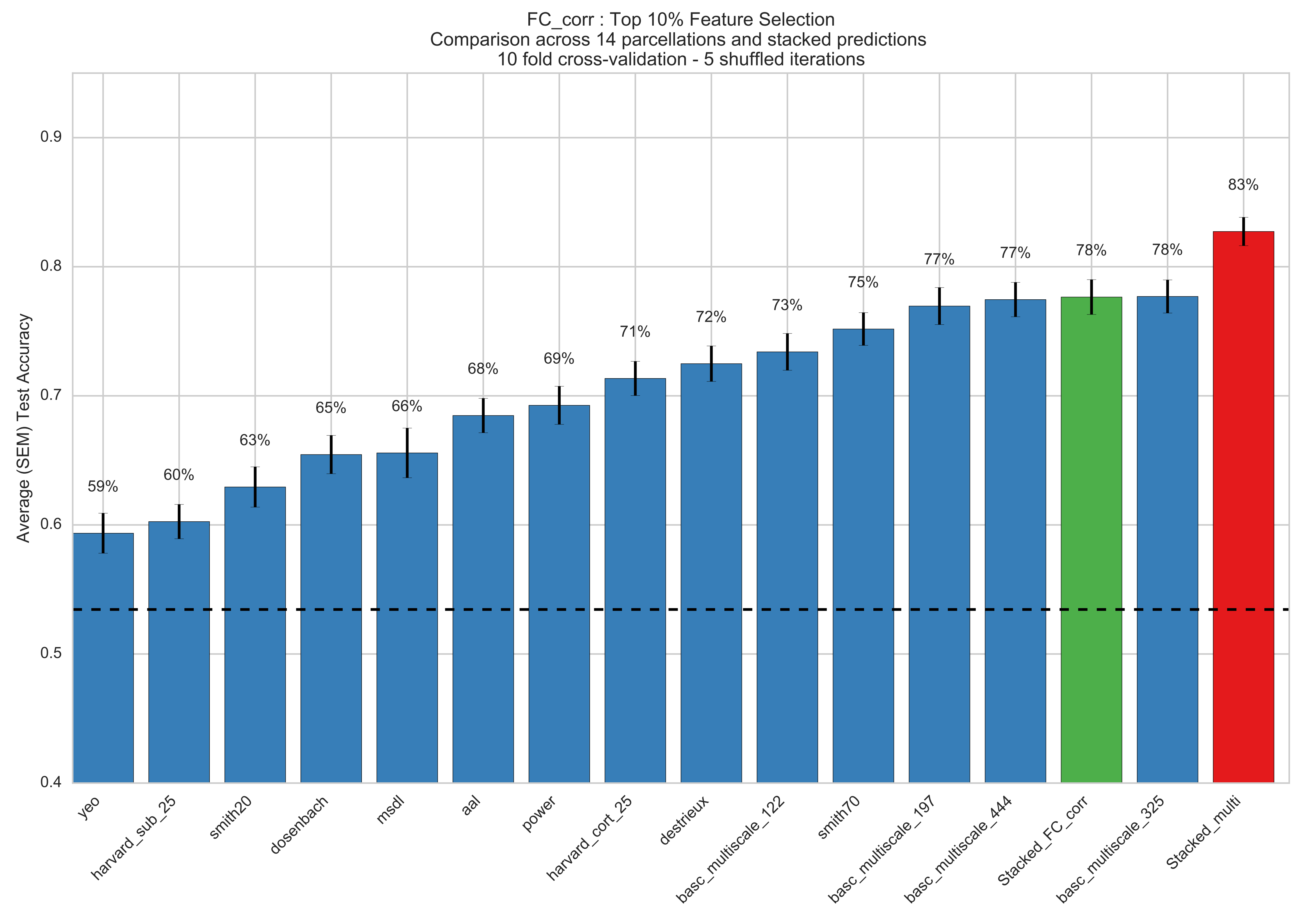

FC_corr : Top 10% Feature Selection
Comparison across 14 parcellations and stacked predictions
10 fold cross-validation - 5 shuffled iterations
Average (SEM) Test Accuracy
0.4
0.5
0.6
0.7
0.8
0.9
59%
60%
63%
65%
66%
68%
69%
71%
72%
73%
75%
77%
77%
78%
78%
83%
yeo
harvard_sub_25
smith20
dosenbach
msdl
aal
power
harvard_cort_25
destrieux
basc_multiscale_122
smith70
basc_multiscale_197
basc_multiscale_444
Stacked_FC_corr
basc_multiscale_325
Stacked_multi

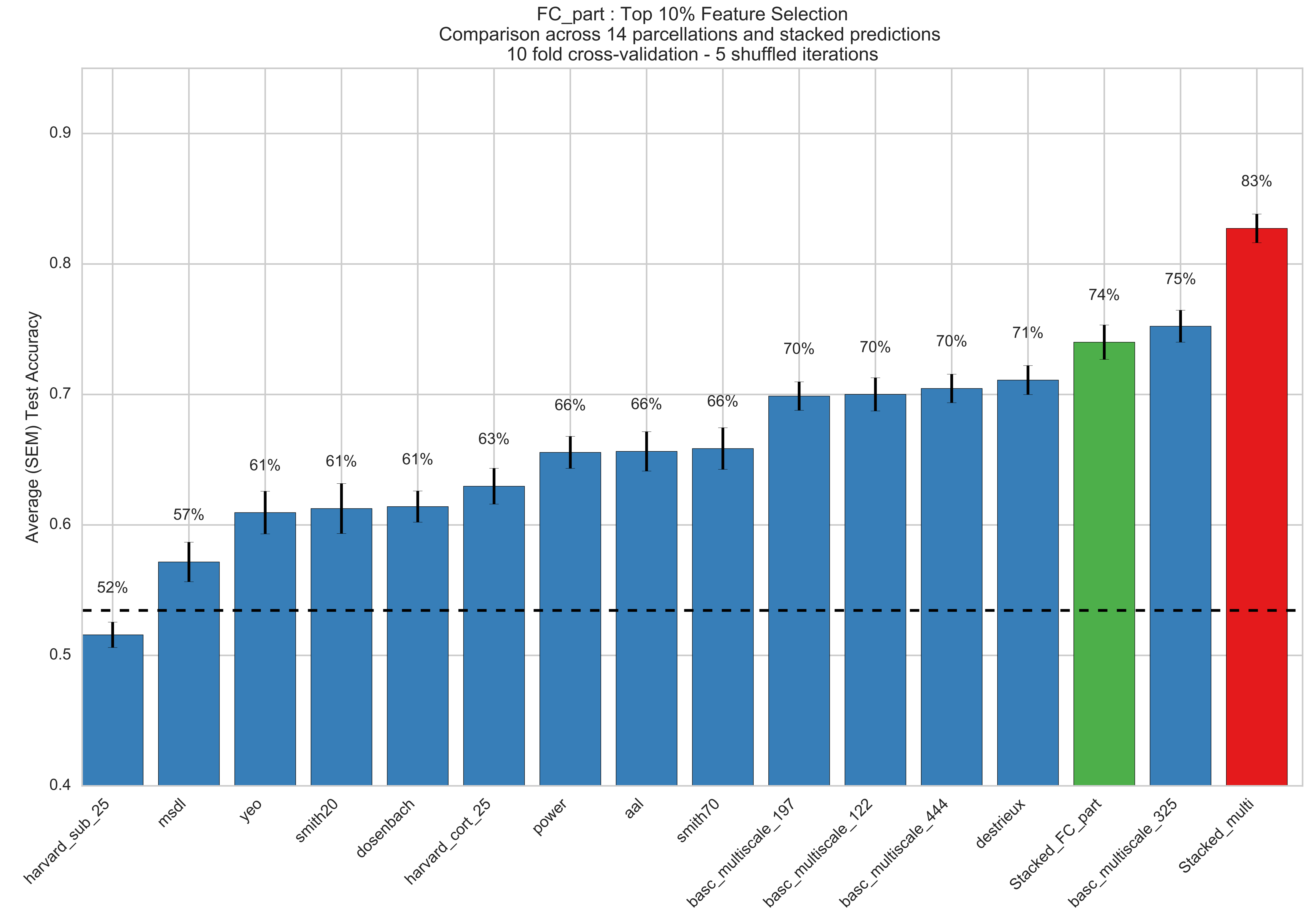
FC_part : Top 10% Feature Selection
Comparison across 14 parcellations and stacked predictions
10 fold cross-validation - 5 shuffled iterations
Average (SEM) Test Accuracy
0.4
0.5
0.6
0.7
0.8
0.9
52%
57%
61%
61%
61%
63%
66%
66%
66%
70%
70%
70%
71%
74%
75%
83%
harvard_sub_25
msdl
yeo
smith20
dosenbach
harvard_cort_25
power
aal
smith70
basc_multiscale_197
basc_multiscale_122
basc_multiscale_444
destrieux
Stacked_FC_part
basc_multiscale_325
Stacked_multi

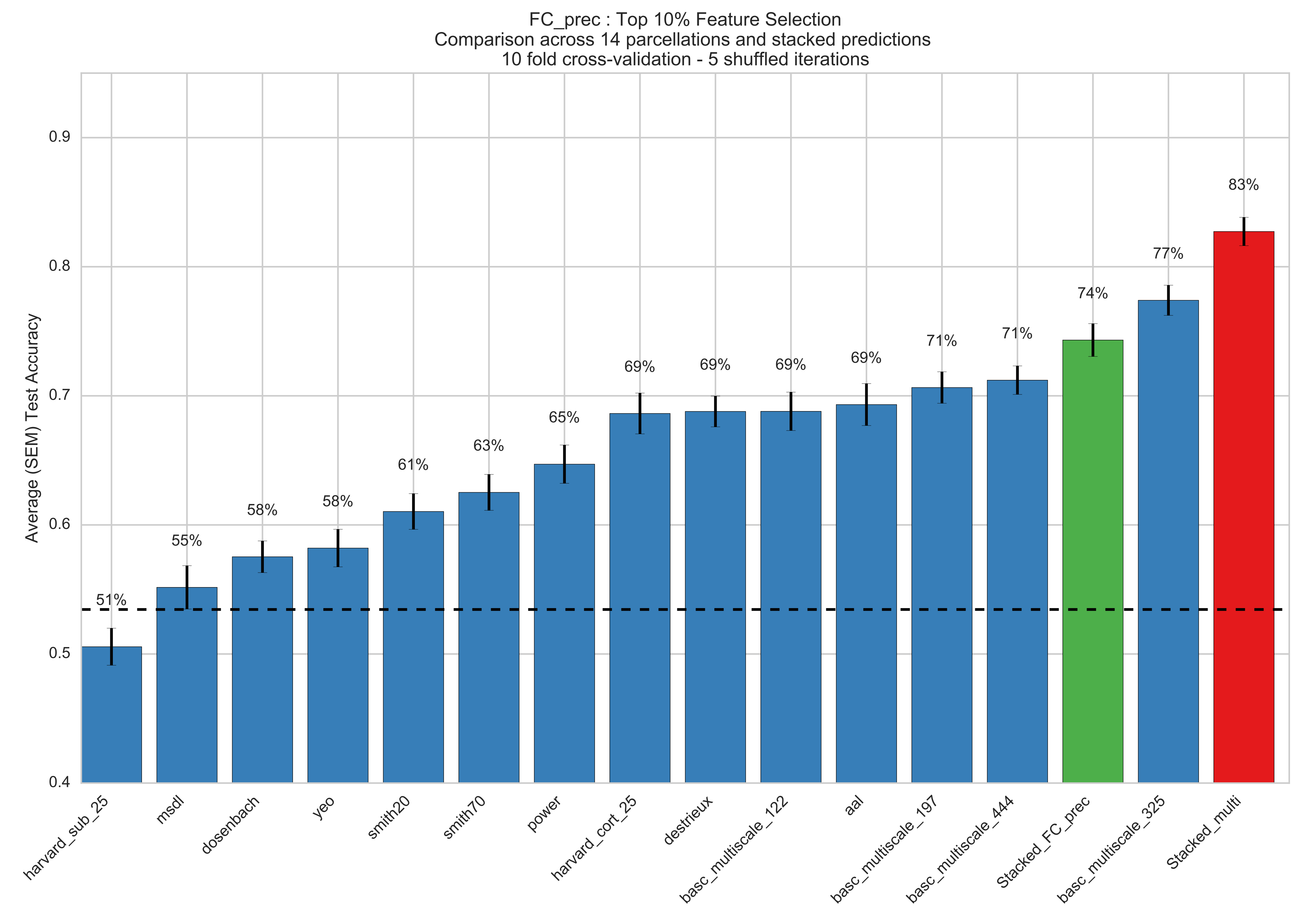

FC_prec : Top 10% Feature Selection
Comparison across 14 parcellations and stacked predictions
10 fold cross-validation - 5 shuffled iterations
Average (SEM) Test Accuracy
0.4
0.5
0.6
0.7
0.8
0.9
51%
55%
58%
58%
61%
63%
65%
69%
69%
69%
69%
71%
71%
74%
77%
83%
harvard_sub_25
msdl
dosenbach
yeo
smith20
smith70
power
harvard_cort_25
destrieux
basc_multiscale_122
aal
basc_multiscale_197
basc_multiscale_444
Stacked_FC_prec
basc_multiscale_325
Stacked_multi

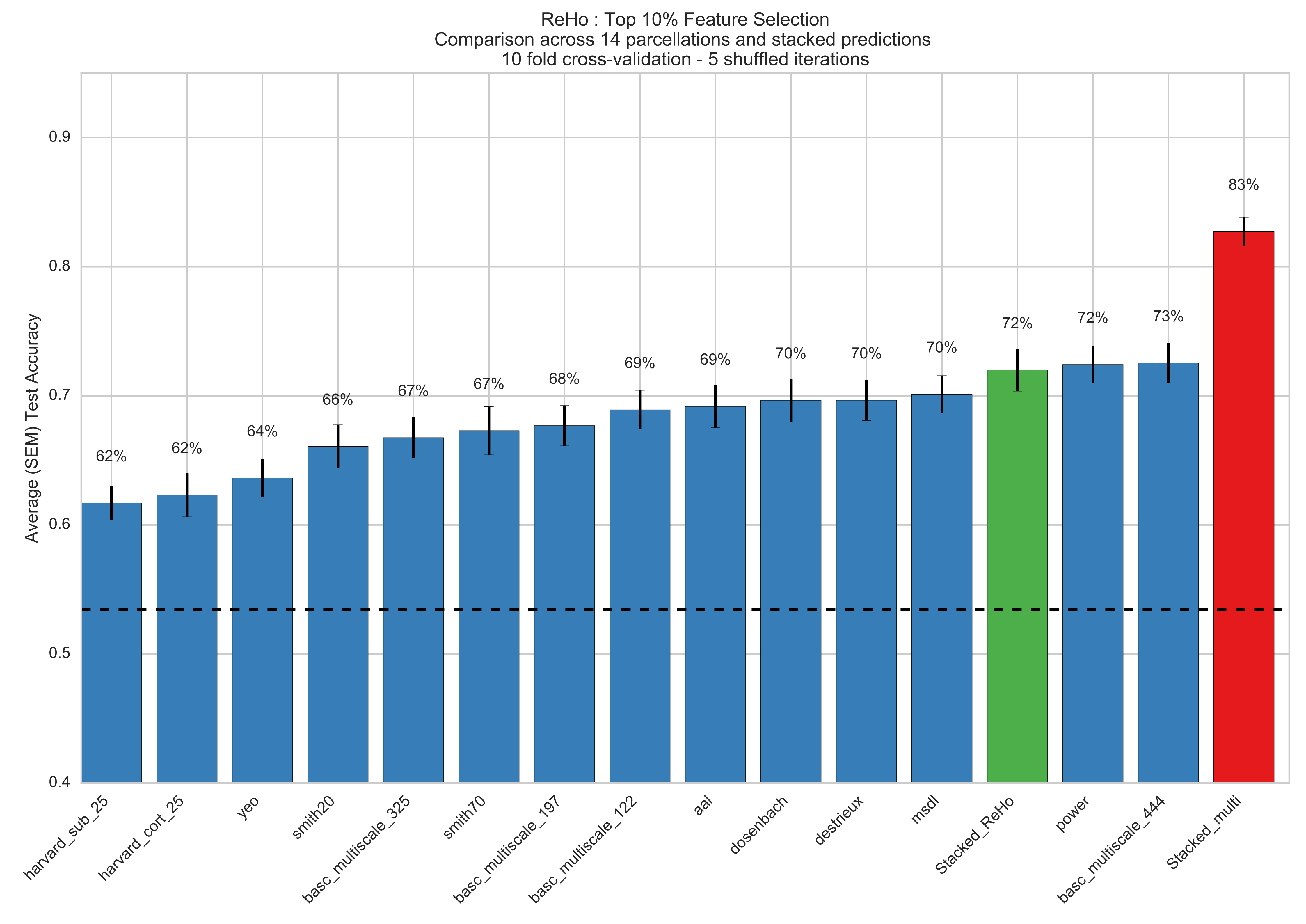
ReHo : Top 10% Feature Selection
Comparison across 14 parcellations and stacked predictions
10 fold cross-validation - 5 shuffled iterations
Average (SEM) Test Accuracy
0.9
0.8
0.7
0.6
0.5
0.4
62%
62%
64%
66%
67%
67%
68%
69%
69%
70%
70%
70%
72%
72%
73%
83%
harvard_sub_25
harvard_cort_25
yeo
smith20
basc_multiscale_325
smith70
basc_multiscale_197
basc_multiscale_122
aal
dosenbach
destrieux
msdl
Stacked_ReHo
power
basc_multiscale_444
Stacked_multi

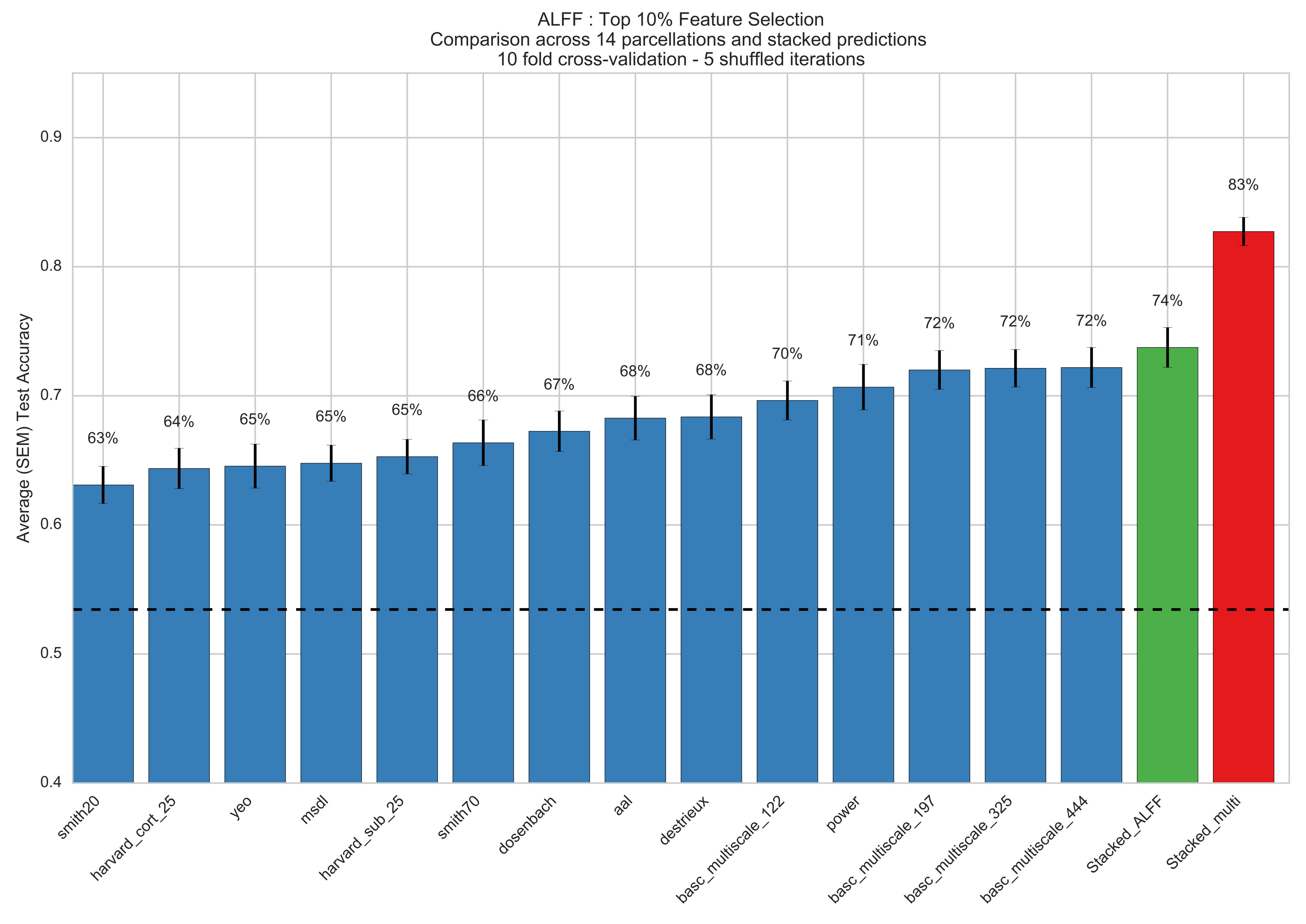
ALFF : Top 10% Feature Selection
Comparison across 14 parcellations and stacked predictions
10 fold cross-validation - 5 shuffled iterations
Average (SEM) Test Accuracy
0.4
0.5
0.6
0.7
0.8
0.9
63%
64%
65%
65%
65%
66%
67%
68%
68%
70%
71%
72%
72%
72%
74%
83%
smith20
harvard_cort_25
yeo
msdl
harvard_sub_25
smith70
dosenbach
aal
destrieux
basc_multiscale_122
power
basc_multiscale_197
basc_multiscale_325
basc_multiscale_444
Stacked_ALFF
Stacked_multi

Feature selection / reduction method :Top 0.5% Feature Selection

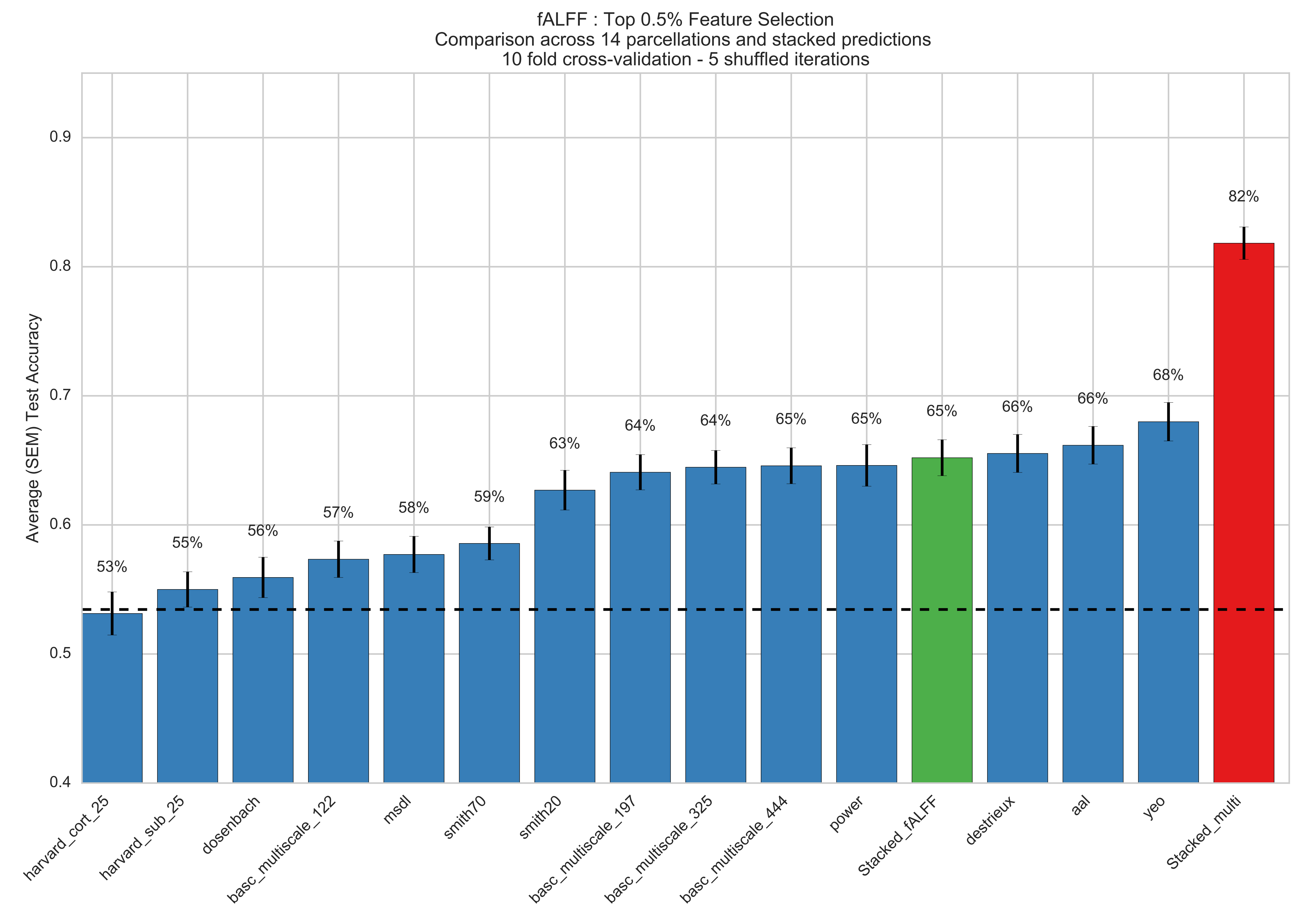
fALFF : Top 0.5% Feature Selection
Comparison across 14 parcellations and stacked predictions
10 fold cross-validation - 5 shuffled iterations
Average (SEM) Test Accuracy
0.9
0.8
0.7
0.6
0.5
0.4
53%
55%
56%
57%
58%
59%
63%
64%
64%
65%
65%
65%
66%
66%
68%
82%
harvard_cort_25
harvard_sub_25
dosenbach
basc_multiscale_122
msdl
smith70
smith20
basc_multiscale_197
basc_multiscale_325
basc_multiscale_444
power
Stacked_fALFF
destrieux
aal
yeo
Stacked_multi

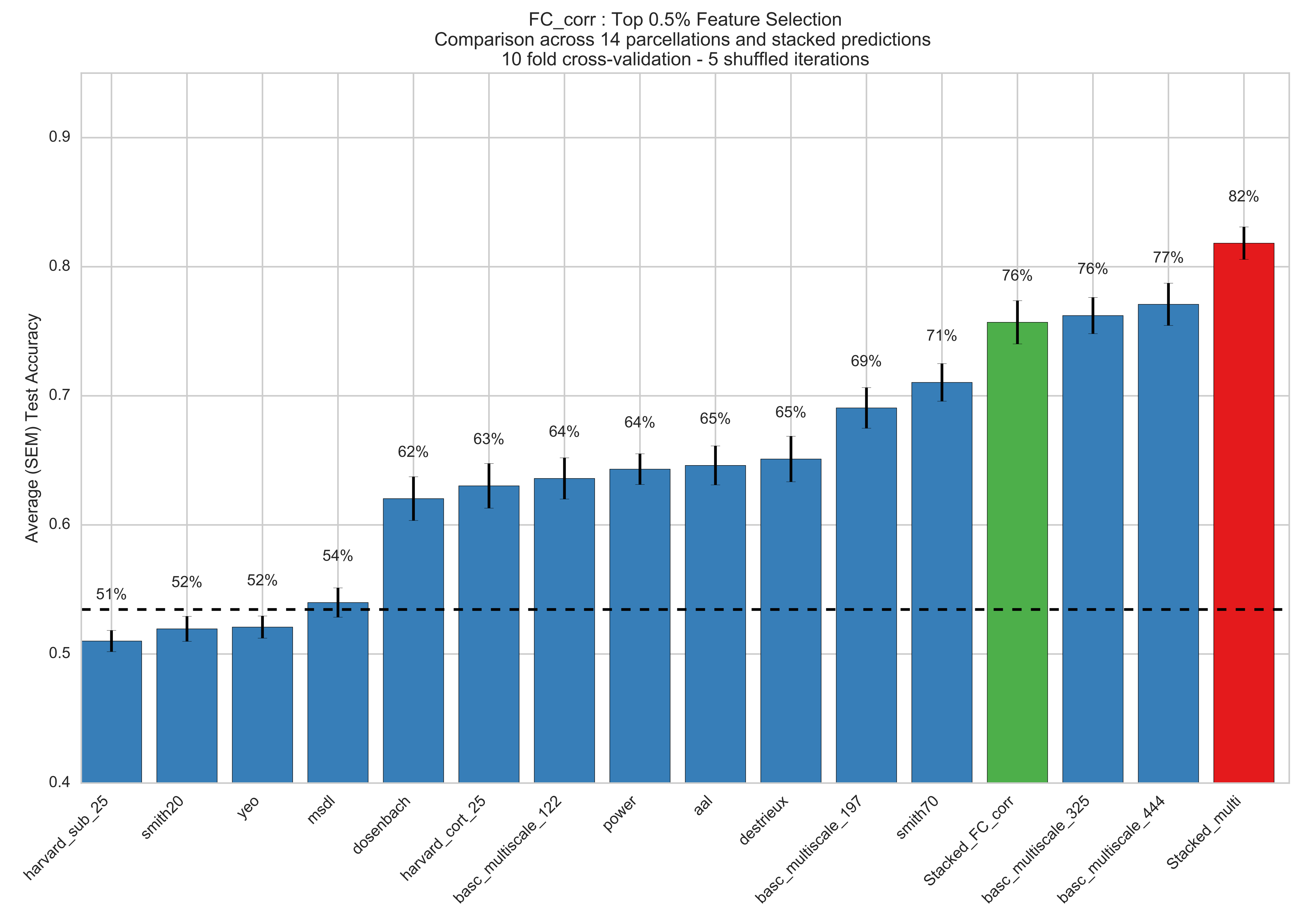

FC_corr : Top 0.5% Feature Selection
Comparison across 14 parcellations and stacked predictions
10 fold cross-validation - 5 shuffled iterations
Average (SEM) Test Accuracy
0.4
0.5
0.6
0.7
0.8
0.9
51%
52%
52%
54%
62%
63%
64%
64%
65%
65%
69%
71%
76%
76%
77%
82%
harvard_sub_25
smith20
yeo
msdl
dosenbach
harvard_cort_25
basc_multiscale_122
power
aal
destrieux
basc_multiscale_197
smith70
Stacked_FC_corr
basc_multiscale_325
basc_multiscale_444
Stacked_multi

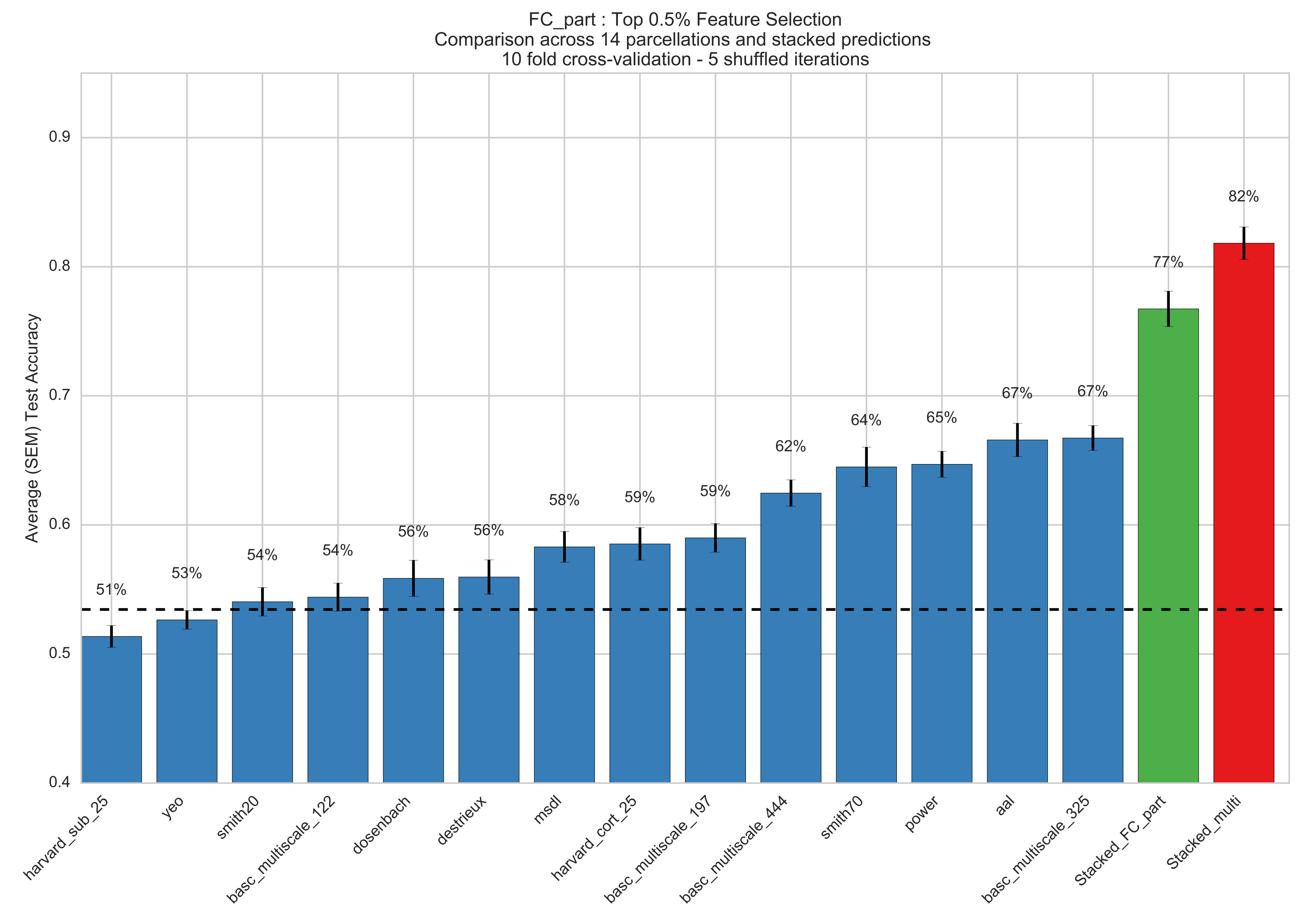
FC_part : Top 0.5% Feature Selection
Comparison across 14 parcellations and stacked predictions
10 fold cross-validation - 5 shuffled iterations
Average (SEM) Test Accuracy
0.4
0.5
0.6
0.7
0.8
0.9
51%
53%
54%
54%
56%
56%
58%
59%
59%
62%
64%
65%
67%
67%
77%
82%
harvard_sub_25
yeo
smith20
basc_multiscale_122
dosenbach
destrieux
msdl
harvard_cort_25
basc_multiscale_197
basc_multiscale_444
smith70
power
aal
basc_multiscale_325
Stacked_FC_part
Stacked_multi

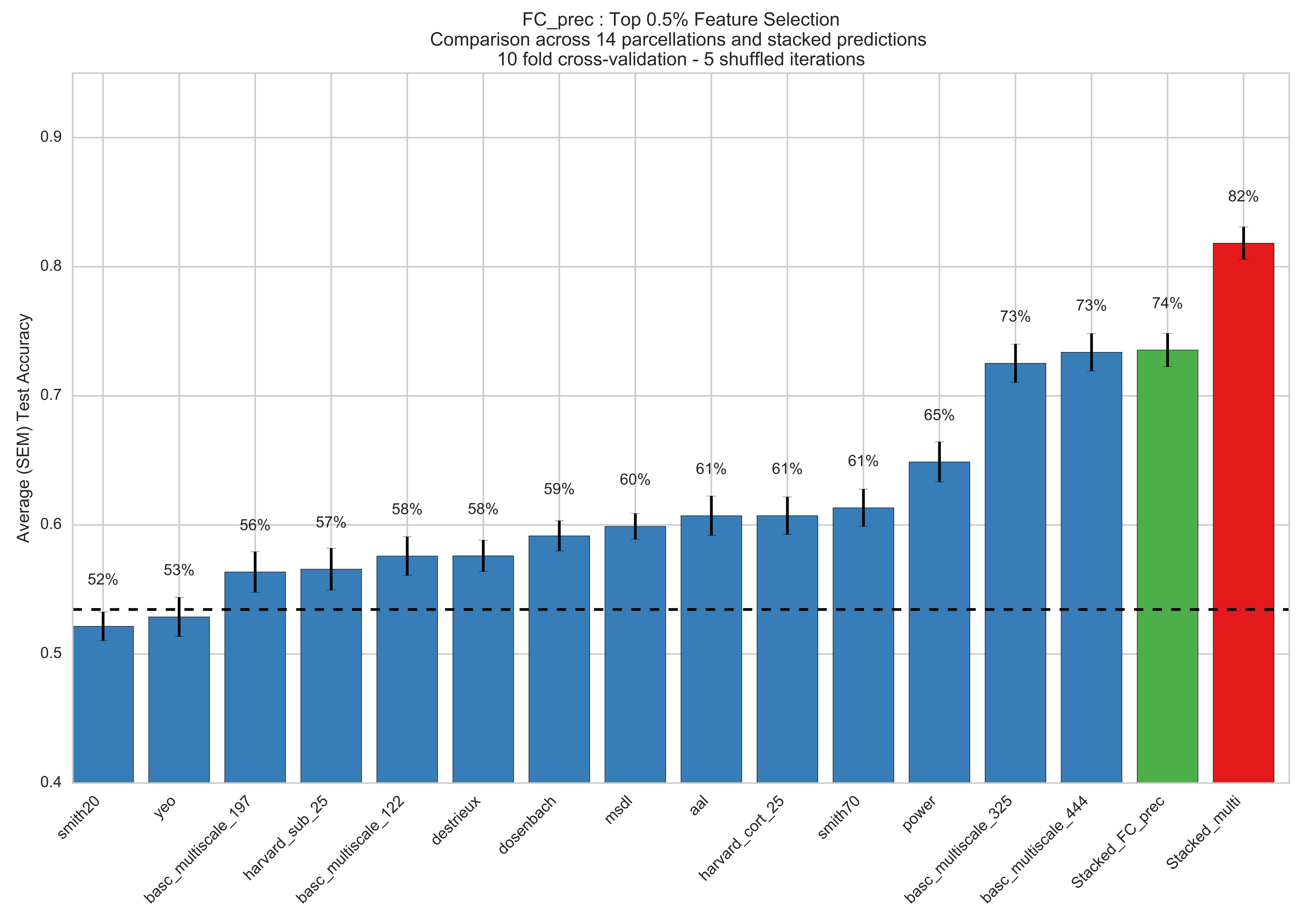

FC_prec : Top 0.5% Feature Selection
Comparison across 14 parcellations and stacked predictions
10 fold cross-validation - 5 shuffled iterations
Average (SEM) Test Accuracy
0.9
0.8
0.7
0.6
0.5
0.4
52%
53%
56%
57%
58%
58%
59%
60%
61%
61%
61%
65%
73%
73%
74%
82%
smith20
yeo
basc_multiscale_197
harvard_sub_25
basc_multiscale_122
destrieux
dosenbach
msdl
aal
harvard_cort_25
smith70
power
basc_multiscale_325
basc_multiscale_444
Stacked_FC_prec
Stacked_multi

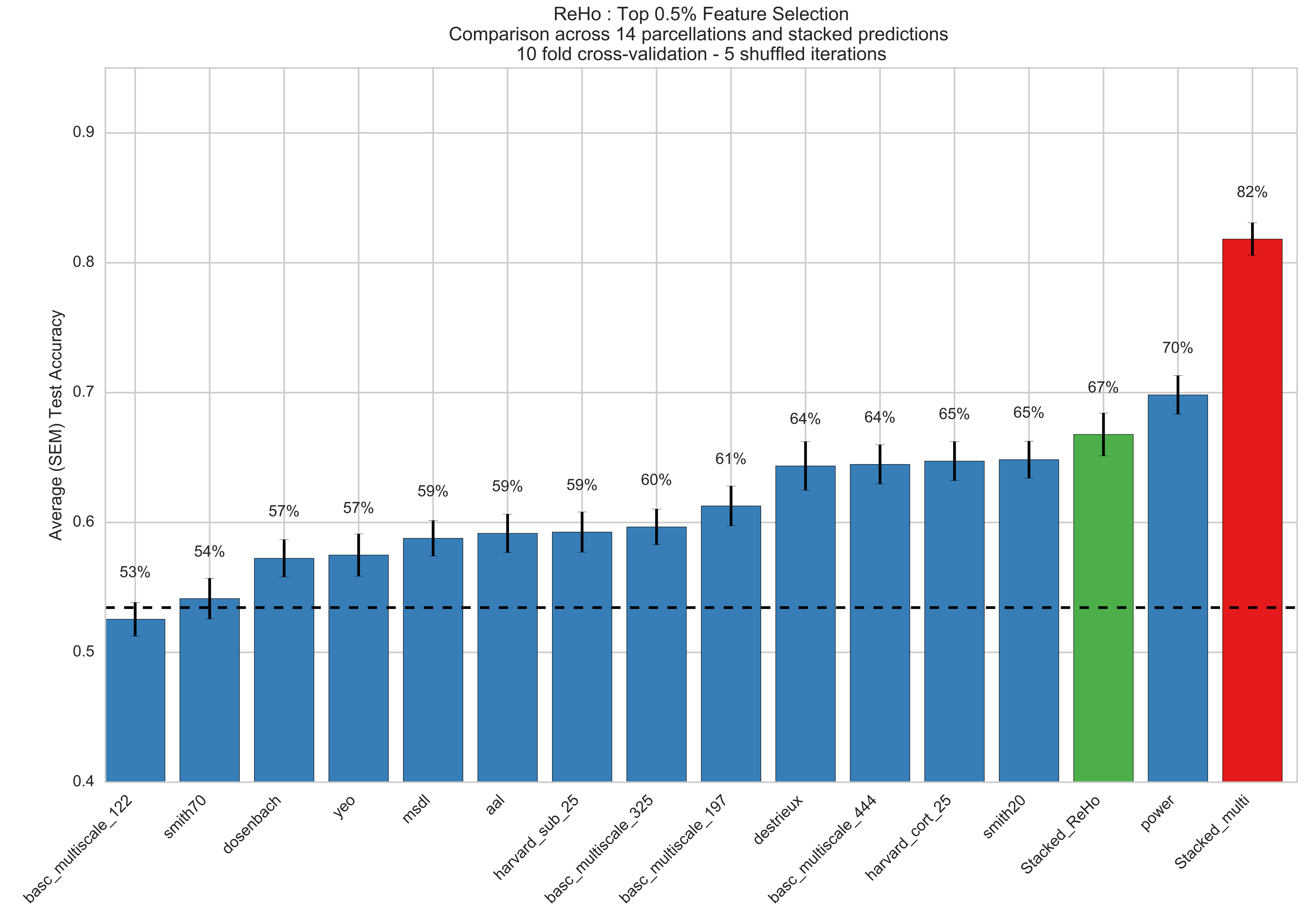

ReHo : Top 0.5% Feature Selection
Comparison across 14 parcellations and stacked predictions
10 fold cross-validation - 5 shuffled iterations
Average (SEM) Test Accuracy
0.9
0.8
0.7
0.6
0.5
0.4
53%
54%
57%
57%
59%
59%
59%
60%
61%
64%
64%
65%
65%
67%
70%
82%
basc_multiscale_122
smith70
dosenbach
yeo
msdl
aal
harvard_sub_25
basc_multiscale_325
basc_multiscale_197
destrieux
basc_multiscale_444
harvard_cort_25
smith20
Stacked_ReHo
power
Stacked_multi

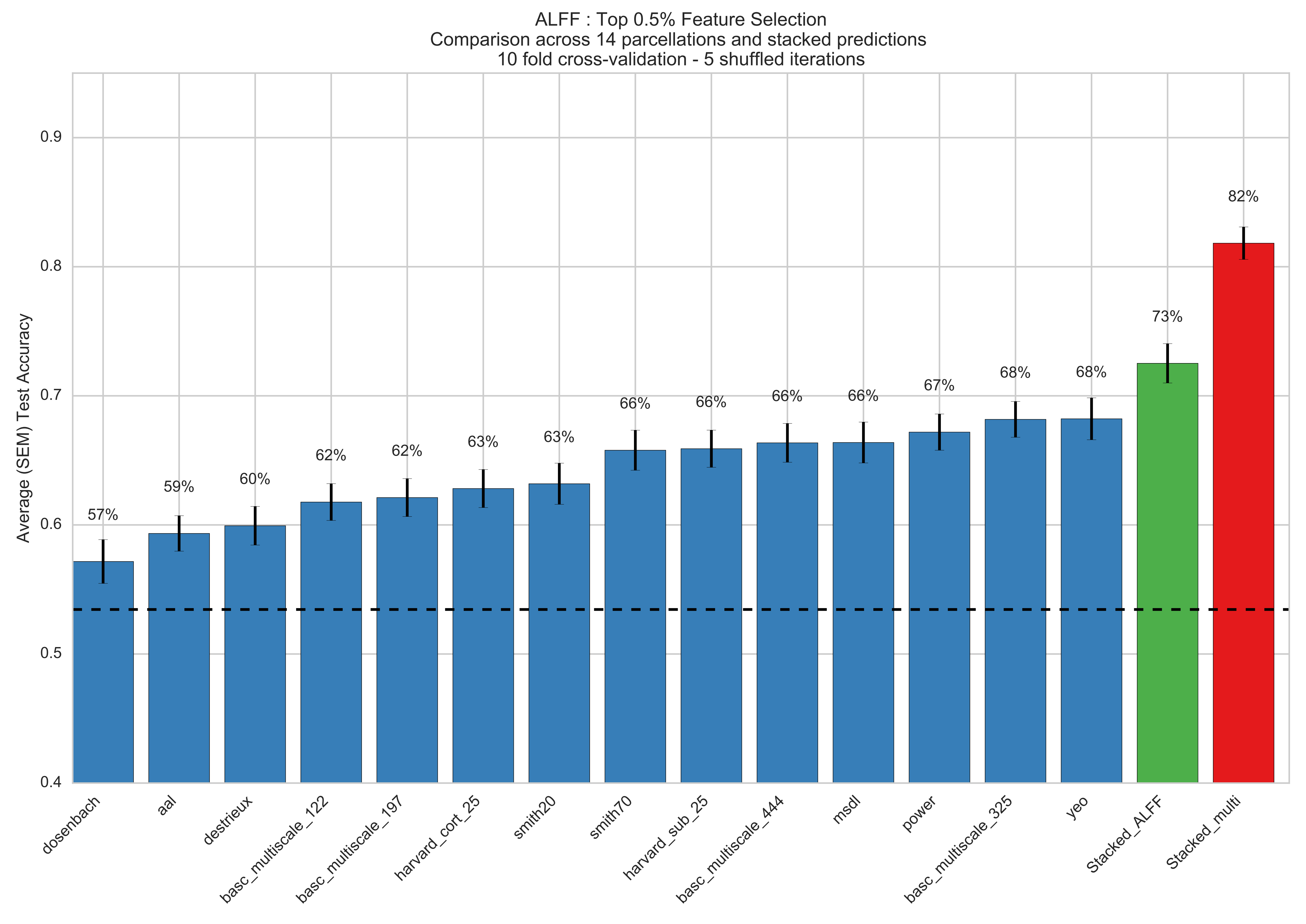
ALFF : Top 0.5% Feature Selection
Comparison across 14 parcellations and stacked predictions
10 fold cross-validation - 5 shuffled iterations
Average (SEM) Test Accuracy
0.4
0.5
0.6
0.7
0.8
0.9
57%
59%
60%
62%
62%
63%
63%
66%
66%
66%
66%
67%
68%
68%
73%
82%
dosenbach
aal
destrieux
basc_multiscale_122
basc_multiscale_197
harvard_cort_25
smith20
smith70
harvard_sub_25
basc_multiscale_444
msdl
power
basc_multiscale_325
yeo
Stacked_ALFF
Stacked_multi

Feature selection / reduction method :Top 30% Feature Selection

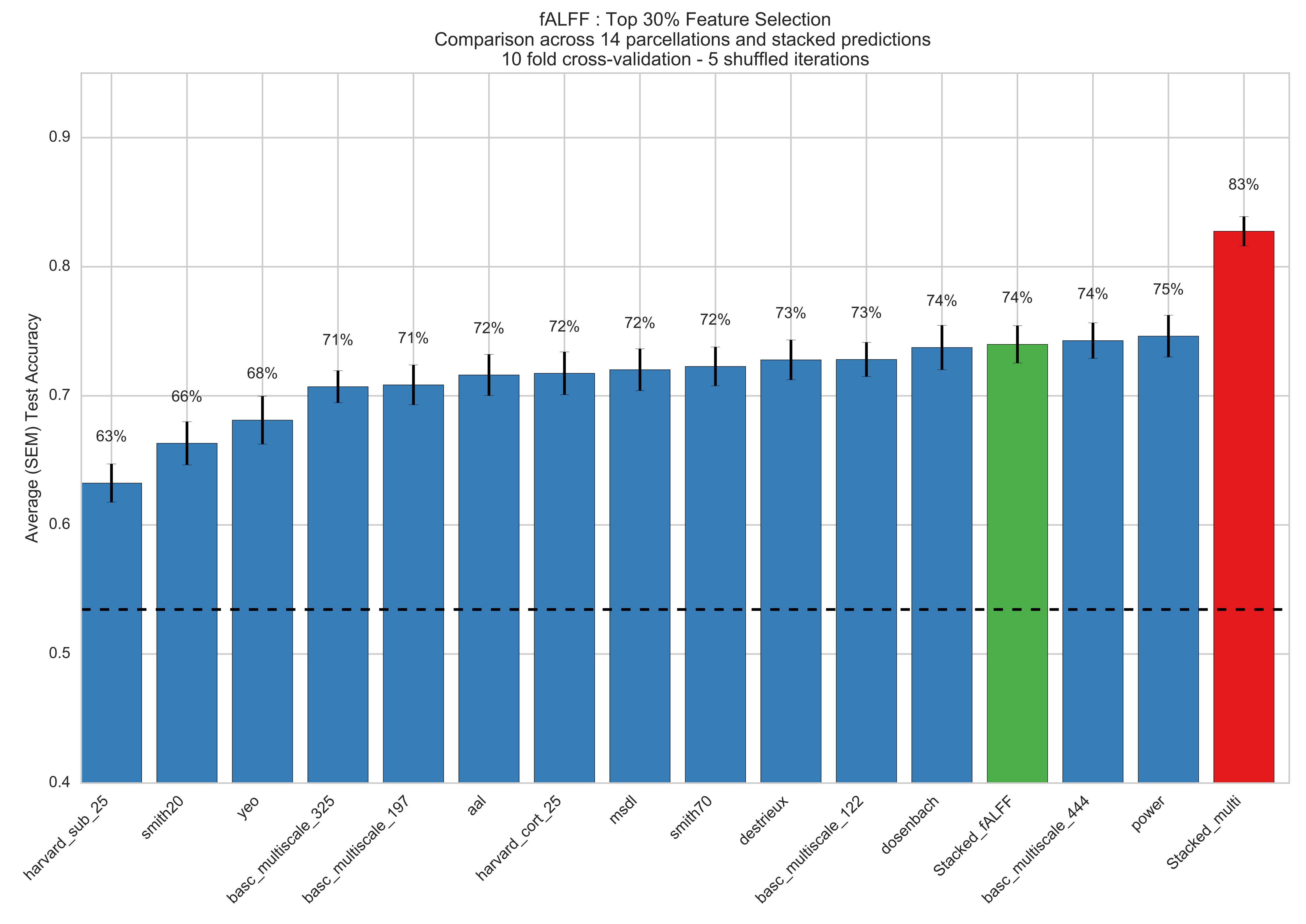

fALFF : Top 30% Feature Selection
Comparison across 14 parcellations and stacked predictions
10 fold cross-validation - 5 shuffled iterations
Average (SEM) Test Accuracy
0.4
0.5
0.6
0.7
0.8
0.9
63%
66%
68%
71%
71%
72%
72%
72%
72%
73%
73%
74%
74%
74%
75%
83%
harvard_sub_25
smith20
yeo
basc_multiscale_325
basc_multiscale_197
aal
harvard_cort_25
msdl
smith70
destrieux
basc_multiscale_122
dosenbach
Stacked_fALFF
basc_multiscale_444
power
Stacked_multi

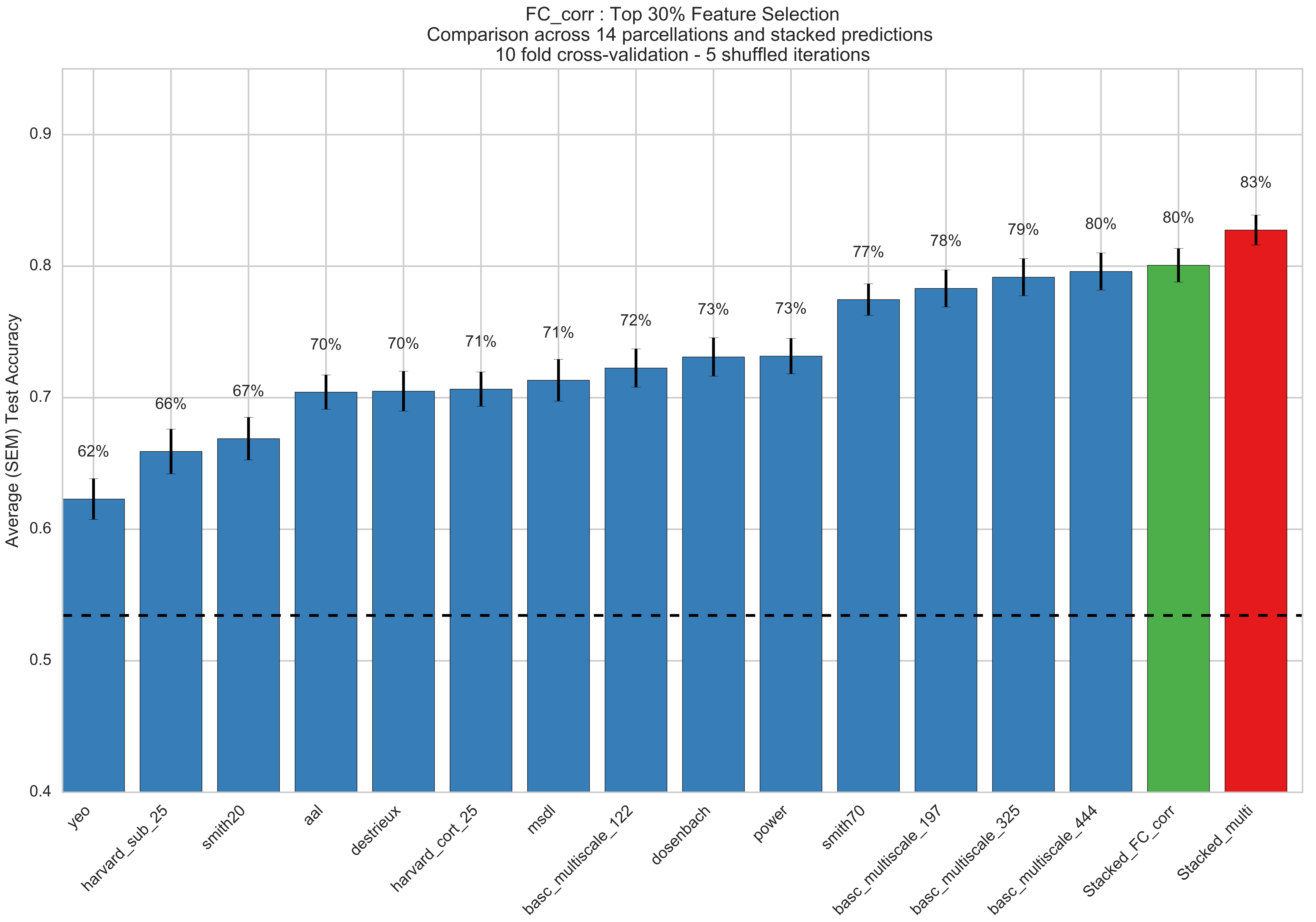
FC_corr : Top 30% Feature Selection
Comparison across 14 parcellations and stacked predictions
10 fold cross-validation - 5 shuffled iterations
Average (SEM) Test Accuracy
0.4
0.5
0.6
0.7
0.8
0.9
62%
66%
67%
70%
70%
71%
71%
72%
73%
73%
77%
78%
79%
80%
80%
83%
yeo
harvard_sub_25
smith20
aal
destrieux
harvard_cort_25
msdl
basc_multiscale_122
dosenbach
power
smith70
basc_multiscale_197
basc_multiscale_325
basc_multiscale_444
Stacked_FC_corr
Stacked_multi

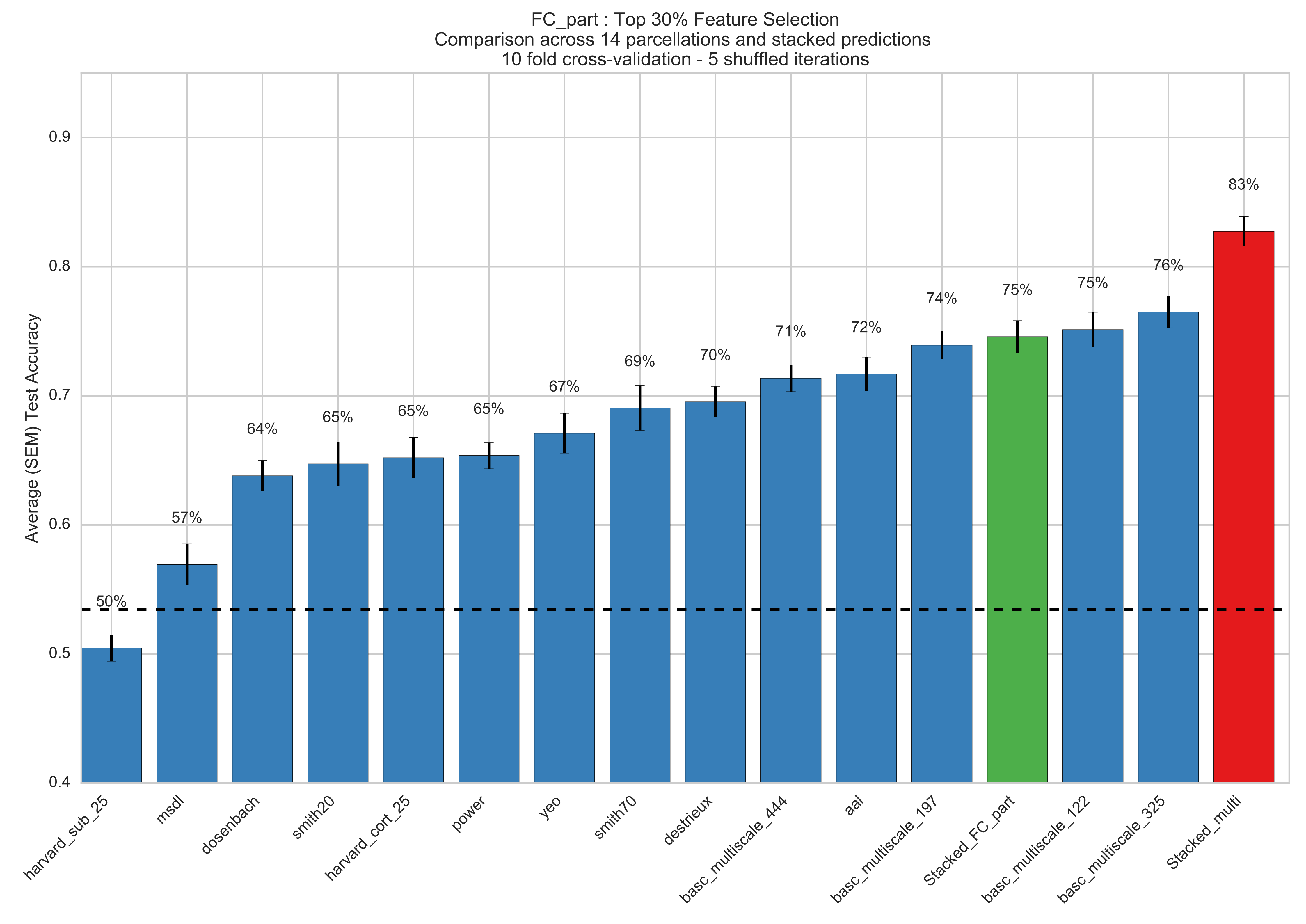
FC_part : Top 30% Feature Selection
Comparison across 14 parcellations and stacked predictions
10 fold cross-validation - 5 shuffled iterations
Average (SEM) Test Accuracy
0.4
0.5
0.6
0.7
0.8
0.9
50%
57%
64%
65%
65%
65%
67%
69%
70%
71%
72%
74%
75%
75%
76%
83%
harvard_sub_25
msdl
dosenbach
smith20
harvard_cort_25
power
yeo
smith70
destrieux
basc_multiscale_444
aal
basc_multiscale_197
Stacked_FC_part
basc_multiscale_122
basc_multiscale_325
Stacked_multi

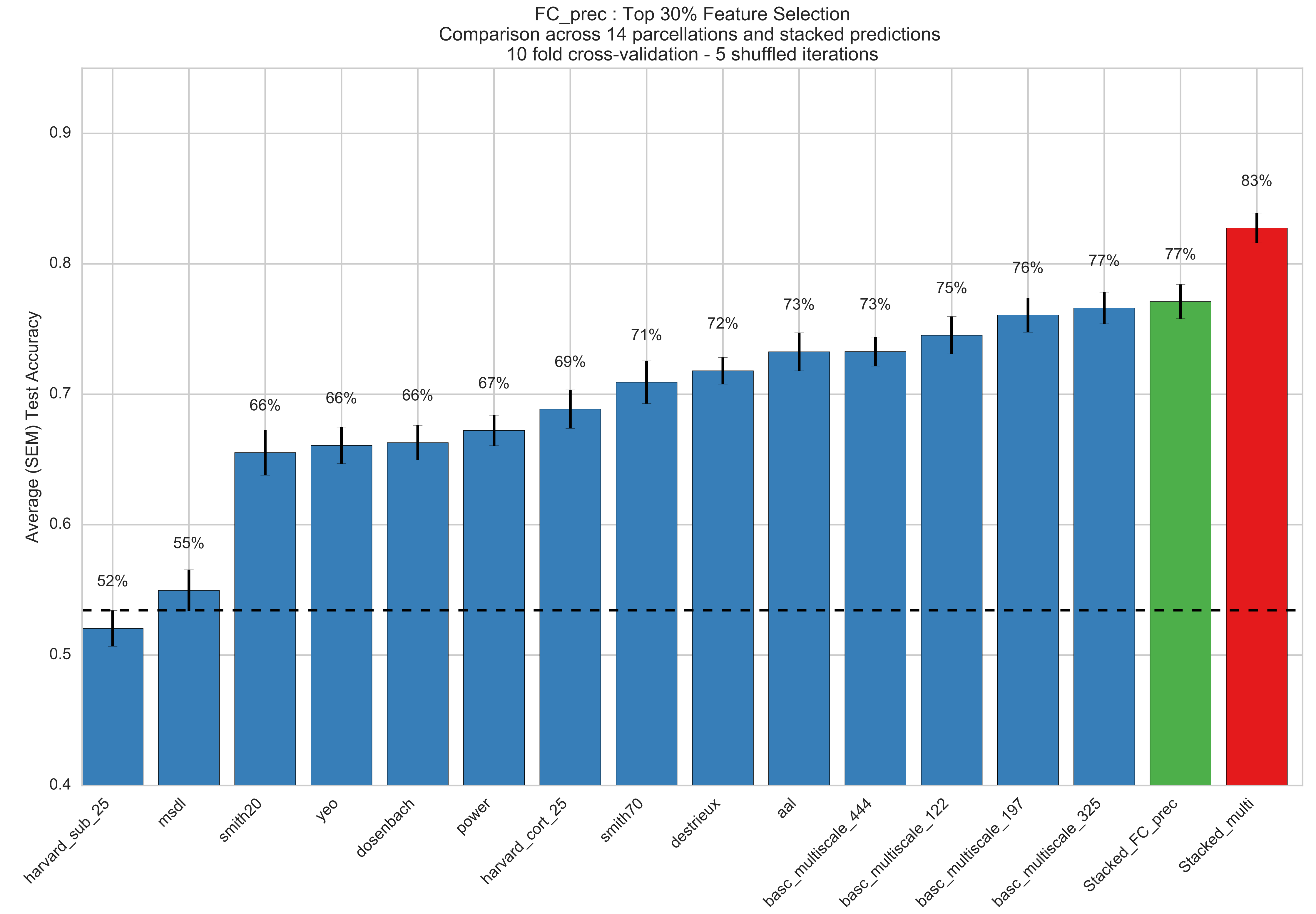
FC_prec : Top 30% Feature Selection
Comparison across 14 parcellations and stacked predictions
10 fold cross-validation - 5 shuffled iterations
Average (SEM) Test Accuracy
0.4
0.5
0.6
0.7
0.8
0.9
52%
55%
66%
66%
66%
67%
69%
71%
72%
73%
73%
75%
76%
77%
77%
83%
harvard_sub_25
msdl
smith20
yeo
dosenbach
power
harvard_cort_25
smith70
destrieux
aal
basc_multiscale_444
basc_multiscale_122
basc_multiscale_197
basc_multiscale_325
Stacked_FC_prec
Stacked_multi

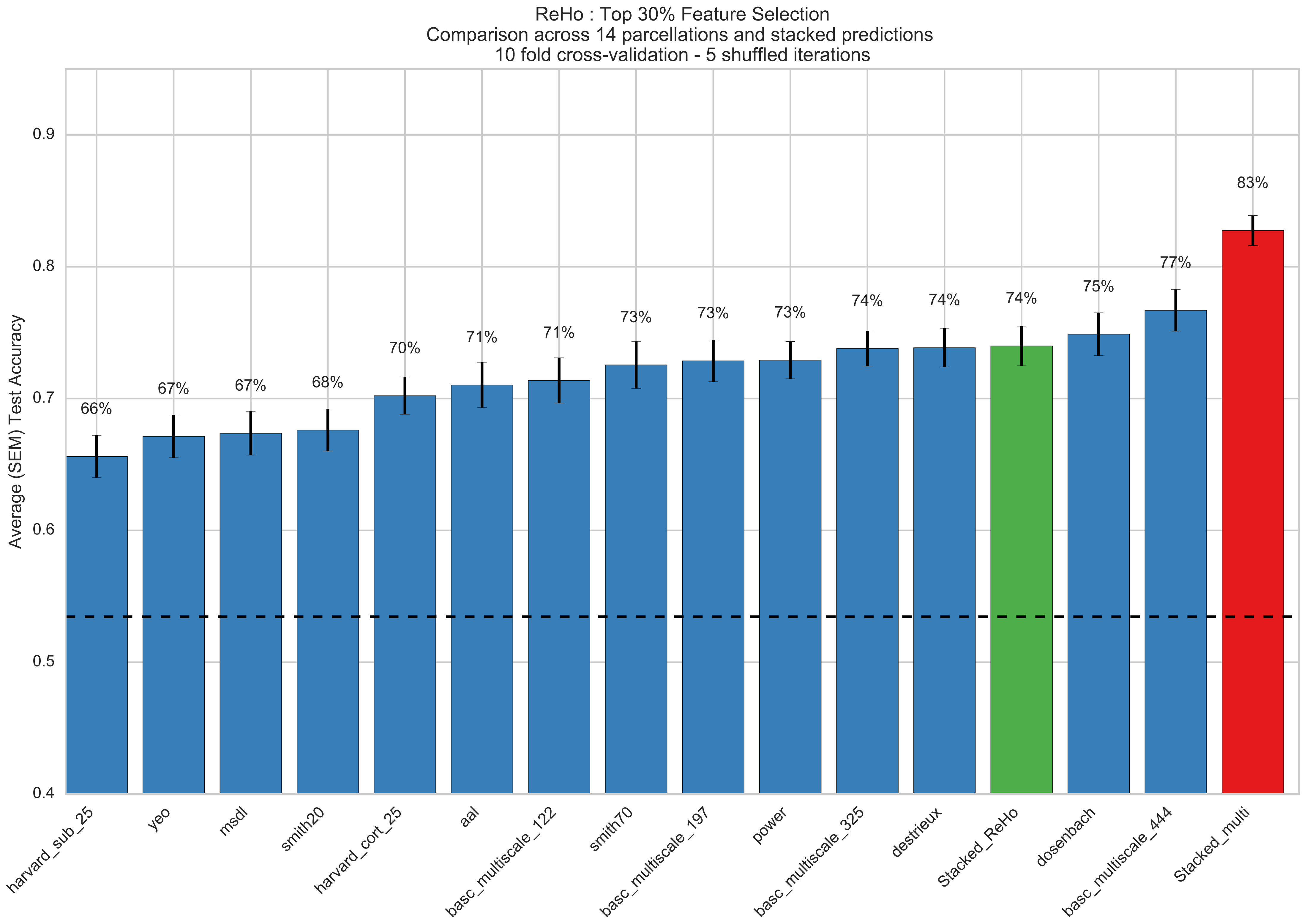

ReHo : Top 30% Feature Selection
Comparison across 14 parcellations and stacked predictions
10 fold cross-validation - 5 shuffled iterations
Average (SEM) Test Accuracy
0.4
0.5
0.6
0.7
0.8
0.9
66%
67%
67%
68%
70%
71%
71%
73%
73%
73%
74%
74%
74%
75%
77%
83%
harvard_sub_25
yeo
msdl
smith20
harvard_cort_25
aal
basc_multiscale_122
smith70
basc_multiscale_197
power
basc_multiscale_325
destrieux
Stacked_ReHo
dosenbach
basc_multiscale_444
Stacked_multi

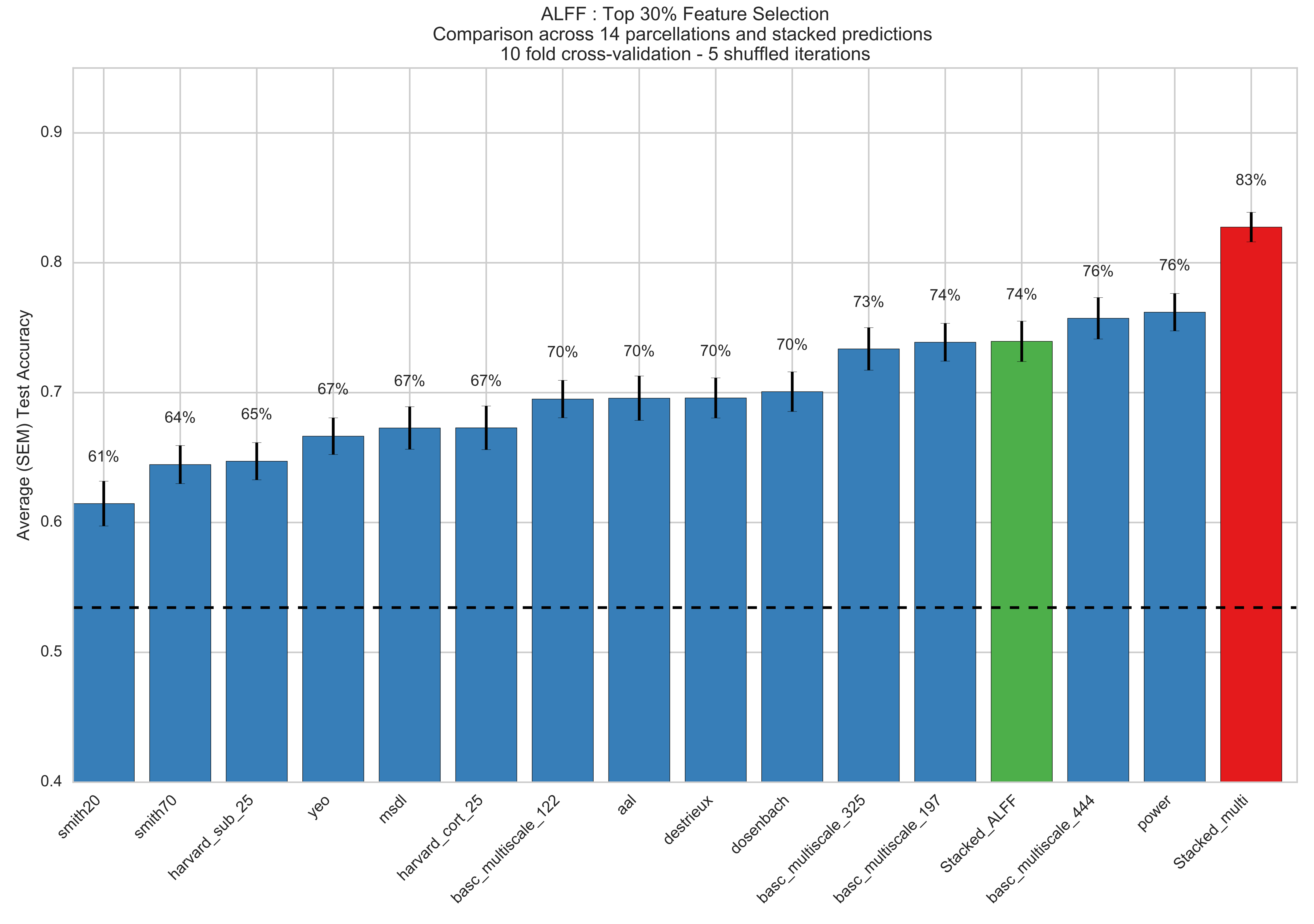
ALFF : Top 30% Feature Selection
Comparison across 14 parcellations and stacked predictions
10 fold cross-validation - 5 shuffled iterations
Average (SEM) Test Accuracy
0.4
0.5
0.6
0.7
0.8
0.9
61%
64%
65%
67%
67%
67%
70%
70%
70%
70%
73%
74%
74%
76%
76%
83%
smith20
smith70
harvard_sub_25
yeo
msdl
harvard_cort_25
basc_multiscale_122
aal
destrieux
dosenbach
basc_multiscale_325
basc_multiscale_197
Stacked_ALFF
basc_multiscale_444
power
Stacked_multi

Feature selection / reduction method :Top 1% Feature Selection

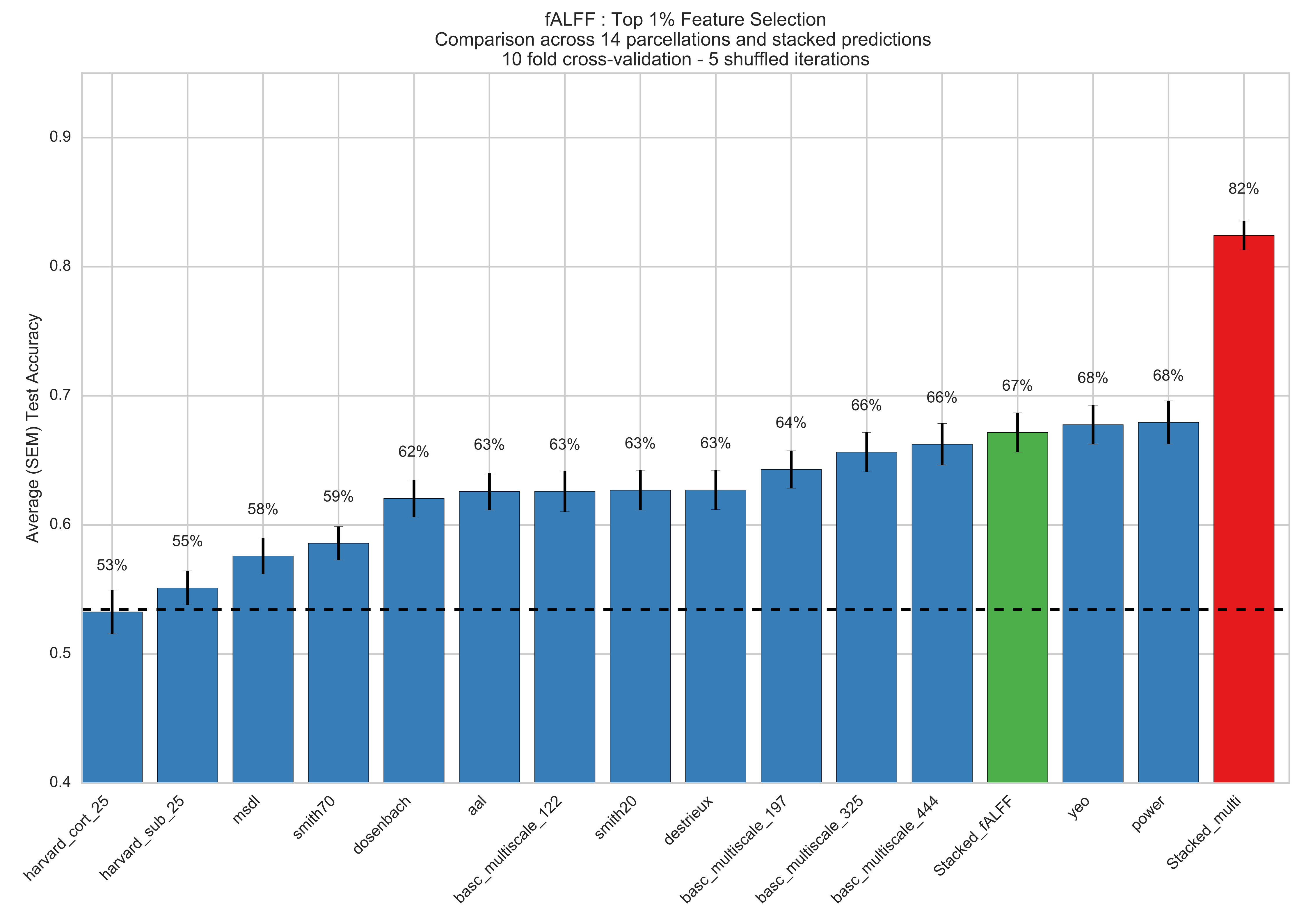
fALFF : Top 1% Feature Selection
Comparison across 14 parcellations and stacked predictions
10 fold cross-validation - 5 shuffled iterations
Average (SEM) Test Accuracy
0.4
0.5
0.6
0.7
0.8
0.9
53%
55%
58%
59%
62%
63%
63%
63%
63%
64%
66%
66%
67%
68%
68%
82%
harvard_cort_25
harvard_sub_25
msdl
smith70
dosenbach
aal
basc_multiscale_122
smith20
destrieux
basc_multiscale_197
basc_multiscale_325
basc_multiscale_444
Stacked_fALFF
yeo
power
Stacked_multi

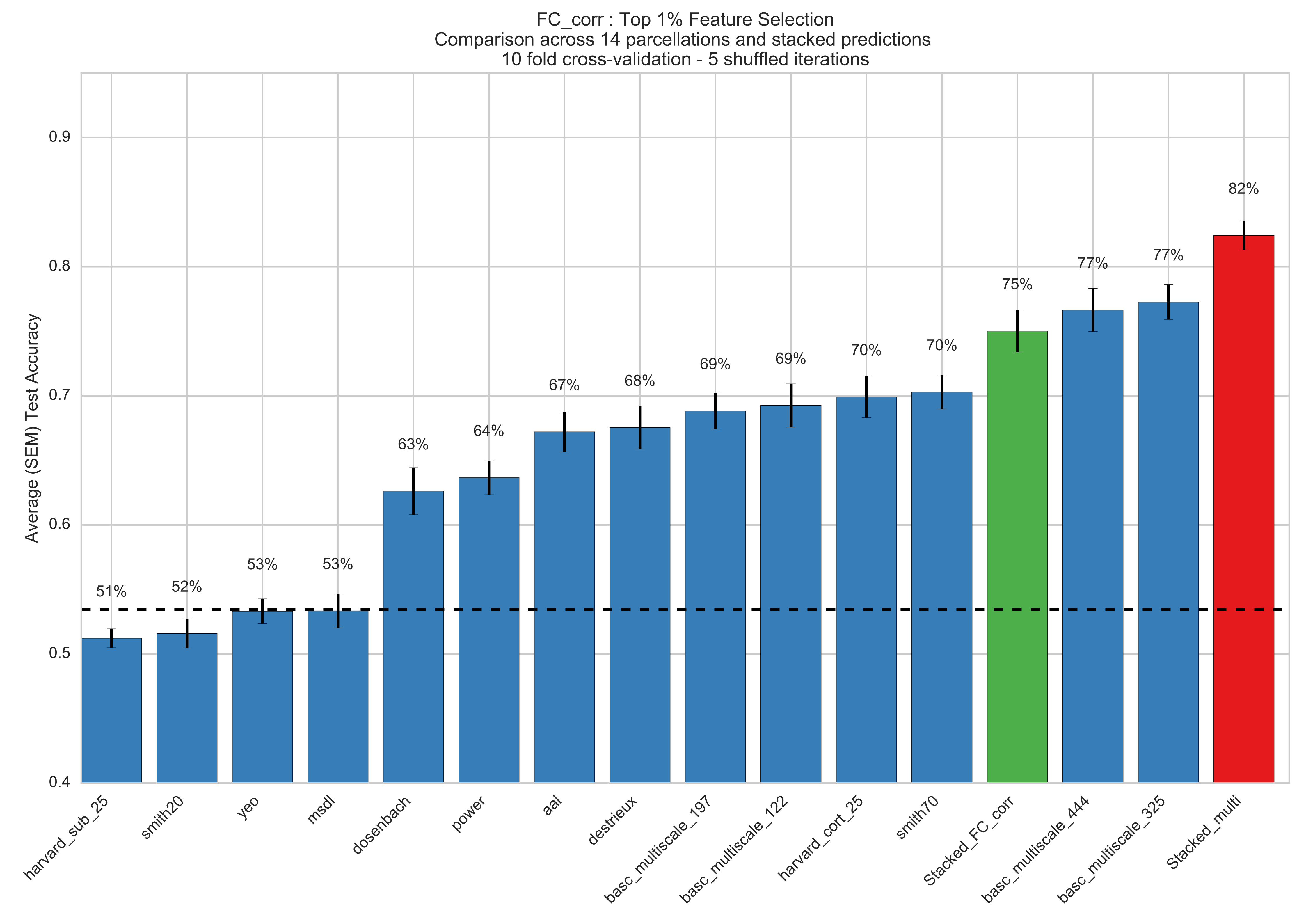
FC_corr : Top 1% Feature Selection
Comparison across 14 parcellations and stacked predictions
10 fold cross-validation - 5 shuffled iterations
Average (SEM) Test Accuracy
0.9
0.8
0.7
0.6
0.5
0.4
51%
52%
53%
53%
63%
64%
67%
68%
69%
69%
70%
70%
75%
77%
77%
82%
harvard_sub_25
smith20
yeo
msdl
dosenbach
power
aal
destrieux
basc_multiscale_197
basc_multiscale_122
harvard_cort_25
smith70
Stacked_FC_corr
basc_multiscale_444
basc_multiscale_325
Stacked_multi

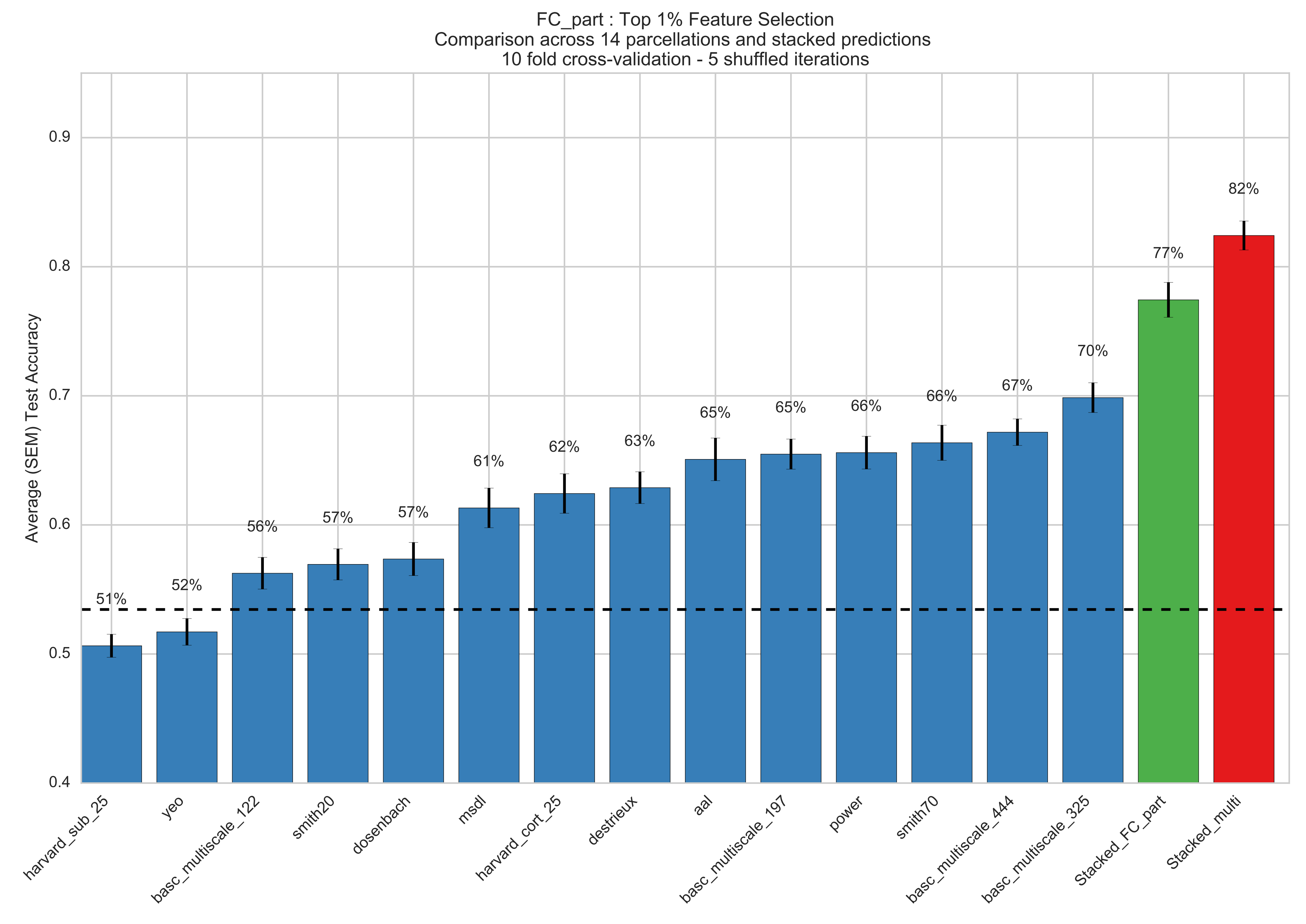
FC_part : Top 1% Feature Selection
Comparison across 14 parcellations and stacked predictions
10 fold cross-validation - 5 shuffled iterations
Average (SEM) Test Accuracy
0.4
0.5
0.6
0.7
0.8
0.9
51%
52%
56%
57%
57%
61%
62%
63%
65%
65%
66%
66%
67%
70%
77%
82%
harvard_sub_25
yeo
basc_multiscale_122
smith20
dosenbach
msdl
harvard_cort_25
destrieux
aal
basc_multiscale_197
power
smith70
basc_multiscale_444
basc_multiscale_325
Stacked_FC_part
Stacked_multi

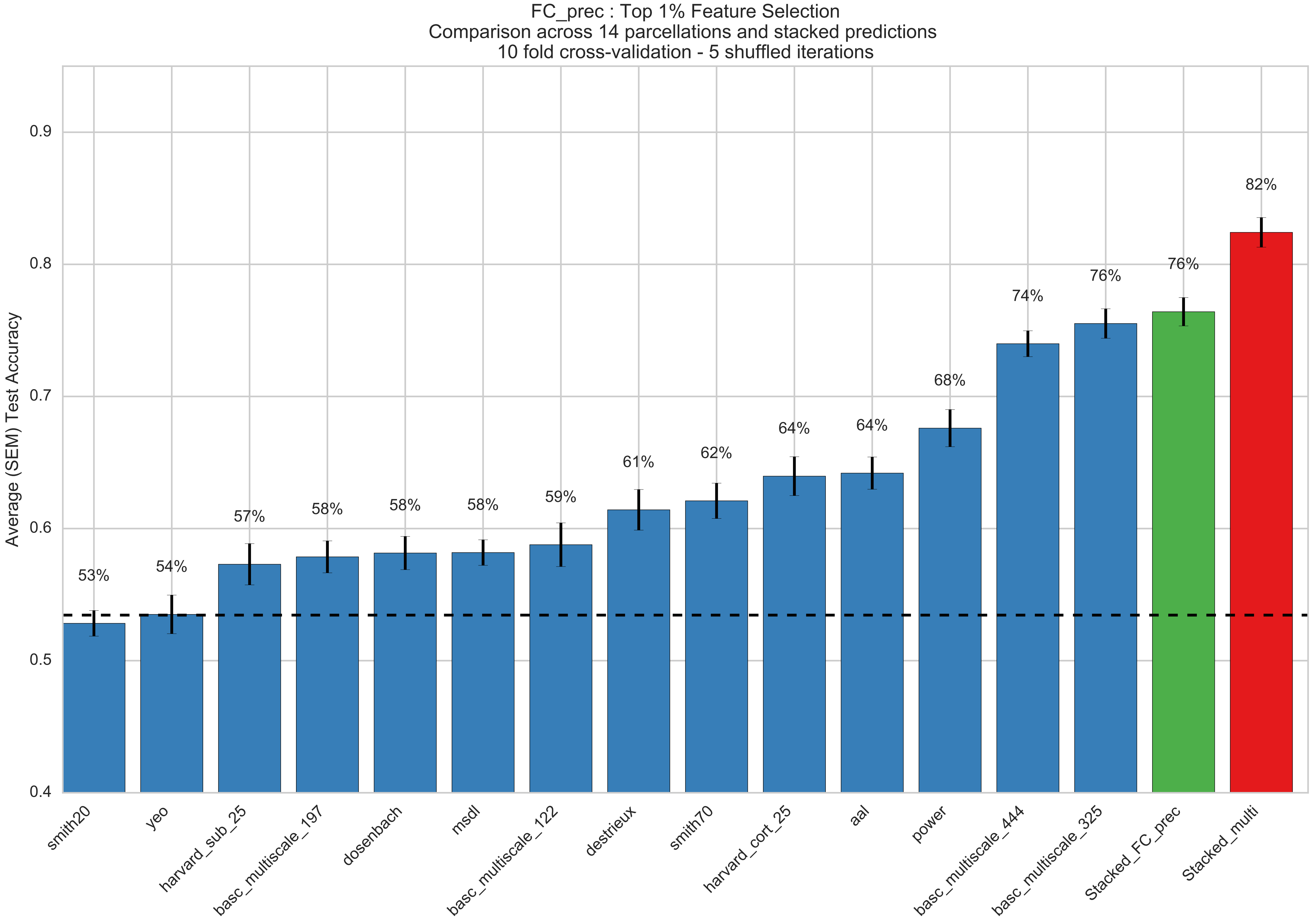
FC_prec : Top 1% Feature Selection
Comparison across 14 parcellations and stacked predictions
10 fold cross-validation - 5 shuffled iterations
Average (SEM) Test Accuracy
0.4
0.5
0.6
0.7
0.8
0.9
53%
54%
57%
58%
58%
58%
59%
61%
62%
64%
64%
68%
74%
76%
76%
82%
smith20
yeo
harvard_sub_25
basc_multiscale_197
dosenbach
msdl
basc_multiscale_122
destrieux
smith70
harvard_cort_25
aal
power
basc_multiscale_444
basc_multiscale_325
Stacked_FC_prec
Stacked_multi

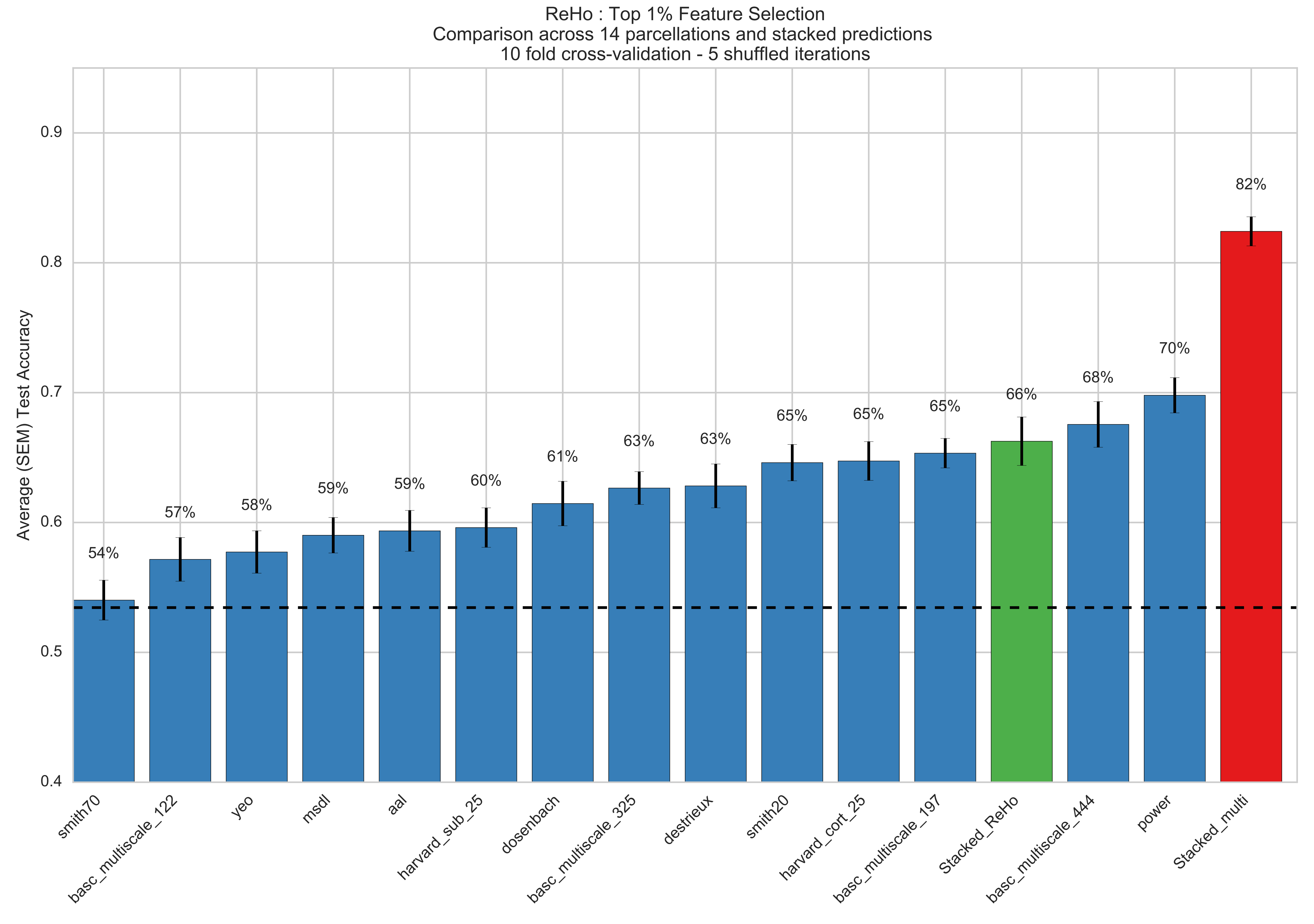
ReHo : Top 1% Feature Selection
Comparison across 14 parcellations and stacked predictions
10 fold cross-validation - 5 shuffled iterations
Average (SEM) Test Accuracy
0.9
0.8
0.7
0.6
0.5
0.4
54%
57%
58%
59%
59%
60%
61%
63%
63%
65%
65%
65%
66%
68%
70%
82%
smith70
basc_multiscale_122
yeo
msdl
aal
harvard_sub_25
dosenbach
basc_multiscale_325
destrieux
smith20
harvard_cort_25
basc_multiscale_197
Stacked_ReHo
basc_multiscale_444
power
Stacked_multi

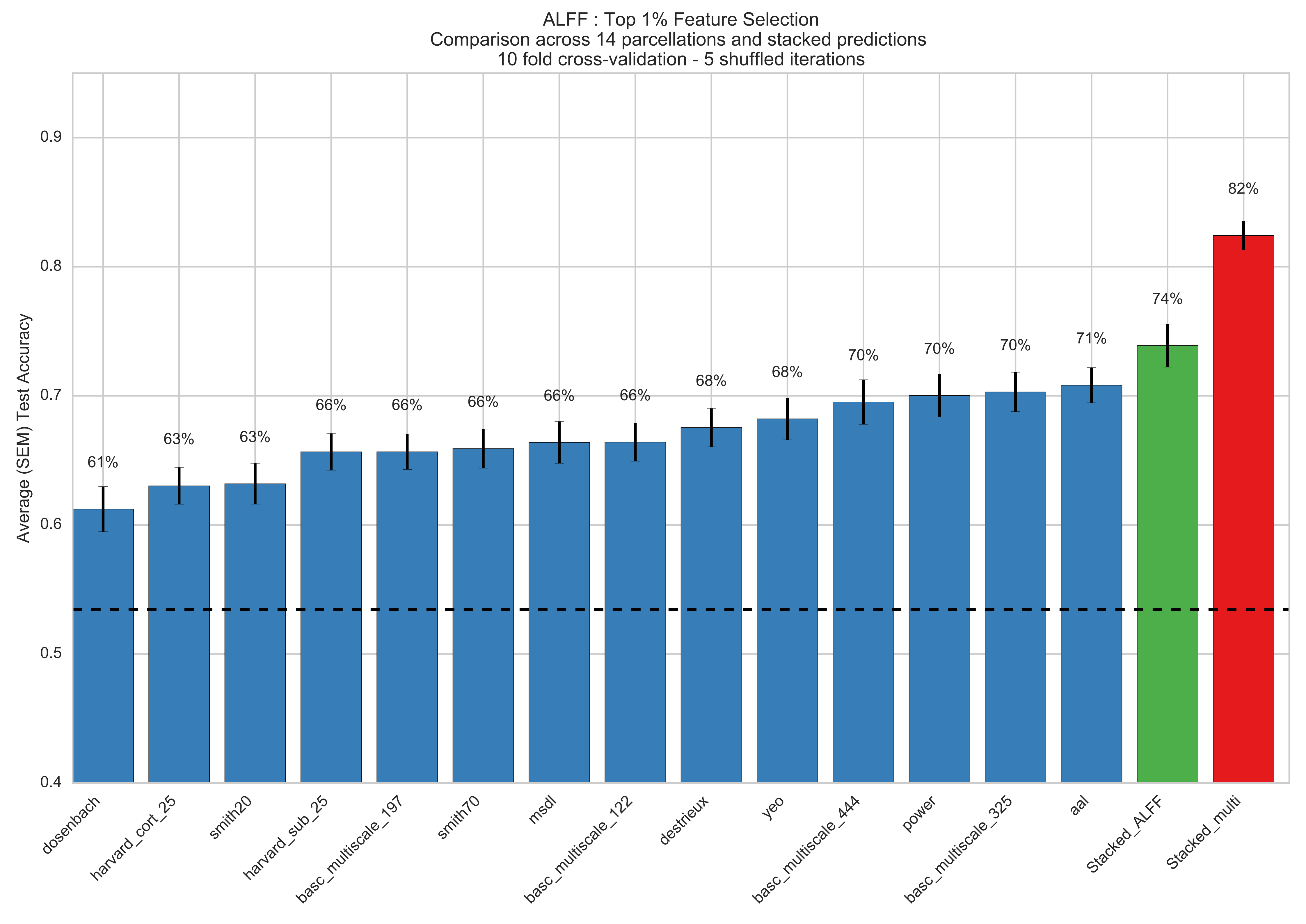
ALFF : Top 1% Feature Selection
Comparison across 14 parcellations and stacked predictions
10 fold cross-validation - 5 shuffled iterations
Average (SEM) Test Accuracy
0.4
0.5
0.6
0.7
0.8
0.9
61%
63%
63%
66%
66%
66%
66%
66%
68%
68%
70%
70%
70%
71%
74%
82%
dosenbach
harvard_cort_25
smith20
harvard_sub_25
basc_multiscale_197
smith70
msdl
basc_multiscale_122
destrieux
yeo
basc_multiscale_444
power
basc_multiscale_325
aal
Stacked_ALFF
Stacked_multi

Feature selection / reduction method :Top 30% FC, 100% regional Feature Selection

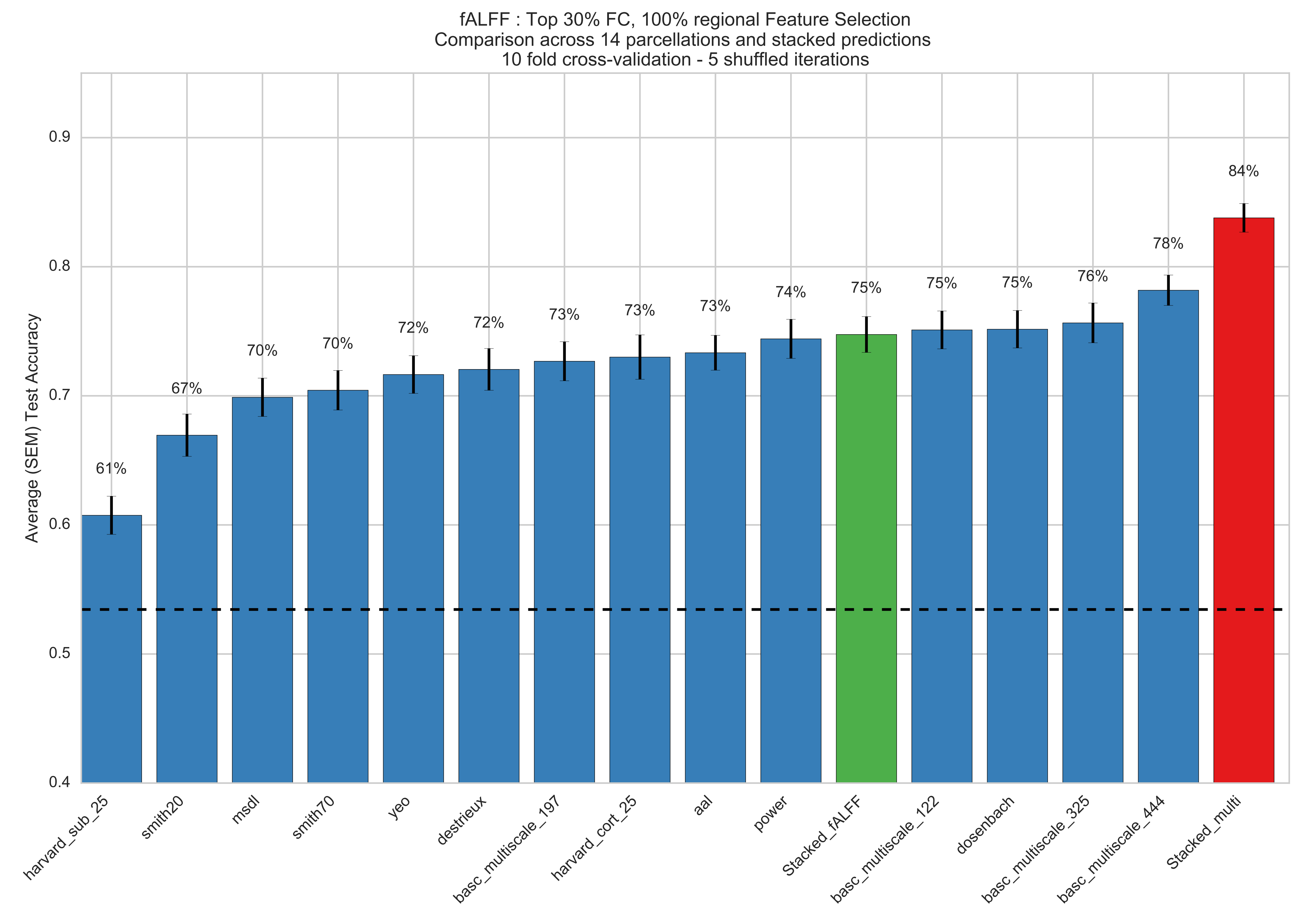

fALFF : Top 30% FC, 100% regional Feature Selection
Comparison across 14 parcellations and stacked predictions
10 fold cross-validation - 5 shuffled iterations
Average (SEM) Test Accuracy
0.4
0.5
0.6
0.7
0.8
0.9
61%
67%
70%
70%
72%
72%
73%
73%
73%
74%
75%
75%
75%
76%
78%
84%
harvard_sub_25
smith20
msdl
smith70
yeo
destrieux
basc_multiscale_197
harvard_cort_25
aal
power
Stacked_fALFF
basc_multiscale_122
dosenbach
basc_multiscale_325
basc_multiscale_444
Stacked_multi

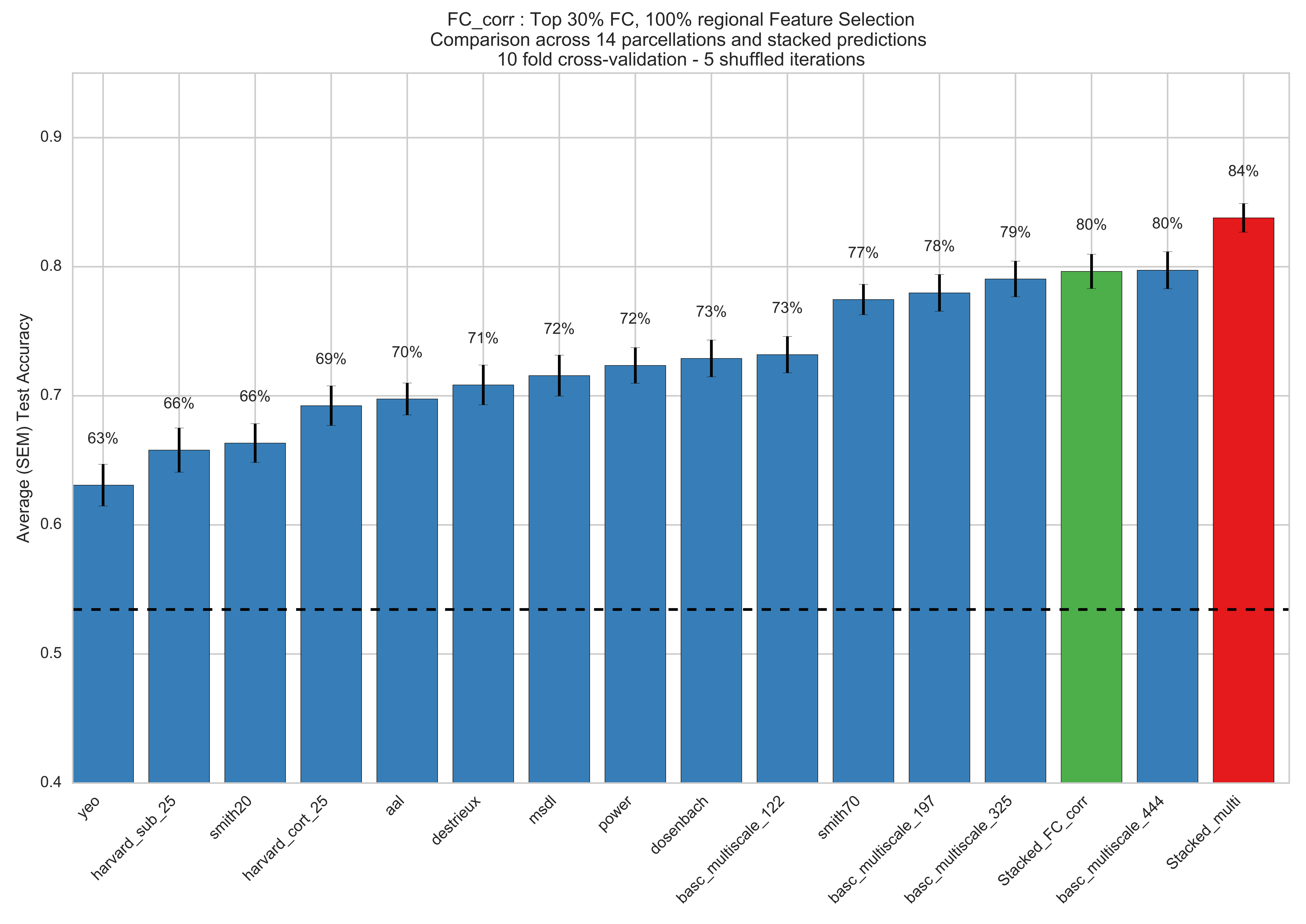

FC_corr : Top 30% FC, 100% regional Feature Selection
Comparison across 14 parcellations and stacked predictions
10 fold cross-validation - 5 shuffled iterations
Average (SEM) Test Accuracy
0.4
0.5
0.6
0.7
0.8
0.9
63%
66%
66%
69%
70%
71%
72%
72%
73%
73%
77%
78%
79%
80%
80%
84%
yeo
harvard_sub_25
smith20
harvard_cort_25
aal
destrieux
msdl
power
dosenbach
basc_multiscale_122
smith70
basc_multiscale_197
basc_multiscale_325
Stacked_FC_corr
basc_multiscale_444
Stacked_multi

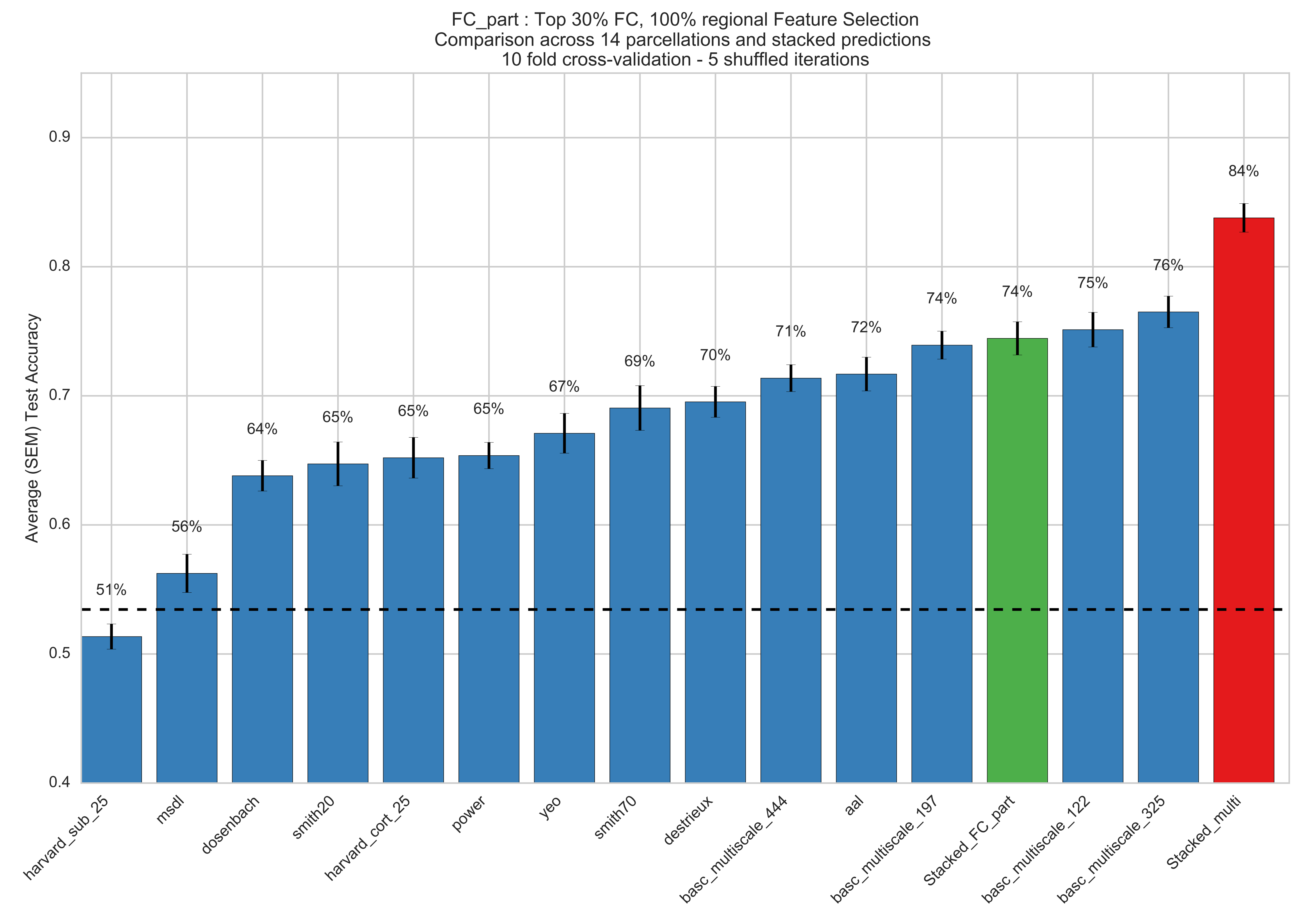
FC_part : Top 30% FC, 100% regional Feature Selection
Comparison across 14 parcellations and stacked predictions
10 fold cross-validation - 5 shuffled iterations
Average (SEM) Test Accuracy
0.4
0.5
0.6
0.7
0.8
0.9
51%
56%
64%
65%
65%
65%
67%
69%
70%
71%
72%
74%
74%
75%
76%
84%
harvard_sub_25
msdl
dosenbach
smith20
harvard_cort_25
power
yeo
smith70
destrieux
basc_multiscale_444
aal
basc_multiscale_197
Stacked_FC_part
basc_multiscale_122
basc_multiscale_325
Stacked_multi

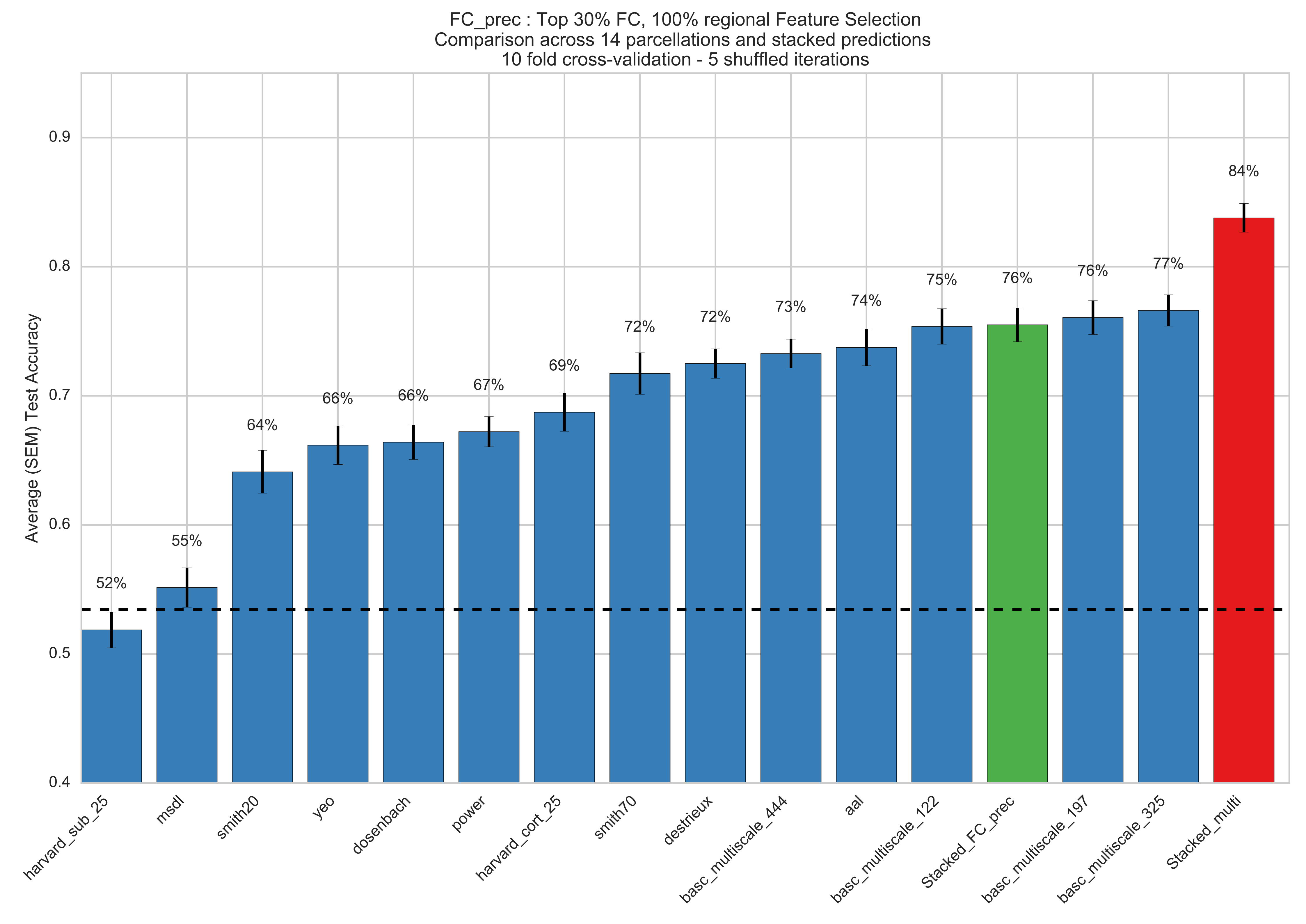

FC_prec : Top 30% FC, 100% regional Feature Selection
Comparison across 14 parcellations and stacked predictions
10 fold cross-validation - 5 shuffled iterations
Average (SEM) Test Accuracy
0.4
0.5
0.6
0.7
0.8
0.9
52%
55%
64%
66%
66%
67%
69%
72%
72%
73%
74%
75%
76%
76%
77%
84%
harvard_sub_25
msdl
smith20
yeo
dosenbach
power
harvard_cort_25
smith70
destrieux
basc_multiscale_444
aal
basc_multiscale_122
Stacked_FC_prec
basc_multiscale_197
basc_multiscale_325
Stacked_multi

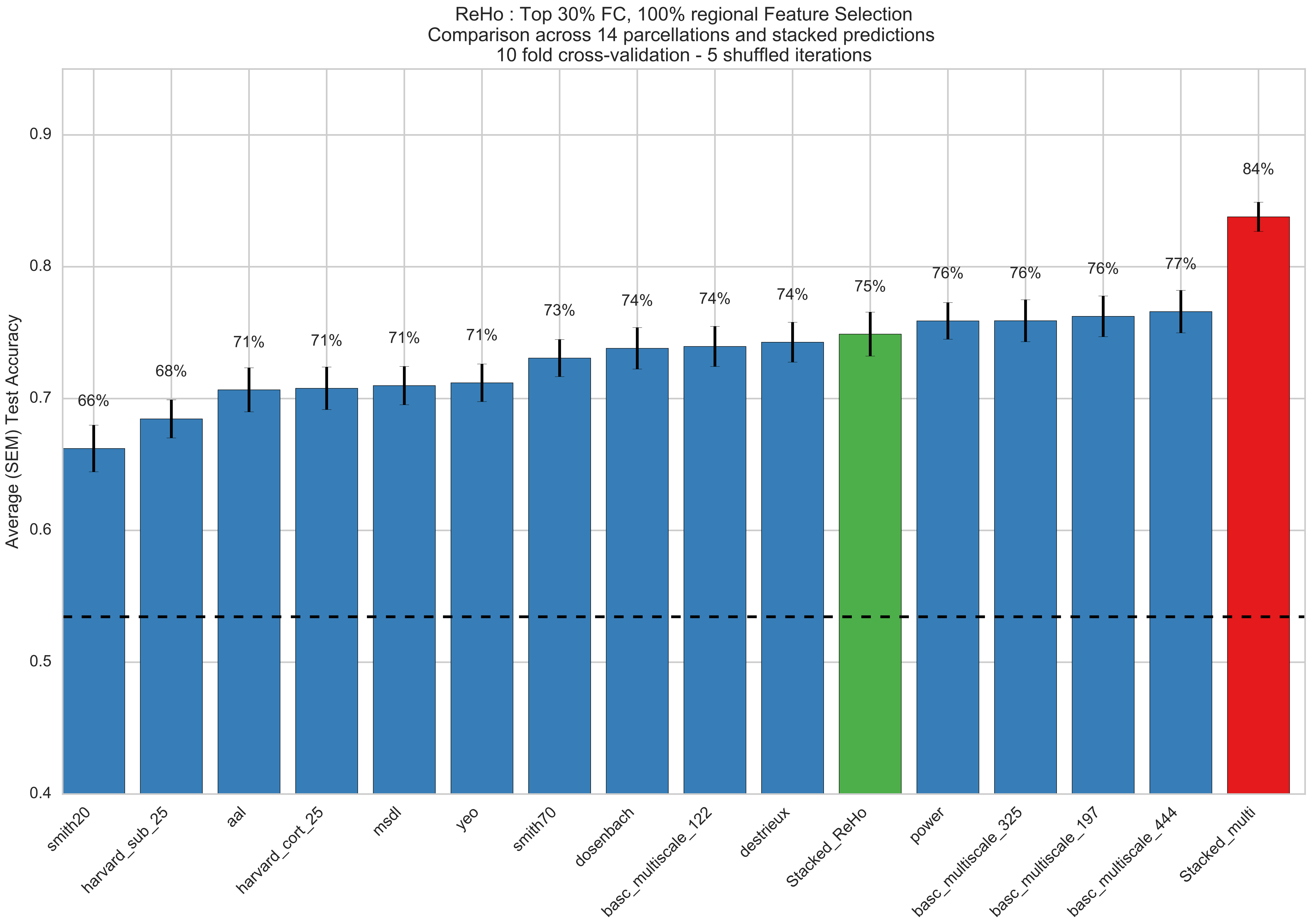

ReHo : Top 30% FC, 100% regional Feature Selection
Comparison across 14 parcellations and stacked predictions
10 fold cross-validation - 5 shuffled iterations
Average (SEM) Test Accuracy
0.4
0.5
0.6
0.7
0.8
0.9
66%
68%
71%
71%
71%
71%
73%
74%
74%
74%
75%
76%
76%
76%
77%
84%
smith20
harvard_sub_25
aal
harvard_cort_25
msdl
yeo
smith70
dosenbach
basc_multiscale_122
destrieux
Stacked_ReHo
power
basc_multiscale_325
basc_multiscale_197
basc_multiscale_444
Stacked_multi

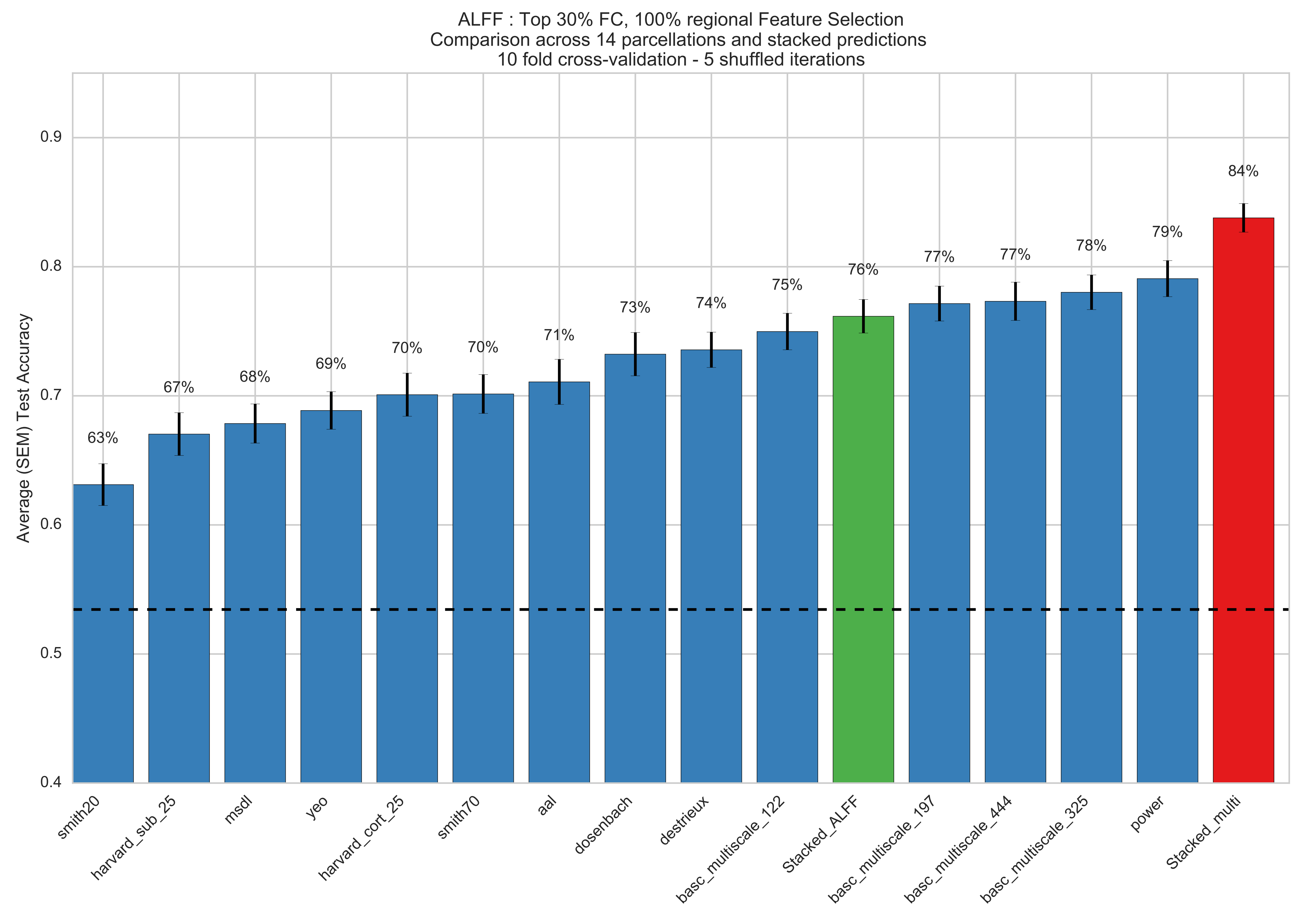
ALFF : Top 30% FC, 100% regional Feature Selection
Comparison across 14 parcellations and stacked predictions
10 fold cross-validation - 5 shuffled iterations
Average (SEM) Test Accuracy
0.4
0.5
0.6
0.7
0.8
0.9
63%
67%
68%
69%
70%
70%
71%
73%
74%
75%
76%
77%
77%
78%
79%
84%
smith20
harvard_sub_25
msdl
yeo
harvard_cort_25
smith70
aal
dosenbach
destrieux
basc_multiscale_122
Stacked_ALFF
basc_multiscale_197
basc_multiscale_444
basc_multiscale_325
power
Stacked_multi

Feature selection / reduction method :Top 20% Feature Selection

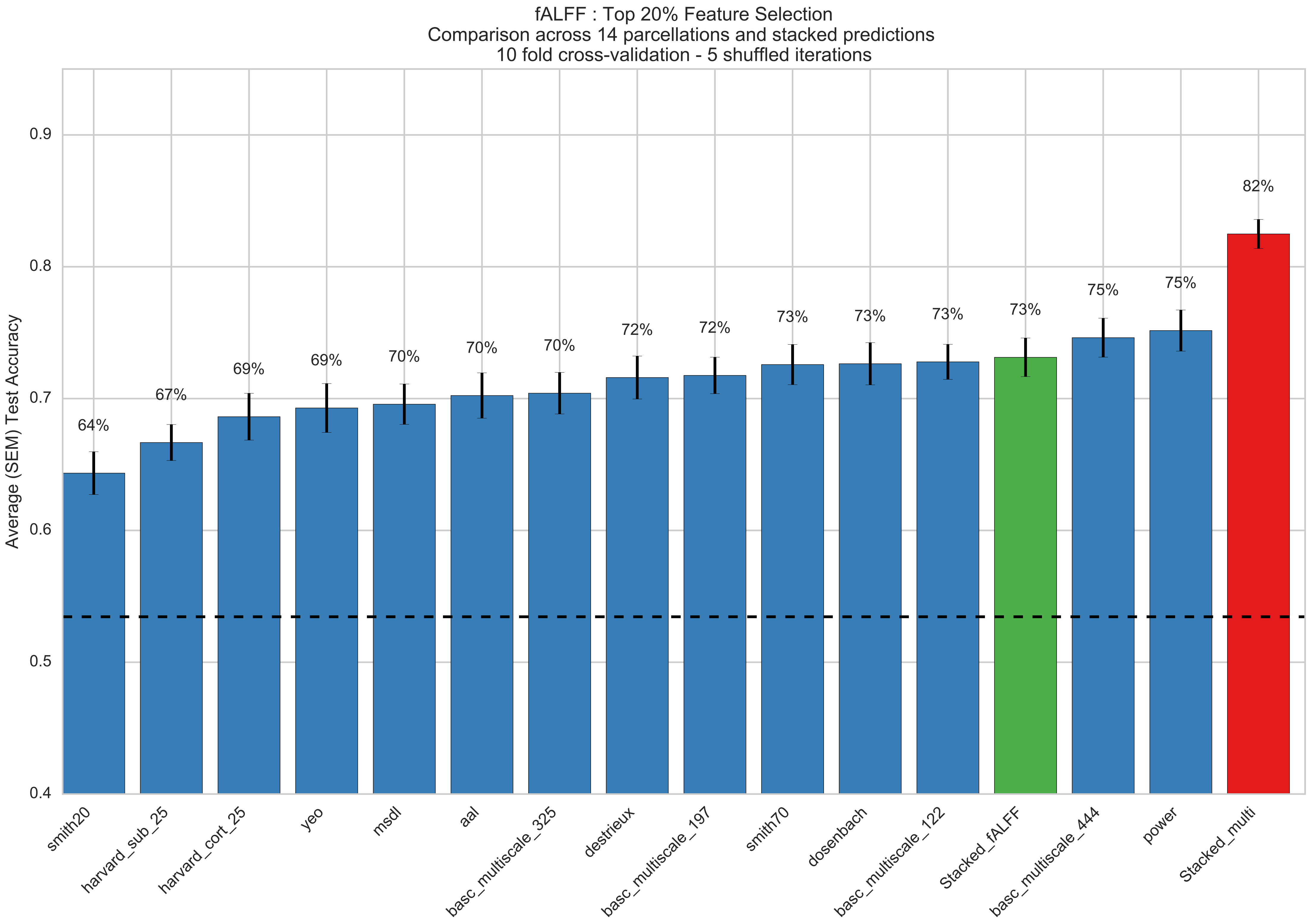
fALFF : Top 20% Feature Selection
Comparison across 14 parcellations and stacked predictions
10 fold cross-validation - 5 shuffled iterations
Average (SEM) Test Accuracy
0.4
0.5
0.6
0.7
0.8
0.9
64%
67%
69%
69%
70%
70%
70%
72%
72%
73%
73%
73%
73%
75%
75%
82%
smith20
harvard_sub_25
harvard_cort_25
yeo
msdl
aal
basc_multiscale_325
destrieux
basc_multiscale_197
smith70
dosenbach
basc_multiscale_122
Stacked_fALFF
basc_multiscale_444
power
Stacked_multi

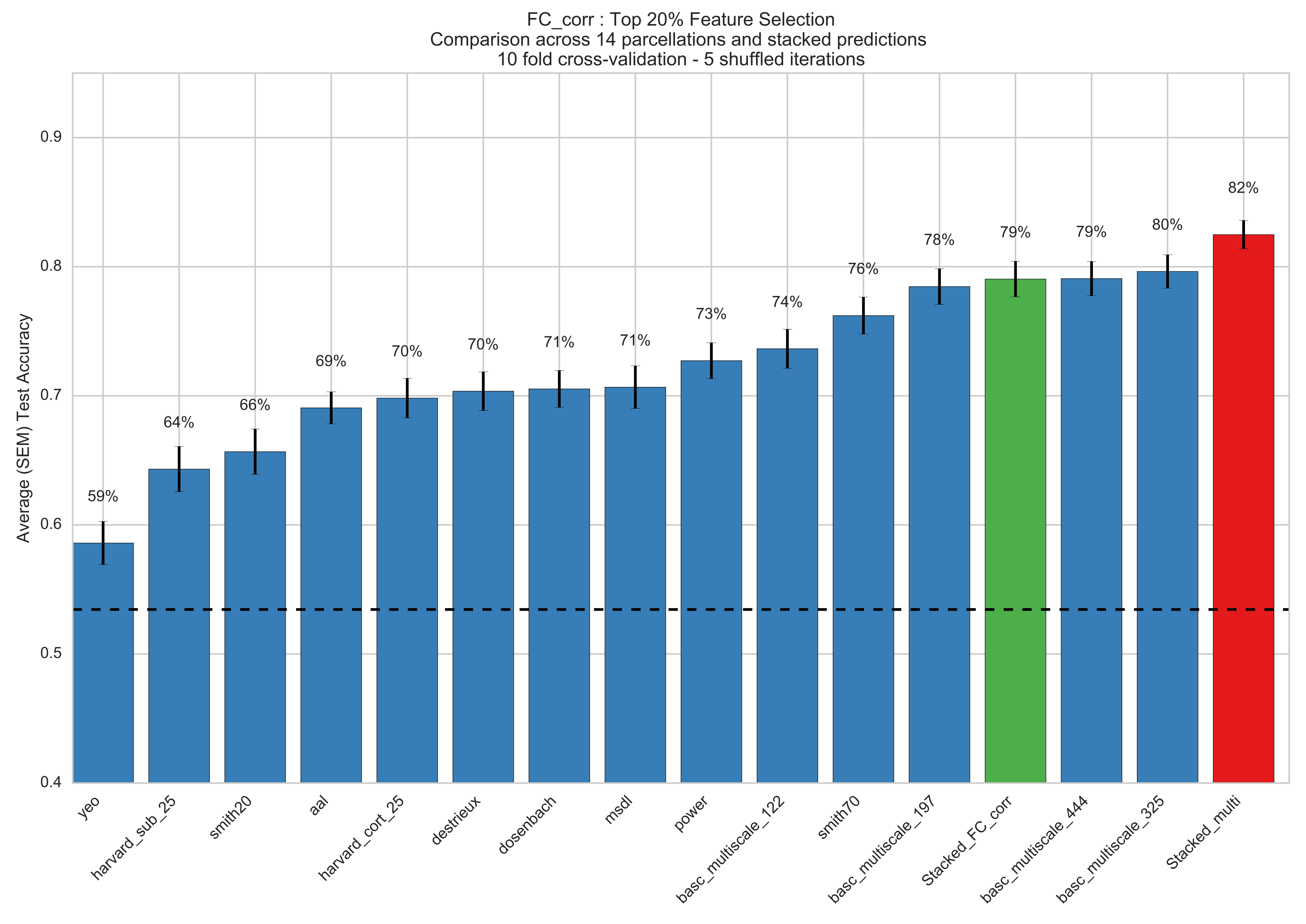

FC_corr : Top 20% Feature Selection
Comparison across 14 parcellations and stacked predictions
10 fold cross-validation - 5 shuffled iterations
Average (SEM) Test Accuracy
0.4
0.5
0.6
0.7
0.8
0.9
59%
64%
66%
69%
70%
70%
71%
71%
73%
74%
76%
78%
79%
79%
80%
82%
yeo
harvard_sub_25
smith20
aal
harvard_cort_25
destrieux
dosenbach
msdl
power
basc_multiscale_122
smith70
basc_multiscale_197
Stacked_FC_corr
basc_multiscale_444
basc_multiscale_325
Stacked_multi

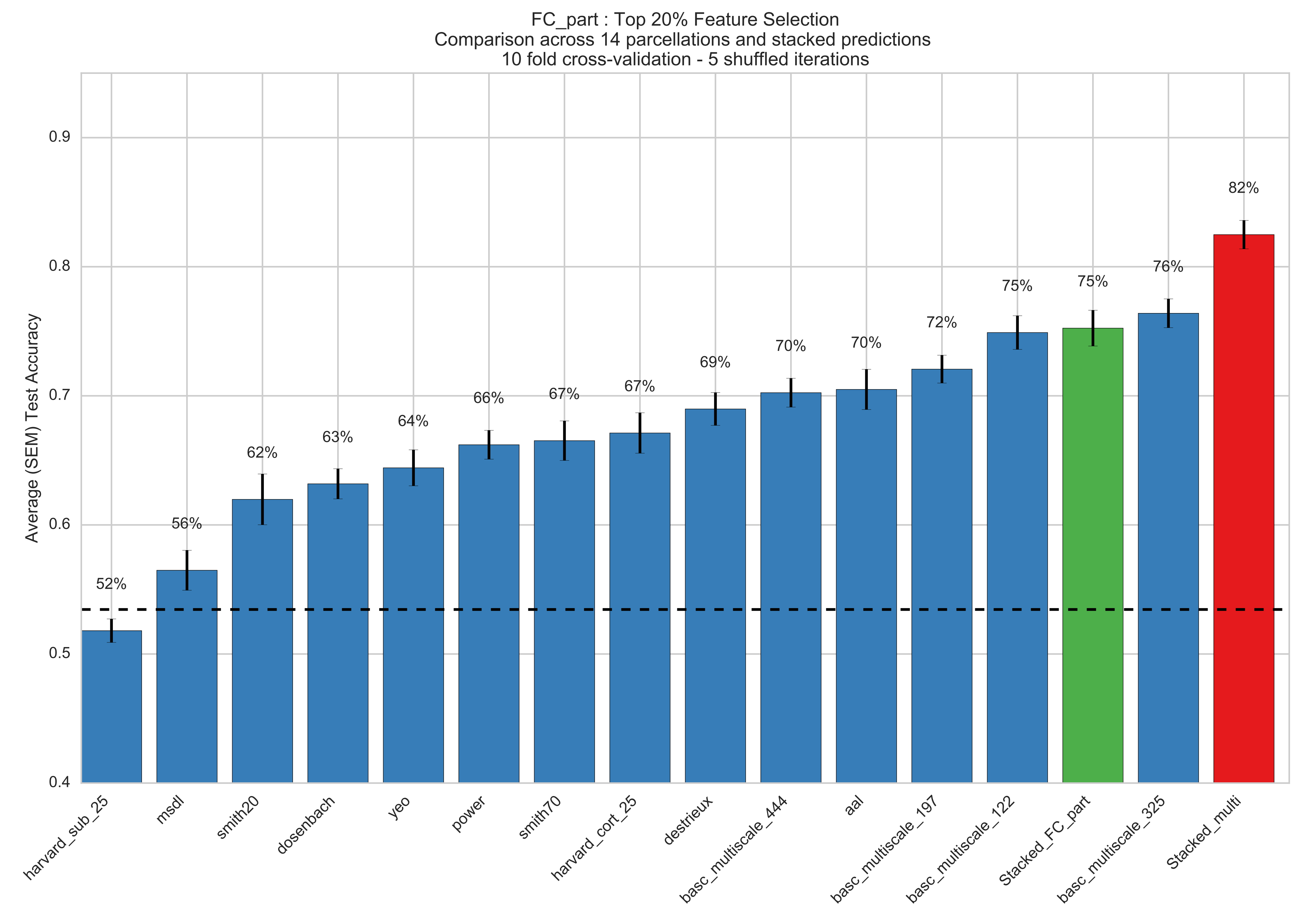
FC_part : Top 20% Feature Selection
Comparison across 14 parcellations and stacked predictions
10 fold cross-validation - 5 shuffled iterations
Average (SEM) Test Accuracy
0.4
0.5
0.6
0.7
0.8
0.9
52%
56%
62%
63%
64%
66%
67%
67%
69%
70%
70%
72%
75%
75%
76%
82%
harvard_sub_25
msdl
smith20
dosenbach
yeo
power
smith70
harvard_cort_25
destrieux
basc_multiscale_444
aal
basc_multiscale_197
basc_multiscale_122
Stacked_FC_part
basc_multiscale_325
Stacked_multi

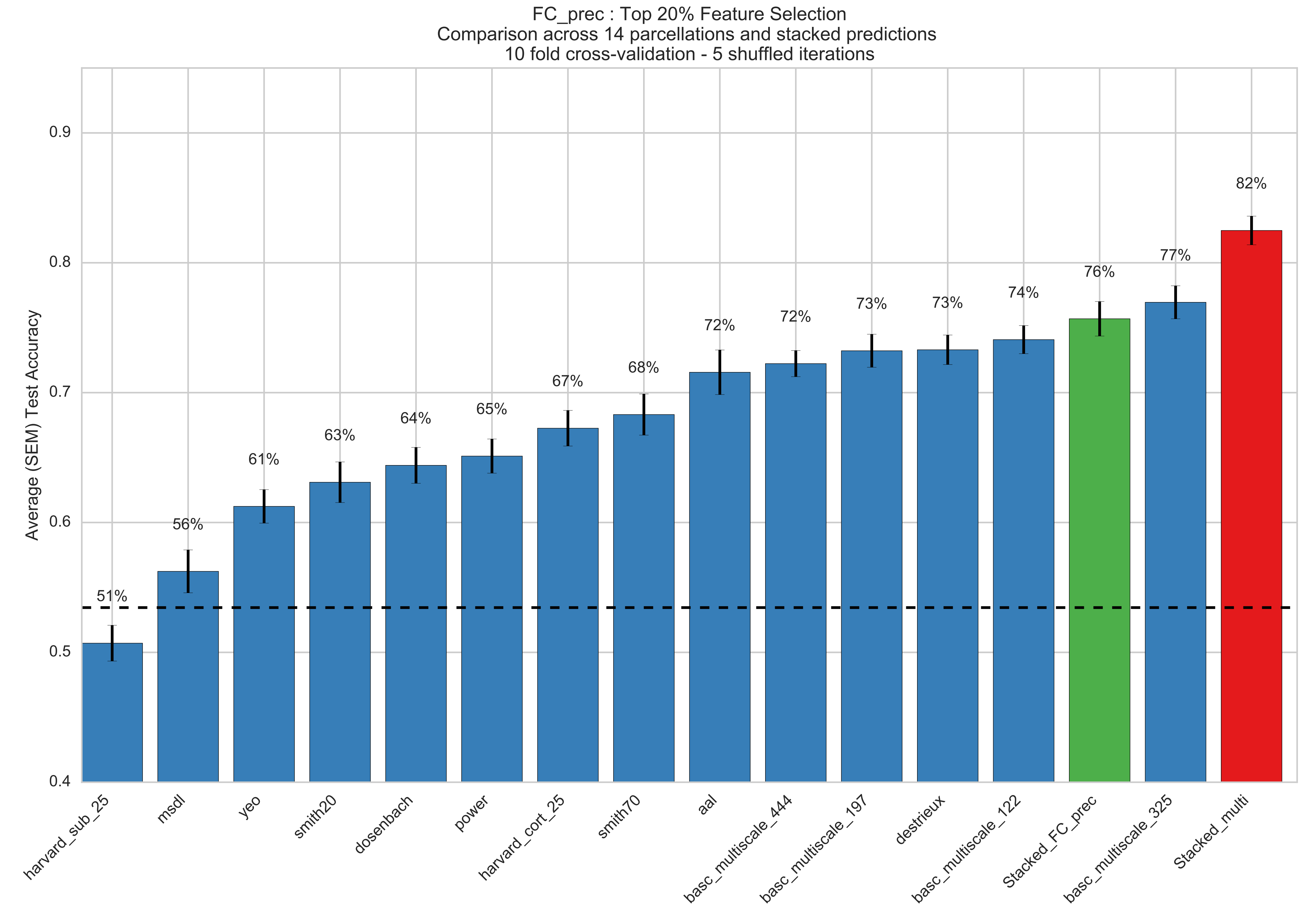

FC_prec : Top 20% Feature Selection
Comparison across 14 parcellations and stacked predictions
10 fold cross-validation - 5 shuffled iterations
Average (SEM) Test Accuracy
0.9
0.8
0.7
0.6
0.5
0.4
51%
56%
61%
63%
64%
65%
67%
68%
72%
72%
73%
73%
74%
76%
77%
82%
harvard_sub_25
msdl
yeo
smith20
dosenbach
power
harvard_cort_25
smith70
aal
basc_multiscale_444
basc_multiscale_197
destrieux
basc_multiscale_122
Stacked_FC_prec
basc_multiscale_325
Stacked_multi

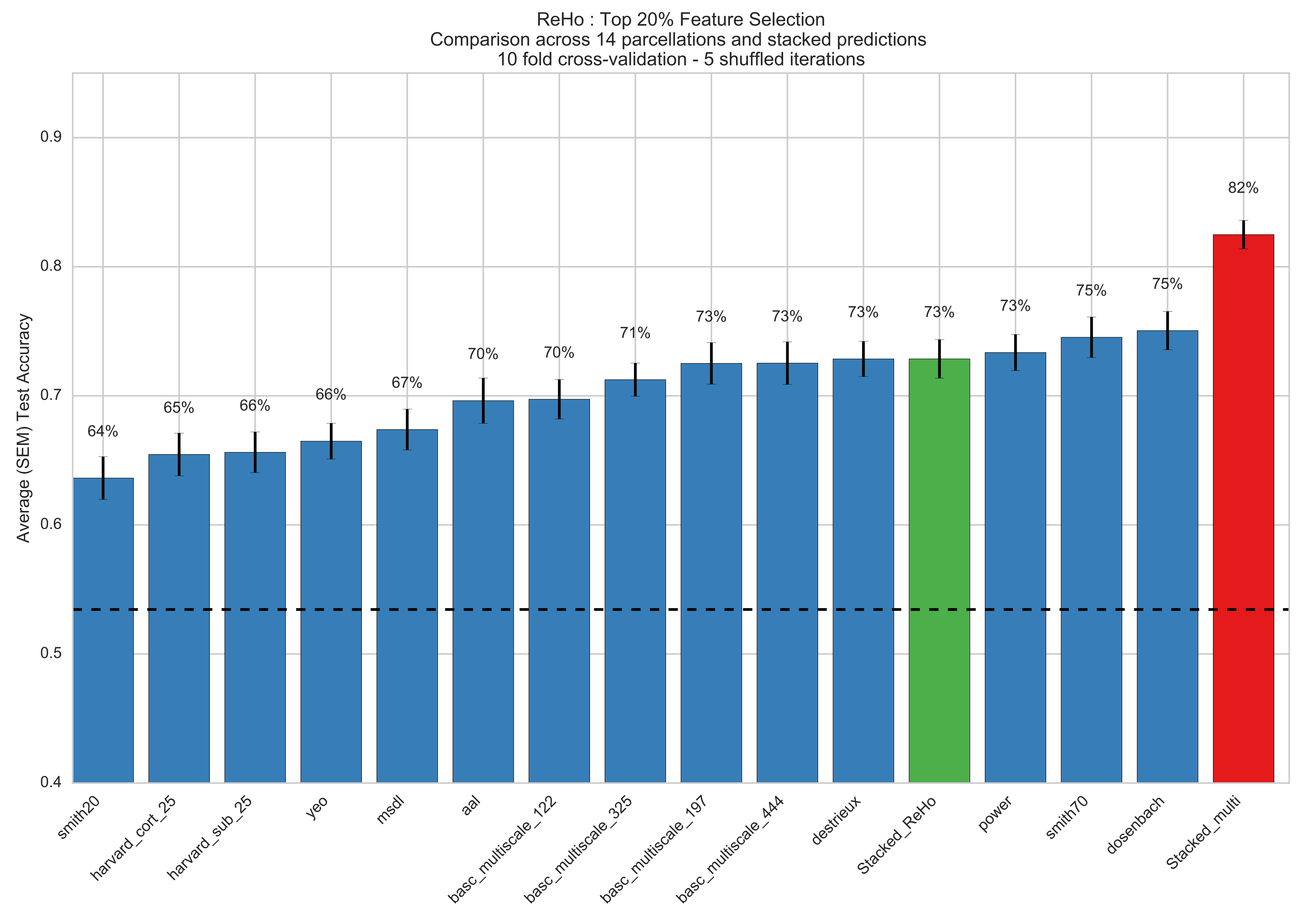

ReHo : Top 20% Feature Selection
Comparison across 14 parcellations and stacked predictions
10 fold cross-validation - 5 shuffled iterations
Average (SEM) Test Accuracy
0.4
0.5
0.6
0.7
0.8
0.9
64%
65%
66%
66%
67%
70%
70%
71%
73%
73%
73%
73%
73%
75%
75%
82%
smith20
harvard_cort_25
harvard_sub_25
yeo
msdl
aal
basc_multiscale_122
basc_multiscale_325
basc_multiscale_197
basc_multiscale_444
destrieux
Stacked_ReHo
power
smith70
dosenbach
Stacked_multi

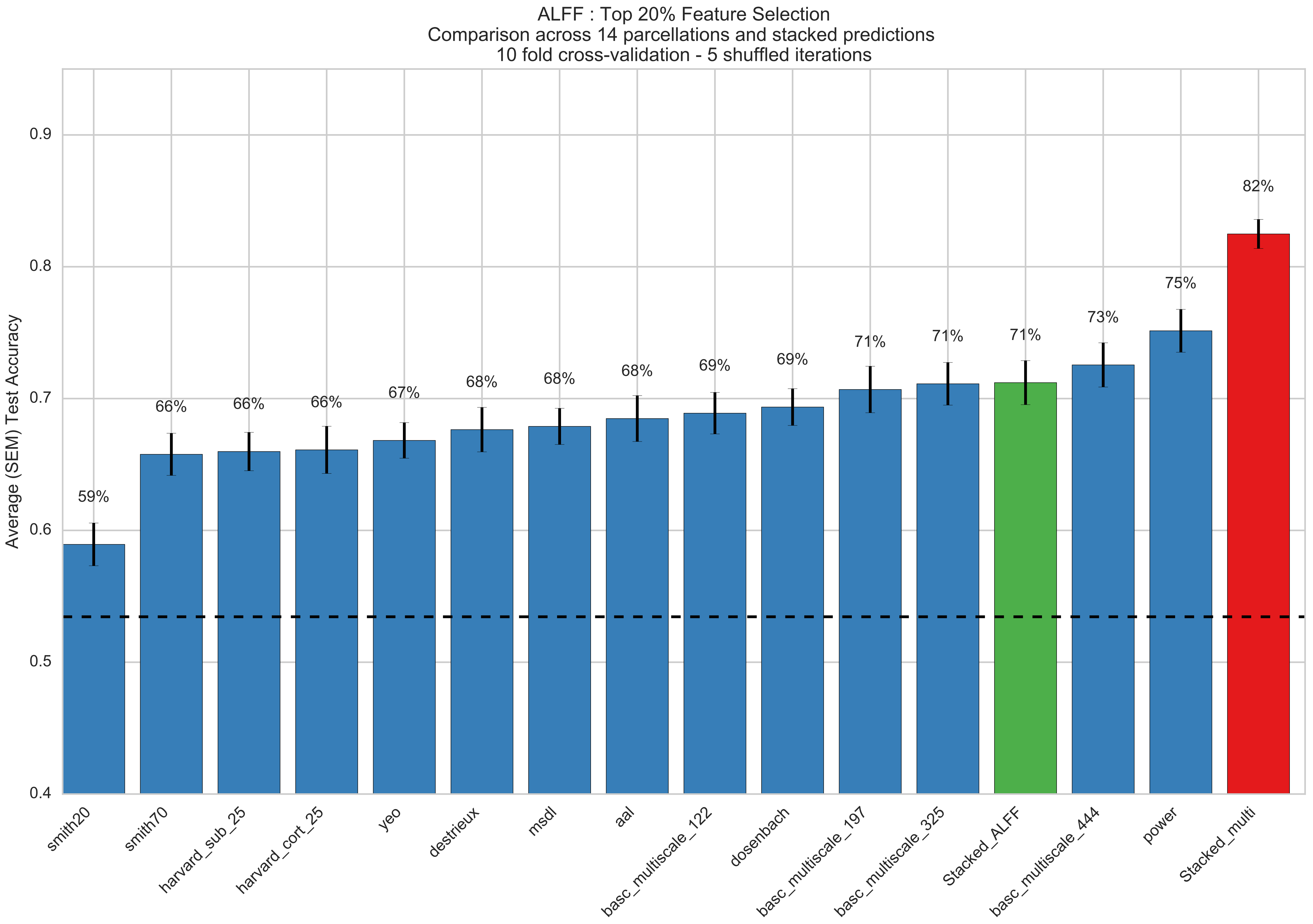

ALFF : Top 20% Feature Selection
Comparison across 14 parcellations and stacked predictions
10 fold cross-validation - 5 shuffled iterations
Average (SEM) Test Accuracy
0.4
0.5
0.6
0.7
0.8
0.9
59%
66%
66%
66%
67%
68%
68%
68%
69%
69%
71%
71%
71%
73%
75%
82%
smith20
smith70
harvard_sub_25
harvard_cort_25
yeo
destrieux
msdl
aal
basc_multiscale_122
dosenbach
basc_multiscale_197
basc_multiscale_325
Stacked_ALFF
basc_multiscale_444
power
Stacked_multi

Feature selection / reduction method :100% Features

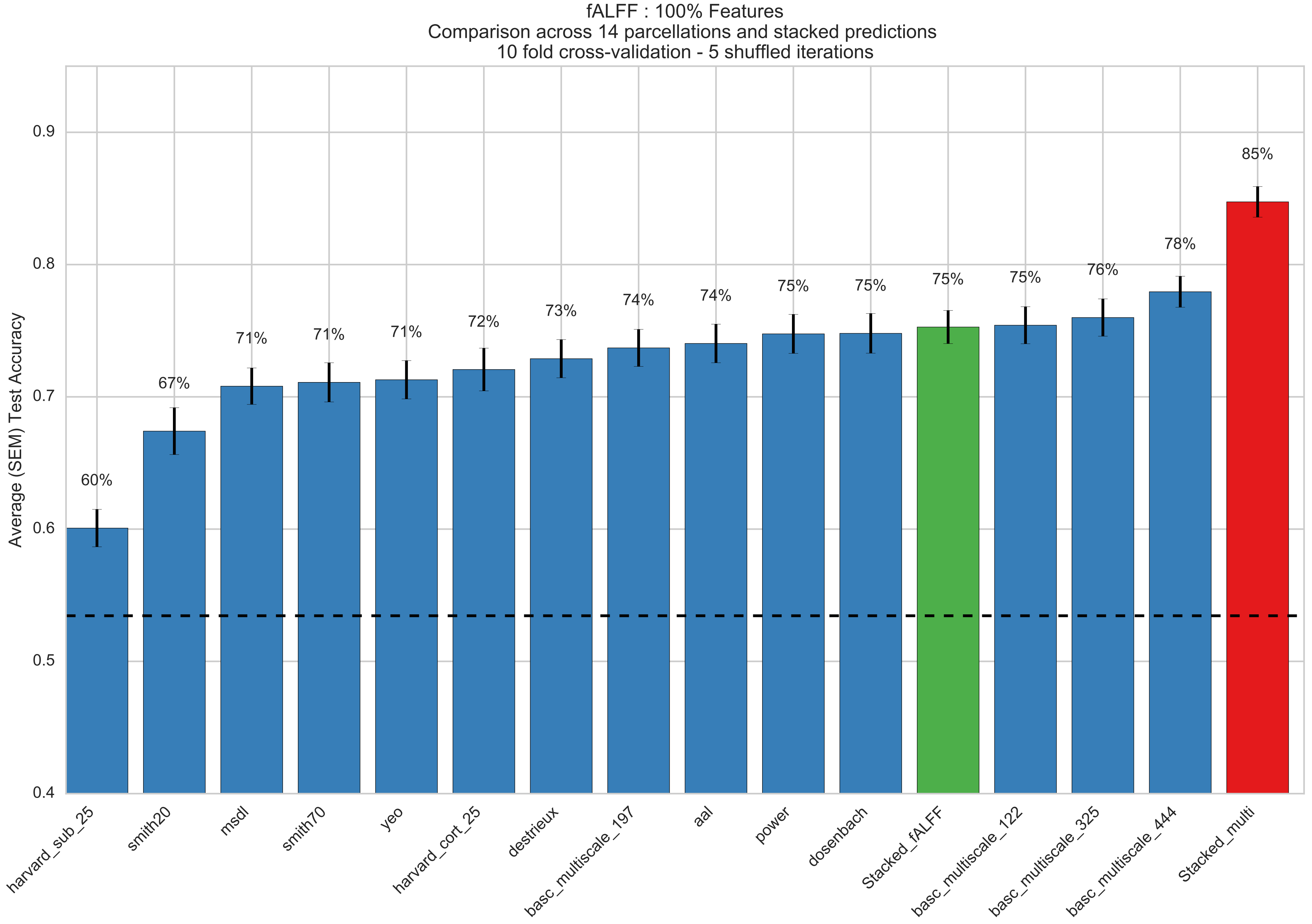
fALFF : 100% Features
Comparison across 14 parcellations and stacked predictions
10 fold cross-validation - 5 shuffled iterations
Average (SEM) Test Accuracy
0.4
0.5
0.6
0.7
0.8
0.9
60%
67%
71%
71%
71%
72%
73%
74%
74%
75%
75%
75%
75%
76%
78%
85%
harvard_sub_25
smith20
msdl
smith70
yeo
harvard_cort_25
destrieux
basc_multiscale_197
aal
power
dosenbach
Stacked_fALFF
basc_multiscale_122
basc_multiscale_325
basc_multiscale_444
Stacked_multi

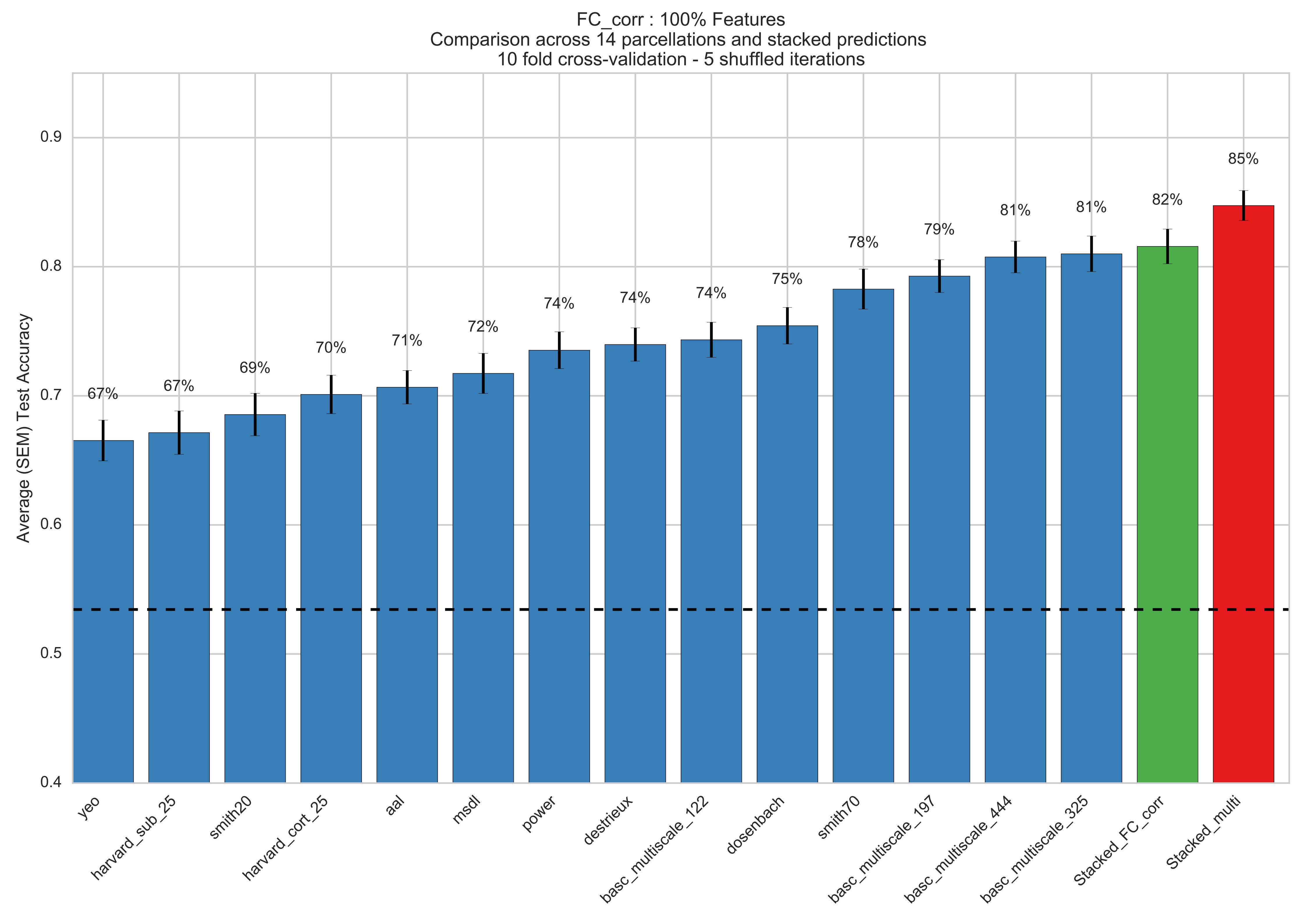
FC_corr : 100% Features
Comparison across 14 parcellations and stacked predictions
10 fold cross-validation - 5 shuffled iterations
Average (SEM) Test Accuracy
0.4
0.5
0.6
0.7
0.8
0.9
67%
67%
69%
70%
71%
72%
74%
74%
74%
75%
78%
79%
81%
81%
82%
85%
yeo
harvard_sub_25
smith20
harvard_cort_25
aal
msdl
power
destrieux
basc_multiscale_122
dosenbach
smith70
basc_multiscale_197
basc_multiscale_444
basc_multiscale_325
Stacked_FC_corr
Stacked_multi

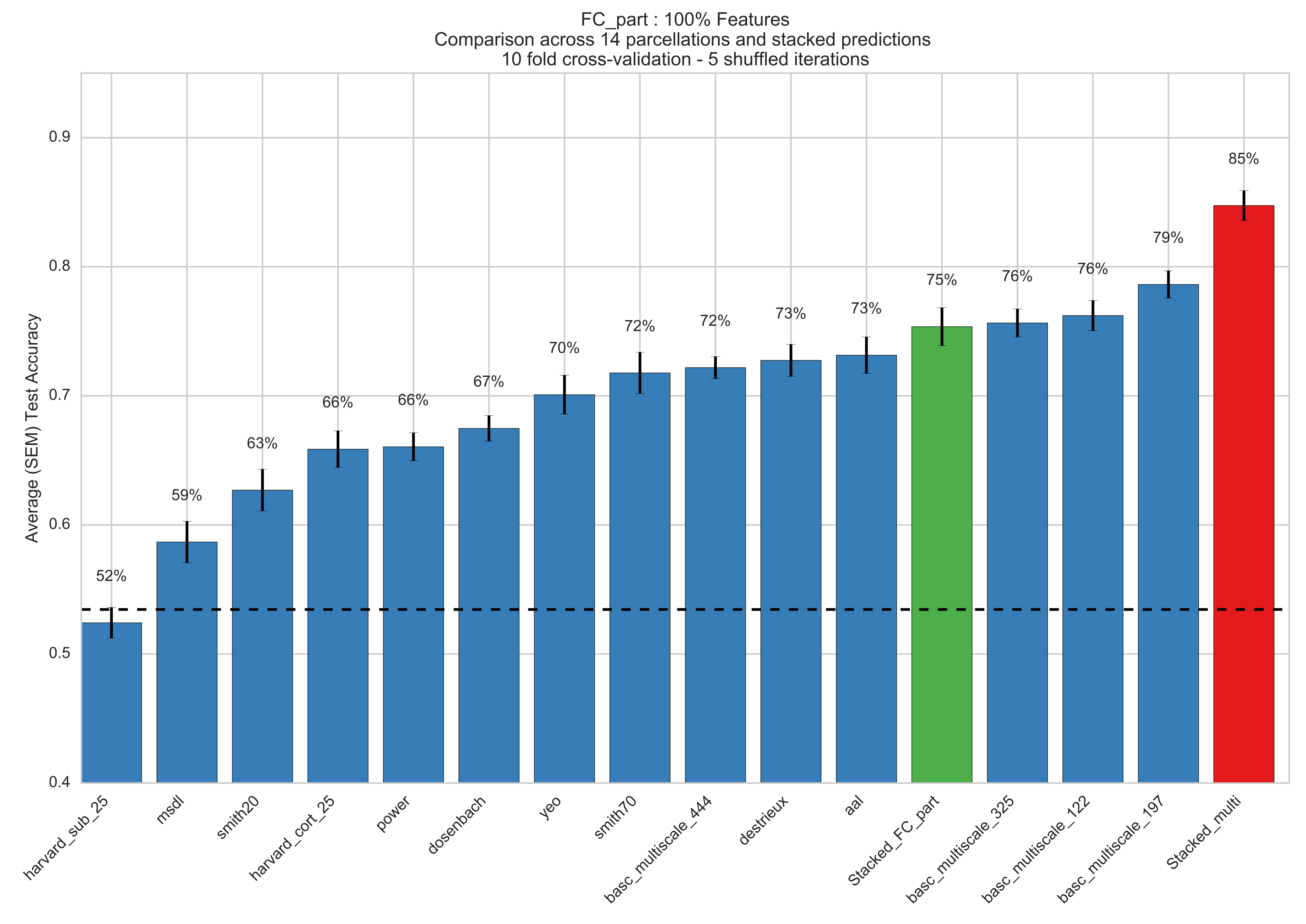
FC_part : 100% Features
Comparison across 14 parcellations and stacked predictions
10 fold cross-validation - 5 shuffled iterations
Average (SEM) Test Accuracy
0.4
0.5
0.6
0.7
0.8
0.9
52%
59%
63%
66%
66%
67%
70%
72%
72%
73%
73%
75%
76%
76%
79%
85%
harvard_sub_25
msdl
smith20
harvard_cort_25
power
dosenbach
yeo
smith70
basc_multiscale_444
destrieux
aal
Stacked_FC_part
basc_multiscale_325
basc_multiscale_122
basc_multiscale_197
Stacked_multi

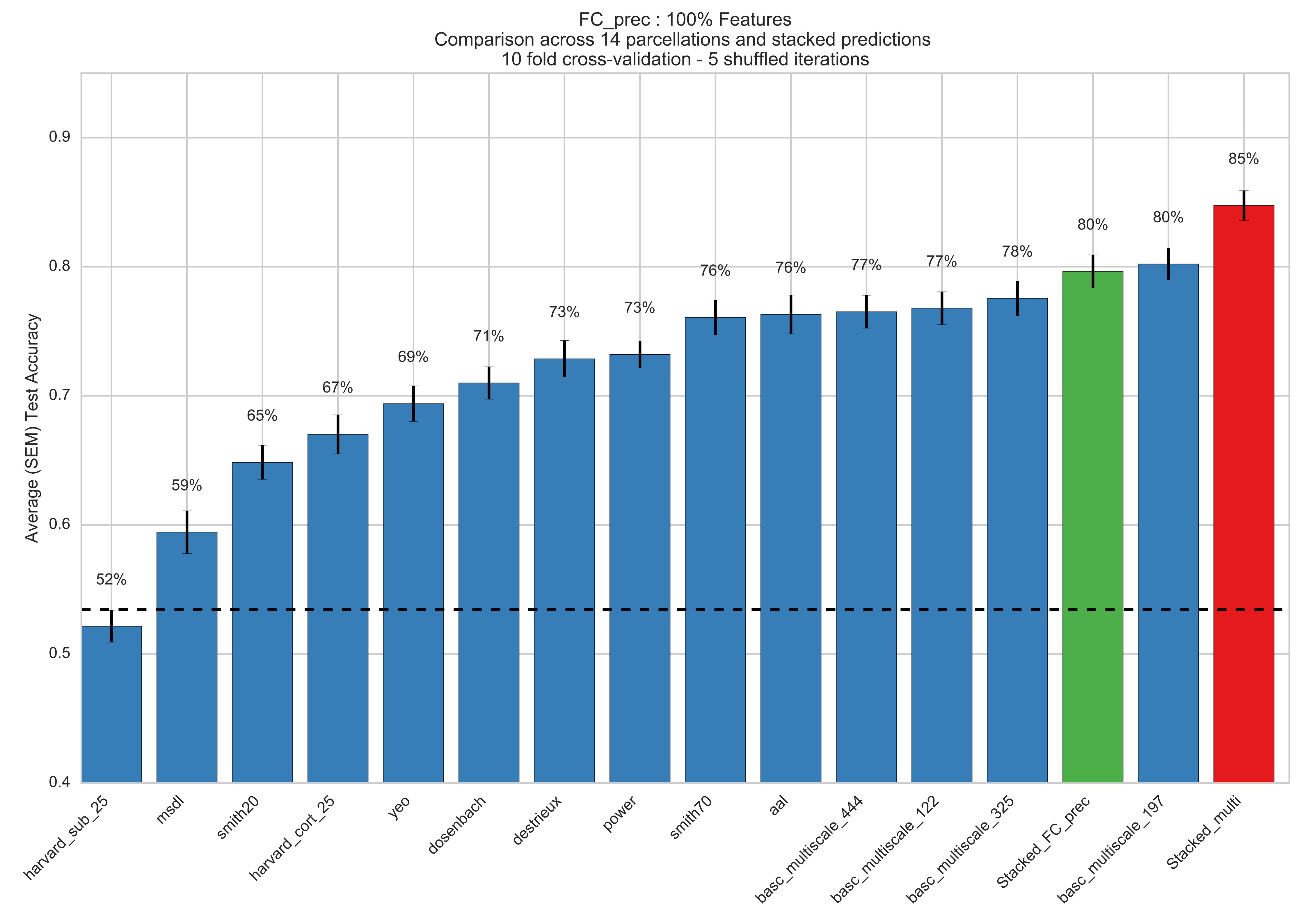

FC_prec : 100% Features
Comparison across 14 parcellations and stacked predictions
10 fold cross-validation - 5 shuffled iterations
Average (SEM) Test Accuracy
0.4
0.5
0.6
0.7
0.8
0.9
52%
59%
65%
67%
69%
71%
73%
73%
76%
76%
77%
77%
78%
80%
80%
85%
harvard_sub_25
msdl
smith20
harvard_cort_25
yeo
dosenbach
destrieux
power
smith70
aal
basc_multiscale_444
basc_multiscale_122
basc_multiscale_325
Stacked_FC_prec
basc_multiscale_197
Stacked_multi

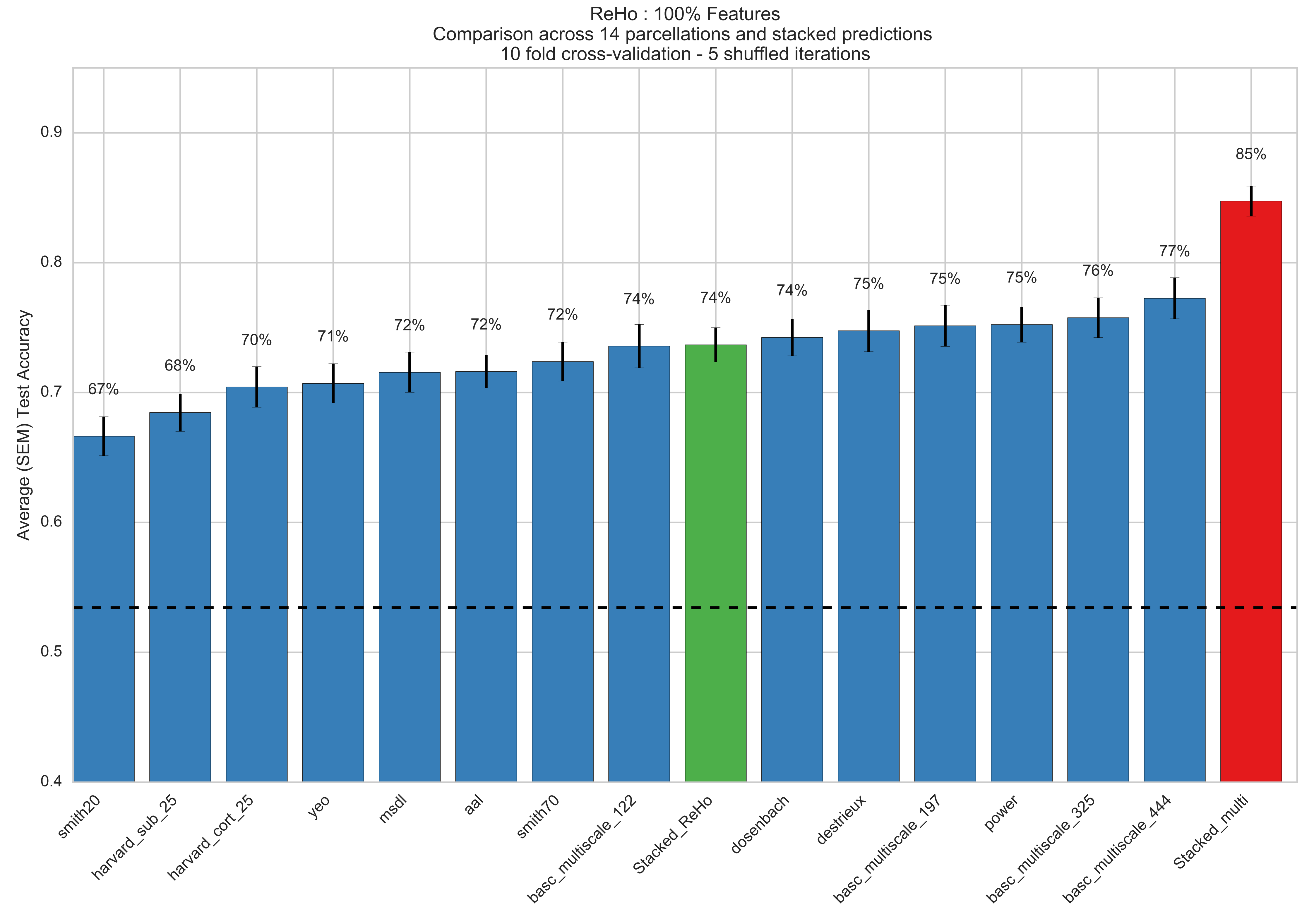

ReHo : 100% Features
Comparison across 14 parcellations and stacked predictions
10 fold cross-validation - 5 shuffled iterations
Average (SEM) Test Accuracy
0.9
0.8
0.7
0.6
0.5
0.4
67%
68%
70%
71%
72%
72%
72%
74%
74%
74%
75%
75%
75%
76%
77%
85%
smith20
harvard_sub_25
harvard_cort_25
yeo
msdl
aal
smith70
basc_multiscale_122
Stacked_ReHo
dosenbach
destrieux
basc_multiscale_197
power
basc_multiscale_325
basc_multiscale_444
Stacked_multi

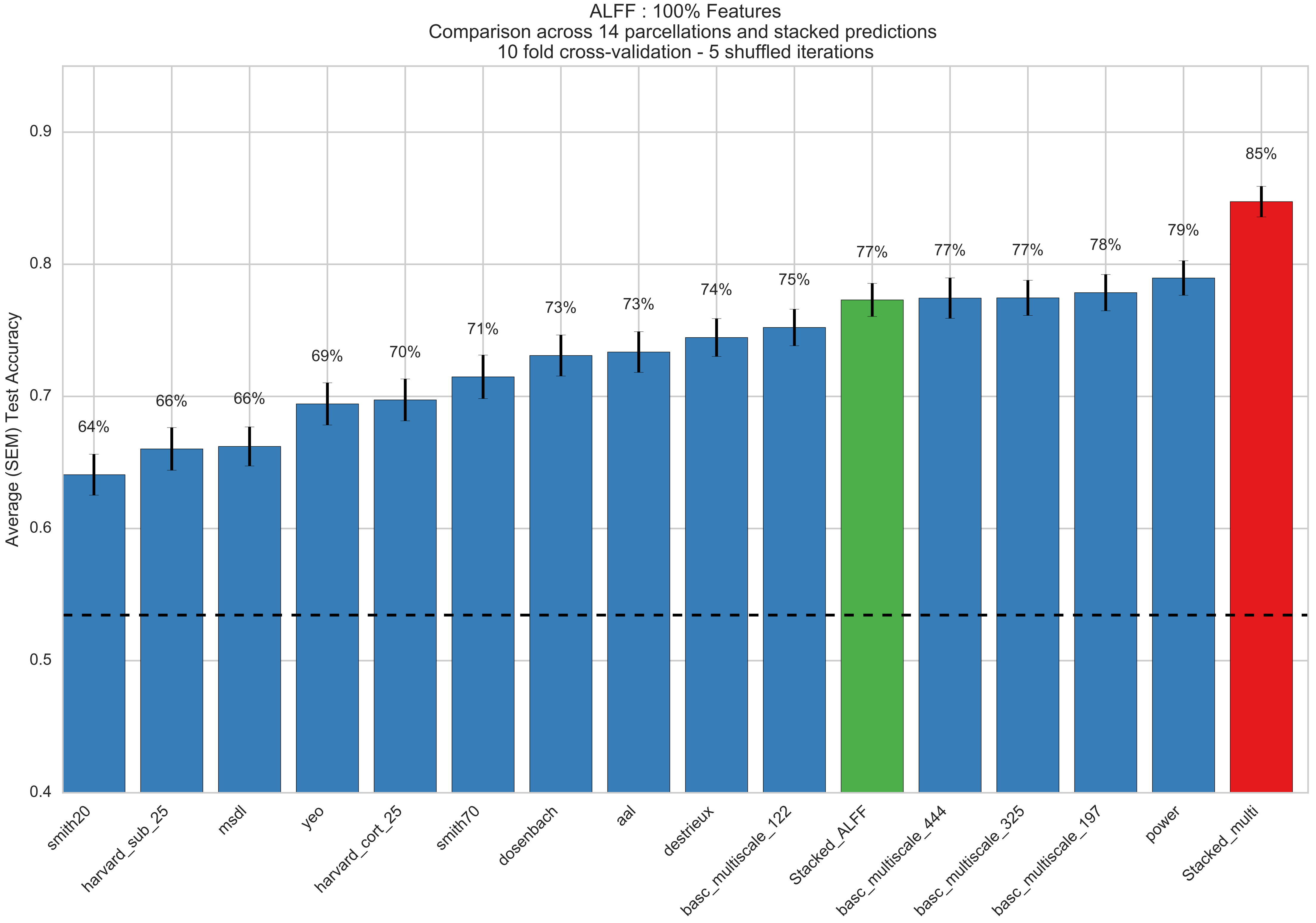

ALFF : 100% Features
Comparison across 14 parcellations and stacked predictions
10 fold cross-validation - 5 shuffled iterations
Average (SEM) Test Accuracy
0.4
0.5
0.6
0.7
0.8
0.9
64%
66%
66%
69%
70%
71%
73%
73%
74%
75%
77%
77%
77%
78%
79%
85%
smith20
harvard_sub_25
msdl
yeo
harvard_cort_25
smith70
dosenbach
aal
destrieux
basc_multiscale_122
Stacked_ALFF
basc_multiscale_444
basc_multiscale_325
basc_multiscale_197
power
Stacked_multi
